# Immunological characteristics of bronchoalveolar lavage fluid and blood across connective tissue disease-associated interstitial lung diseases

**DOI:** 10.3389/fimmu.2024.1408880

**Published:** 2024-10-25

**Authors:** Aiko Hirano, Aki Sakashita, Wataru Fujii, Kevin Baßler, Taisuke Tsuji, Masatoshi Kadoya, Atsushi Omoto, Noriya Hiraoka, Tatsuya Imabayashi, Yoshiko Kaneko, Hideaki Sofue, Yosuke Maehara, Takahiro Seno, Makoto Wada, Masataka Kohno, Wataru Fukuda, Kei Yamada, Koichi Takayama, Yutaka Kawahito

**Affiliations:** ^1^ Inflammation and Immunology, Graduate School of Medical Science, Kyoto Prefectural University of Medicine, Kyoto, Japan; ^2^ Aimed Analytics GmbH, Bonn, Germany; ^3^ Department of Respiratory Medicine, Japanese Red Cross Kyoto Daiichi Hospital, Kyoto, Japan; ^4^ Center for Rheumatic Disease, Japanese Red Cross Kyoto Daiichi Hospital, Kyoto, Japan; ^5^ Department of Pulmonary Medicine, Graduate School of Medical Science, Kyoto Prefectural University of Medicine, Kyoto, Japan; ^6^ Department of Radiology, Graduate School of Medical Science, Kyoto Prefectural University of Medicine, Kyoto, Japan

**Keywords:** single-cell RNA sequencing, genetics, interstitial lung disease, connective tissue disease, systemic autoimmune rheumatic disease

## Abstract

Interstitial lung disease (ILD) is a serious complication of connective tissue diseases (CTDs). The heterogeneity of ILDs reflects differences in pathogenesis among diseases. This study aimed to clarify the characteristics of CTD-ILDs via a detailed analysis of the bronchoalveolar lavage fluid (BALF) and blood immune cells. BALF and blood samples were collected from 39 Japanese patients with newly diagnosed ILD: five patients with Sjögren’s syndrome (SS), eight patients with dermatomyositis (DM), six patients with rheumatoid arthritis (RA), six patients with systemic sclerosis, four patients with anti-neutrophil cytoplasmic antibody-associated vasculitis, and 10 patients with idiopathic interstitial pneumonia. We performed single-cell RNA sequencing to analyze the gene expression profiles in these patients’ immune cells. In patients with SS, B cells in the BALF were increased and genes associated with the innate and acquired immunity were enriched in both the BALF and blood. In contrast, patients with DM showed an upregulation of genes associated with viral infection in both the BALF and blood. In patients with RA, neutrophils in the BALF tended to increase, and their gene expression patterns changed towards inflammation. These disease-specific characteristics may help us understand the pathogenesis for each disease and discover potential biomarkers.

## Introduction

Connective tissue disease-associated interstitial lung disease (CTD-ILD) is a serious complication and an important prognostic factor in various autoimmune diseases, such as Sjögren’s syndrome (SS), dermatomyositis (DM), rheumatoid arthritis (RA), systemic sclerosis (SSc), and anti-neutrophil cytoplasmic antibody (ANCA)-associated vasculitis (AAV) (1). The presentation and clinical course of CTD-ILDs differ depending on the underlying CTD (2). In addition, the heterogeneity in the lung fibrosis status in these diseases may reflect differences in the underlying pathogenic mechanisms, which may involve multiple cellular compartments (3). Various immune cells are thought to be involved in the pathogenesis of CTD-ILD (4); however, the characteristics of immune cells in each underlying disease remain unclear.

High-resolution computed tomography is essential for the diagnosis and severity assessment of CTD-ILDs (5). However, even with similar diagnosis and severity, some patients may face a poorer prognosis or experience acute exacerbations (1). Therefore, advanced techniques beyond imaging help better understand the diversity and pathophysiology of CTD-ILDs. Bronchoalveolar lavage fluid (BALF) is a useful sample to help exclude infectious diseases and alveolar hemorrhage and can provide complementary clues for diagnosis (5–7). Although histopathology seems to provide more information than BALF samples, the necessity of lung biopsy for CTD-ILD is controversial because of its invasiveness; therefore, its practice is limited (8, 9). If we could interpret the local pathology of the lung from the status of immune cells in BALF, it would be useful to further understand the pathogenesis of CTD-ILDs. The phenotypic characteristics of immune cells in the BALF of patients with CTD-ILD are not well known, so a comprehensive landscape of immune cells in the BALF is necessary to identify features reflecting the pathogenesis of each CTD-ILD. Single-cell RNA sequencing technologies are revolutionary and can potentially define cell populations more accurately by examining a large number of genes (10–12). Another example of a single-cell transcriptome study is the report of specific disease-related functional changes in lung macrophages (13–16). Hence, single-cell transcriptomic analysis appears promising for revealing the characteristics of immune cells in CTD-ILDs.

Therefore, in this study, we used single-cell RNA sequencing to analyze the characteristics of immune cells in BALF and blood samples from patients with newly developed CTD-ILD. We aimed to clarify the pathogenesis of each CTD-ILD and investigate their characteristics in terms of the distribution of immune cells and their gene expression profiles in the BALF and blood.

## Materials and methods

### Summary of materials and methods

The scRNA-seq analysis encompassed several crucial steps. Fastq files were preprocessed using the Drop-seq tools and aligned with the hg38 reference genome, excluding abundant mitochondrial transcripts. Quality control involved defining inclusion criteria based on gene and cell counts, and filtering cells with high endogenous-to-mitochondrial counts. The Seurat pipeline was employed for dataset integration, log-normalization, variable gene selection, and dimensionality reduction. Harmony integration mitigated batch effects, and UMAP representation was generated. Doublet cells were identified using the ‘DoubletFinder’ package.

To identify and remove low-quality cells, various metrics were collected, including mitochondrial gene counts, empty droplets, ribosomal reads, and cell-type annotations. This resulted in a blood dataset of 35,670 cells and a BALF dataset of 83,067 cells across 14,666 genes. Patient 25 in the blood dataset was excluded due to contamination with BALF cells. Patients 4, 10, 36, and 45 were also excluded from the blood dataset due to the low number of reads. Patients 1, 10, 16, and 31 were included in the cell type annotations of BALF and blood but were excluded from the further analysis. Patients 1 and 16 were excluded because their radiological patterns were OP, making the diagnosis of idiopathic interstitial pneumonia (IIP) controversial. Patient 10 was excluded because of taking 2g/day of mycophenolate mofetil. and Patient 31 was excluded because of the diagnosis other than CTD-ILDs. Detailed patient information is provided in **Supplementary Table 1**. Clustering was performed using an SNN-graph algorithm, and cell types were annotated using ‘FindTransferAnchors’ and ‘MapQuery’ functions. Sub-clustering of major cell types was carried out, and DE analysis was conducted at both major cell type and sub-cluster levels. Two strategies, involving ‘DESeq2’ and ‘IDEAS,’ were used for DE gene identification. Gene ontology enrichment analysis was performed, and data were visualized using Seurat, pheatmap, and ggplot2.

### Study population

Human studies were approved by the ethics committee of the Kyoto Prefectural University of Medicine (approval number ERB-C-1471) and conducted in accordance with the Declaration of Helsinki. All patients provided written informed consent before specimens were collected. Patients with SS, DM, RA, SSc, AAV, or IIP were diagnosed according to each classification criteria (17–24). Radiological findings examined using high-resolution computed tomography (HRCT) were diagnosed and classified by a thoracic radiologist according to the American Thoracic Society/European Respiratory Society (ATS/ERS) classification of IIP (17). Tables explaining the clinical characteristics, demographic characteristics, and smoking habits are presented in **Table 1** and **Supplementary Table 1**. At the onset of interstitial pneumonia, one patient with RA was taking 5 mg/day prednisolone equivalent for other symptoms.

**Table 1:** Patient characteristics.

	SS	DM	RA	SSc	AAV	IIP	p
n	5	8	6	6	4	10	
Radiological pattern, n(%)							0.0040
NSIP	3 (60)	7 (88)	4 (67)	5 (83)	0 (0)	6 (60)	
UIP	1 (20)	0 (0)	0 (0)	1 (17)	1 (25)	3 (30)	
OP	0 (0)	1 (13)	2 (33)	0 (0)	0 (0)	0 (0)	
HP	1 (20)	0 (0)	0 (0)	0 (0)	0 (0)	0 (0)	
PPFE	0 (0)	0 (0)	0 (0)	0 (0)	0 (0)	1 (10)	
Unclassifiable	0 (0)	0 (0)	0 (0)	0 (0)	3 (75)	0 (0)	
Sex, female subjects, n(%)	5 (100)	3 (38)	2 (33)	6 (100)	2 (50)	2 (20)	0.0017
Age, median (IQR)	64 (55, 68)	58 (45, 60)	71 (67, 77)	68 (67, 73)	73 (70, 74)	72 (66, 75)	0.11
BMI, n(%)	19.6 (15.6, 23.9)	23.4 (20.6, 25.1)	25.5 (21.1, 27.6)	21.8 (21.4, 22.3)	24.4 (22.6, 26.5)	24.5 (23.1, 27.7)	0.24
Current smoking, n(%)	0 (0)	0 (0)	0 (0)	0 (0)	1 (25)	2 (20)	0.27
EX-smoking, n(%)	0 (0)	2 (25)	4 (67)	1 (17)	2 (50)	1 (10)	0.0013
Pack-years, median (IQR)	0 (0, 0)	0 (0, 30.0)	10.6 (0, 34.0)	0 (0, 0)	20.0 (0, 48.0)	44.5 (28.5, 65.0)	0.0013
VC (% predicted), median (IQR)	72.8 (71.9, 82.4)	68.3 (68.0, 76.3)	79.5 (73.4, 83.0)	92.2 (89.7, 100.2)	78.6 (75.6, 81.7)	76.8 (67.4, 90.2)	0.11
Blood							
WBC, /μL, median (IQR)	5100 (4810, 5660)	5300 (4900, 7320)	9620 (8420, 10100)	4990 (4080, 6500)	7980 (5940, 11100)	6560 (5020, 7370)	0.18
lymphocyte, /μL, median (IQR)	1000 (800, 1100)	1590 (1420, 1830)	1730 (1380, 2070)	1600 (1050, 2150)	1270 (1010, 1700)	1650 (1500, 2150)	0.35
neutrophils, /μL, median (IQR)	3400 (2590, 4300)	3590 (2740, 4450)	6750 (5920, 7280)	3150 (2580,3880)	5470 (3500, 8750)	3850 (2880, 4530)	0.13
monocytes, /μL, median (IQR)	300 (300, 350)	500 (355, 555)	565 (473, 600)	400 (325, 475)	455 (275, 733)	550 (400, 600)	0.39
eosinophils, /μL, median (IQR)	100 (70.0, 100)	120 (55.0, 235)	150 (75.0, 225)	150 (100, 200)	150 (100, 253)	250 (125, 375)	0.48
CRP, mg/dL, median (IQR)	0.130 (0.100, 0.280)	0.275 (0.0925, 0.703)	3.55 (2.06, 5.82)	0.265 (0.0925, 0.460)	1.09 (0.135, 5.27)	0.500 (0.0875, 0.880)	0.083
KL-6, U/mL, median (IQR)	1830 (1120, 2780)	983 (714, 1870)	876 (410, 2270)	866 (531, 1130)	326 (237, 548)	960 (530, 1310)	0.19
BALF							
BALF cell numbers, /μL, median (IQR)	163 (135, 373)	200 (150, 216)	134 (55.3, 255)	124 (95.0, 138)	44.5 (37.8, 160)	84.5 (61.5, 171)	0.27
%neutrophils, %, median (IQR)	7.00 (1.00, 15.0)	2.00 (1.50, 4.00)	6.00 (5.00, 42.5)	0.50 (0.00, 1.75)	4.50 (1.00, 8.25)	1.00 (0.00, 1.75)	0.060
%lymphocytes, %, median (IQR)	28.0 (8.00, 15.0)	25.0 (11.5, 48.5)	3.00 (1.50, 4.50)	5.00 (4.25, 11.0)	3.50 (2.25, 5.00)	2.50 (1.25, 7.00)	0.016
%eosinophils, %, median (IQR)	1.00 (0.00, 4.00)	1.00 (0.00, 3.50)	3.00 (1.50, 3.00)	0.50 (0.00, 1.00)	0.50 (0.00, 1.25)	0.00 (0.00, 0.00)	0.56
%macrophages, %, median (IQR)	61.0 (40.0, 73.0)	60.0 (47.5, 83.0)	91.4 (71.3, 93.5)	93.0 (87.5, 95.5)	91.0 (90.0, 91.8)	97.0 (88.3, 98.0)	0.052
CD4 / CD8	3.03 (1.48, 3.56)	0.272 (0.196, 0.307)	0.76 (0.55, 1.60)	1.41 (0.867, 4.07)	1.12 (1.18, 1.26)	2.50 (1.44, 4.41)	0.023
recovery rate, %, median (IQR)	51.3 (50.0, 56.7)	52.5 (38.6, 57.5)	51.7 (35.5, 55.8)	62.5 (56.3, 66.5)	62.5 (57.8, 68.0)	58.0 (51.0, 66.0)	0.14

The Kruskal–Wallis test was performed for the multi-condition comparison and Fisher’s exact test was performed for the categorical variables. Statistical significance was set at p < 0.05. The recovery rate was calculated by dividing the total volume of recovered BALF by the 150 ml of saline solution injected.

NSIP, Non-specific interstitial pneumonia; UIP, Usual interstitial pneumonia; OP, Organizing pneumonia; HP, hypersensitivity pneumonitis; PPFE, pleuroparenchymal fibroelastosis; SS, Sjögren’s syndrome; DM, dermatomyositis; RA, rheumatoid arthritis; SSc, systemic sclerosis; AAV, ANCA-associated vasculitis; IIP, idiopathic interstitial pneumonia.

### Bronchoscopy procedure

Bronchoscopy was performed as part of the diagnostic workup by two bronchoscopists through oral access and with light conscious sedation in the middle lobe or, if not accessible, the lingular lobe. BAL was conducted using the fiberoptic bronchoscope in a wedge position within the selected bronchopulmonary segment. Warmed saline solution (three syringes, 50 mL each) was injected into the airway with the intention of retrieving at least 30% of its total volume to obtain BALF specimens. The BALF was passed through sterile gauze and collected in containers for suspension tissue culture and the specimens were transported at 4°C (i.e., on ice).

### BALF processing

Human BALF was obtained from all patients included in the study through bronchoscopy. BALF specimens were centrifuged at 4°C and 300 × g for 10 minutes, and the supernatant was separated. Subsequently, they were washed with PBS supplemented with 2% fetal calf serum (FCS) and centrifuged at 4°C and 300 × g for 10 minutes. The supernatants were discarded, and the remaining cells were resuspended in 3 ml of PBS supplemented with 2% FCS, after which the cells were counted.

### Isolation of peripheral blood mononuclear cells and granulocytes

Peripheral blood mononuclear cell (PBMC) was obtained by Ficoll density centrifugation (at 20°C and 400 × g for 30 min with the centrifugation break turned off) of the peripheral blood. After harvesting PBMC from the interphase, all further steps were conducted at 4°C. Granulocytes were recovered from the granulocyte/erythrocyte fraction using cold ammonium chloride potassium lysing buffer (1.5M NH_4_Cl, 0.1M KHCO_3_, and 1mM EDTA in H_2_O with pH 7.4 at 8°C) to lyse erythrocytes, followed by a washing step with PBS supplemented with 2% FCS. All centrifugation steps required for granulocyte isolation were performed at max 300 × g for 10 min.

### Flow cytometry/FACS

Single-cell suspensions were stained with Fixable Viability Dye eFluor™ 780 (ThermoFisher, USA) for 15 min at room temperature and washed with PBS at 300 × g for 5 min at 4°C. They were then resuspended in 100 µL PBS and blocked with 5 µL human FcR blocking reagent (Miltenyi, Germany) for 15 min on ice and were subsequently stained with the listed anti-human antibodies (**Supplementary Table 2**) in buffer containing PBS, 2% FCS for 30 min on ice. The cells were centrifuged at 300 × g for 5 min at 4°C and re-suspended in a buffer containing PBS and 2% FCS for analysis. Data were acquired using a FACS Celesta (BD Biosciences). Data were analyzed using FlowJo v.10 software (Tree Star, USA). We gated CD45^+^ living single cells and analyzed myeloid cells and lymphoid cells separately. For myeloid cells in the BALF, we defined CD3^-^CD19^-^CD56^-^CD66b^+^HLA-DR^-^CD16^+^ cells as neutrophils, CD3^-^CD19^-^CD56^-^CD66b^+^HLA-DR^-^CD16^-^ as eosinophils, CD3^-^CD19^-^CD56^-^CD66b^-^HLA-DR^+^autofluorescence^+^ as alveolar macrophages, CD3^-^CD19^-^CD56^-^CD66b^-^HLA-DR^+^autofluorescence^-^CD14^+^ as monocytes, CD3^-^CD19^-^CD56^-^CD66b^-^HLA-DR^+^autofluorescence^-^CD14^-^ as dendritic cells, and CD3^+^CD19^+^CD56^+^ as lymphocytes (**Supplementary Figure 1A**). For myeloid cells in the blood, we defined CD3^-^CD19^-^CD56^-^CD66b^+^HLA-DR^-^CD16^+^ cells as neutrophils, CD3^-^CD19^-^CD56^-^CD66b^+^HLA-DR^-^CD16^-^ as eosinophils, CD3^-^CD19^-^CD56^-^CD66b^-^CD14^+^CD16^-^ as classical monocytes, CD3^-^CD19^-^CD56^-^CD66b^-^CD14^+^CD16^+^ as intermediate monocytes, CD3^-^CD19^-^CD56^-^CD66b^-^CD14^-^CD16^+^ as nonclassical monocytes, CD3^-^CD19^-^CD56^-^CD66b^-^HLA-DR^+^CD14^-^CD16^-^ as dendritic cells, and CD3^+^CD19^+^CD56^+^ as lymphocytes (**Supplementary Figure 1B**). For lymphoid cells in the blood and BALF, we defined CD3^+^CD19^-^ cells as T cells, CD3^-^CD19^+^ as B cells, CD3^-^CD19^-^CD56^+^ as CD56 NK cells, CD3^-^CD19^-^CD56^-^CD16^+^ as CD16 NK cells, CD3^+^CD4^+^CD8^-^CD19^-^ cells as CD4 T cells, CD3^+^CD4^-^CD8^+^CD19^-^ as CD8 T cells, and CD3^+^CD4^-^CD8^-^CD19^-^ as double negative T cells (**Supplementary Figures 1C, D**).

### Measurement of proteins in BALF and plasma

After the isolation of cells (see above), the supernatant of BALF samples and plasma was collected and frozen at −80°C before protein measurement. Protein levels in cell-free BALF and plasma samples were determined using the LEGENDplex macrophage/microglia panel (BioLegend, USA). The normalized results were further analyzed using the LEGENDplex software. Complement levels were estimated using an enzyme-linked immunosorbent assay (ELISA) kit (BD Biosciences), according to the manufacturer’s protocol.

### Analysis of immune cells in the BALF and blood of patients with CTD-ILD by nanodroplet-based scRNA-seq (Seq-Well)

Freshly isolated BALF and peripheral blood were collected from patients. In order to analyze the gene expression patterns of immune cells in the BALF and blood, we used Seq-Well, a nanodroplet-based technology for single-cell RNA sequencing (25). Briefly, individual cells were loaded in nanowells with capture beads. Seq-well is one of the commonly used methods for single-cell RNA sequencing (26) and shown to be comparable to other methods (14). Libraries were prepared using the Nextera XT DNA Sample Prep Kit (Illumina) according to the manufacturer’s recommendations, and paired-end sequencing was performed as follows: Read 1 26 cycles, i7 index 8 cycles and Read 2 56 cycles on a NextSeq500 instrument (Illumina) by Macrogen Japan (Tokyo, Japan). We then compared the distribution of immune cells and the differential gene expression profiles in the BALF and blood samples of patients.

### Preparation of Seq-Well arrays, libraries, and sequencing

Seq-Well arrays and libraries were prepared as described by Gierahn et al. (25). Briefly, Sylgard base and crosslinker were mixed in a ratio of 10:1 for 10 min, placed under vacuum pressure for 15 min to remove air bubbles and poured for a 2 h incubation at 70°C into a wafer with a mounted 86,000 well pattern-holding microscope slide. The arrays were then removed from the molds, excess silicone was cut off with a blade, and the arrays were prepared for functionalization. This protocol adds chemical moieties to the surface of the arrays, facilitating sealing with a semipermeable polycarbonate membrane and the interchange of lysis and RNA hybridization buffers. The arrays were rinsed with EtOH, plasma treated for 10 min, and successively submerged in APTES, acetone, and PDITC buffers. Upon further washes with acetone, the arrays were spun and dried at 70°C for 2 h. The arrays were then incubated with 0.2% chitosan solution (pH=6.3) at 37°C for 1.5 h, followed by overnight incubation in PGA buffer at room temperature under vacuum pressure. Finally, the arrays were removed from the vacuum, rotated for 3 h at room temperature, and subsequently moved to 4°C for at least 24 h before use.

After loading the functionalized arrays with mRNA capture beads, 20,000 cells were coated and suspended in RPMI 1640 medium supplemented with 10% FCS. During the 10 min incubation period, the loaded arrays were placed on a strong magnetic plate to support the settling of the cells *via* a magnetic field. After repeated washing with PBS and soaking in RPMI 1640 medium, the arrays were sealed using polycarbonate membranes treated with air plasma for 7 min under mild vacuum (Diener Electronic). Following a 30 min incubation in a 37°C cell culture incubator, the arrays were incubated in lysis buffer for 20 min and then placed in hybridization buffer for 40 min. Next, the mRNA capture beads were washed from the arrays and collected using washing buffer. Reverse transcription was performed on the bead pellet using a Maxima Reverse Transcriptase reaction for 30 min at room temperature followed by 90 min incubation at 52°C with end-over-end rotation. The reaction was stopped by washing the beads with TE buffer supplemented with 0.1% Tween-20 (TE-TW) and TE buffer supplemented with 0.5% SDS (TE-SDS). After a washing step in 10mM TrisHCl pH 8.0, excess primers were digested in an exonuclease reaction for 50 min at 37°C with end-over-end rotation and washed in TE-TW and TE-SDS. Beads were resuspended in 500 µL H_2_O and counted using a Fuchs-Rosenthal cytometer in bead counting solution. Pools of 5,000 beads (10 µL) were then added to 40 µL PCR reactions for the amplification of reverse transcribed cDNA libraries. After PCR, 16,000-20,000 beads were combined (hereafter referred to as ‘pools’) and further processed. The pools were cleaned with 0.6 × volumetric ratio AMPure XP beads, and library integrity was assessed using the High Sensitivity D5000 ScreenTape assay for Tapestation 4200 (Agilent).

cDNA libraries (1 ng) were tagged using the Nextera XT DNA Sample Prep Kit (Illumina) according to the manufacturer’s recommendations. The pools were cleaned with 0.8 × volumetric ratio AMPure XP beads, run with a High-Sensitivity DNA5000 assay on Tapestation 4200 (Agilent), and quantified using the Qubit high-sensitivity dsDNA assay. Seq-Well libraries were equimolarly pooled and clustered at 1.4 pM concentration with 10% PhiX using High Output v2.1 chemistry on a NextSeq500 system. Paired-end sequencing was performed as follows: custom Drop-Seq Read 1 primer for 21 cycles, 8 cycles for the i7 index, and 61 cycles for Read 2. Single-cell data were demultiplexed using bcl2fastq2 (v2.20). See **Supplementary Table 3** for details on reagents and reactions.

### Preprocessing of scRNA-seq raw data

For preprocessing, the generated fastq files from Seq-Well were loaded into a data preprocessing pipeline (version 0.4, available at https://github.com/Hoohm/dropSeqPipe) which relies on Drop-seq tools provided by the McCarroll lab. STAR alignment within the pipeline was performed using the human reference genome (hg38) with annotations (Ensemble v91). The resulting datasets were imported into the R software for further analysis. The highly abundant mitochondrial transcripts *MT-RNR1* and *MT-RNR2* were excluded. The resulting datasets were imported into the R package ‘Seurat’ for downstream analyses. An overview of the used packages and package versions is provided in **Supplementary Table 4**.

### Quality control of scRNA-seq data

We selected cells and genes for further analyses using the following criteria for each donor separately: (і) only genes that were found in at least 100 cells were retained; (ii) a threshold of 300 expressed genes was used to keep cells for further analyses; and (iii) with regard to the rate of endogenous-to-mitochondrial counts per cell, cells with a rate of > 5% were excluded.

### Dataset integration and dimensionality reduction of scRNA-seq data

All the subsequent steps were conducted using the single-cell analysis pipeline Seurat unless stated otherwise. To account for variations in the sequencing depth across cells, we applied a log-normalization strategy using CPM normalization with a scale factor of 10,000. Next, the genes with the highest cell-to-cell variability in the dataset were determined by calculating the top 2,000 most variable genes using the ‘vst’ method of the ‘FindVariableFeatures’ function in Seurat.

After the linear transformation of the remaining genes (scaling) to ensure homoscedasticity, the dimensionality of the data was reduced to 30 principal components. To analyze the data without having any influence of batch effects resulting from either different donors or technologies, the ‘harmony’ integration approach based on patient batches was used to harmonize and integrate the different datasets using the Seurat implementation with the default settings. The integrated dataset was then used as the input for UMAP representation.

Next, doublet cells were identified utilizing the R package ‘DoubletFinder’ (version 2.0.2) (27) using the first 30 principal components of the non-integrated datasets, assuming a doublet formation rate of 10% and leaving all other parameters unaltered.

### Background identification and removal

To detect low-quality cells (background in the Seq-Well technology) and exclude them from further analysis, we collected the following rich set of information about the cells: the proportion of reads mapped to mtDNA using the ‘miQC’ package, the likelihood that a cell represents an empty droplet using the ‘emptyDrops’ function of ‘DropletUtils’ (FDR <= 0.2), the percentage of ribosomal reads, total reads, and the number of genes per cell. Additionally, we used the ‘perCellQCMetrics’ function of the ‘scater’ package and provided mitochondrial and ribosomal genes to detect the top 5% of cells enriched for these features. Another layer of information was provided using annotated datasets to query the most likely cell-type annotation of the cells in the datasets. For this purpose, we used the ‘FindTransferAnchors’ (reference reduction = PCA with 30 dimensions and log normalization) and the ‘MapQuery’ function of the ‘Seurat’ package. As reference samples, we used a large annotated PBMC dataset (https://www.cell.com/cell/fulltext/S0092-8674%2821%2900583-3) and the COPD dataset from Baßler et al. (14). Through this annotation, we obtained a mapping and prediction score that indicated how likely the cells in the reference dataset were to find a counterpart in the dataset used in this study. For each metric, we calculated the mean per cluster, ranked the cluster means from low to high quality, and combined all the statistics using the Borda rank. Clusters with exceptionally high numbers of low-quality hits were excluded from further analysis.

### Clustering of the integrated scRNA-seq datasets

The cellular heterogeneity of the integrated datasets was determined using a shared nearest neighbor (SNN)-graph based clustering algorithm implemented in the Seurat pipeline. For both the BALF and the blood data, we used the first 30 principle components as input and set the resolution to 0.6 and 0.8, respectively. The default setting was used for the number of neighbors (k=20).

### Cell-type annotation

For the annotation of the cell types (per cluster), the annotations generated with the ‘FindTransferAnchors’ and ‘MapQuery’ functions described above were used. In particular, the cell-type labels queried from the dataset from Baßler et al. (14). were used for annotation. In addition, we validated these cell-type annotations using marker genes. Marker genes per cluster were defined as the most significant DE genes between identified clusters using a Wilcoxon rank sum test for differential gene expression implemented in Seurat. Visualization of the obtained marker genes was performed using Seurat functions such as a dot plot representation of cell type/cluster-specific marker gene expression. A more global overview of the expression profiles was obtained by calculating the mean expression values of marker genes per cluster, followed by scaling and centering of these values and representing them in a heatmap graph using the R package ‘pheatmap’, in which the genes were clustered according to the ‘ward.D’ agglomeration method.

### Sub-clustering of cell-types

For a detailed characterization of the cells in the dataset, the cells of the identified major cell types were isolated, and scaling, dimensionality reduction using PCA, and data integration were repeated as described above. UMAP was then calculated, followed by subclustering according to the strategy described above (with different resolution parameters depending on the cell type studied). To annotate the subclusters, we used marker genes (as described above) in combination with *a priori* knowledge from the public domain.

### Differential expression analysis

The identification of differentially expressed (DE) genes between conditions was performed at the level of major cell types and sub-clusters. We used two strategies for identifying DE genes to account for potential donor effects. (і) For each cell-type, mini-bulks were generated per patient by summing the reads. Next, we loaded the minibulks into ‘DESeq2’ and used its pipeline to identify DE genes. (ii) We denoised the scRNA-seq dataset using the imputation method of ‘SAVER’. Next, we used the denoised dataset as input to the ‘IDEAS’ package. To run the ‘IDEAS’ functions to identify DE genes, we set the ‘fit_method’ argument of ‘ideas_dist’ to “saver_direct” and left the other settings unaltered.

Remark: For the blood dataset, we excluded patient 25 because the sample was contaminated with BALF cells.

### Gene ontology enrichment analysis

Gene ontology (GO) enrichment analysis was performed based on the DE genes between conditions using the ‘clusterProfiler’ package. As background, we used all expressed genes in the dataset.

### Data visualization

The Seurat, pheatmap and ggplot2 packages were used to generate the figures.

### Statistical analysis

If not otherwise stated, statistical analyses were conducted in relation to the total sample size *n*. For the two-condition comparison, the Wilcoxon rank-sum test was used, and for the multi-condition comparison, the Kruskal–Wallis test followed by the Steel–Dwass test was performed. Fisher’s exact test was performed for the categorical variables. Statistical significance was set at p < 0.05.

## Results

### Patient disposition and characteristics

Five patients with SS, eight patients with DM, six patients with RA, six patients with SSc, four patients with AAV, and 10 patients with IIP (as a control) who had newly developed interstitial pneumonia were included in this study (**Table 1**, **Supplementary Table 1**). Blood tests and bronchoscopy were performed before starting treatment for interstitial pneumonia. All patients, except one with RA, were received immunosuppressive therapy following sample collection in this study as needed. Among the patients with DM, one was anti-melanoma differentiation-associated (MDA) 5 protein antibody-positive, while the others had anti-synthetase syndrome. All patients with AAV were positive for myeloperoxidase (MPO)-ANCA. Pulmonary function tests were performed before treatment, and there appeared no significant differences.

### Differences in immune cell proportion in the blood of patients with CTD-ILD

Freshly collected peripheral blood and BALF samples were subjected to single-cell RNA sequencing using the Seq-Well platform. We then compared the distribution of immune cells and differential gene expression profiles.

**Figure 1:**
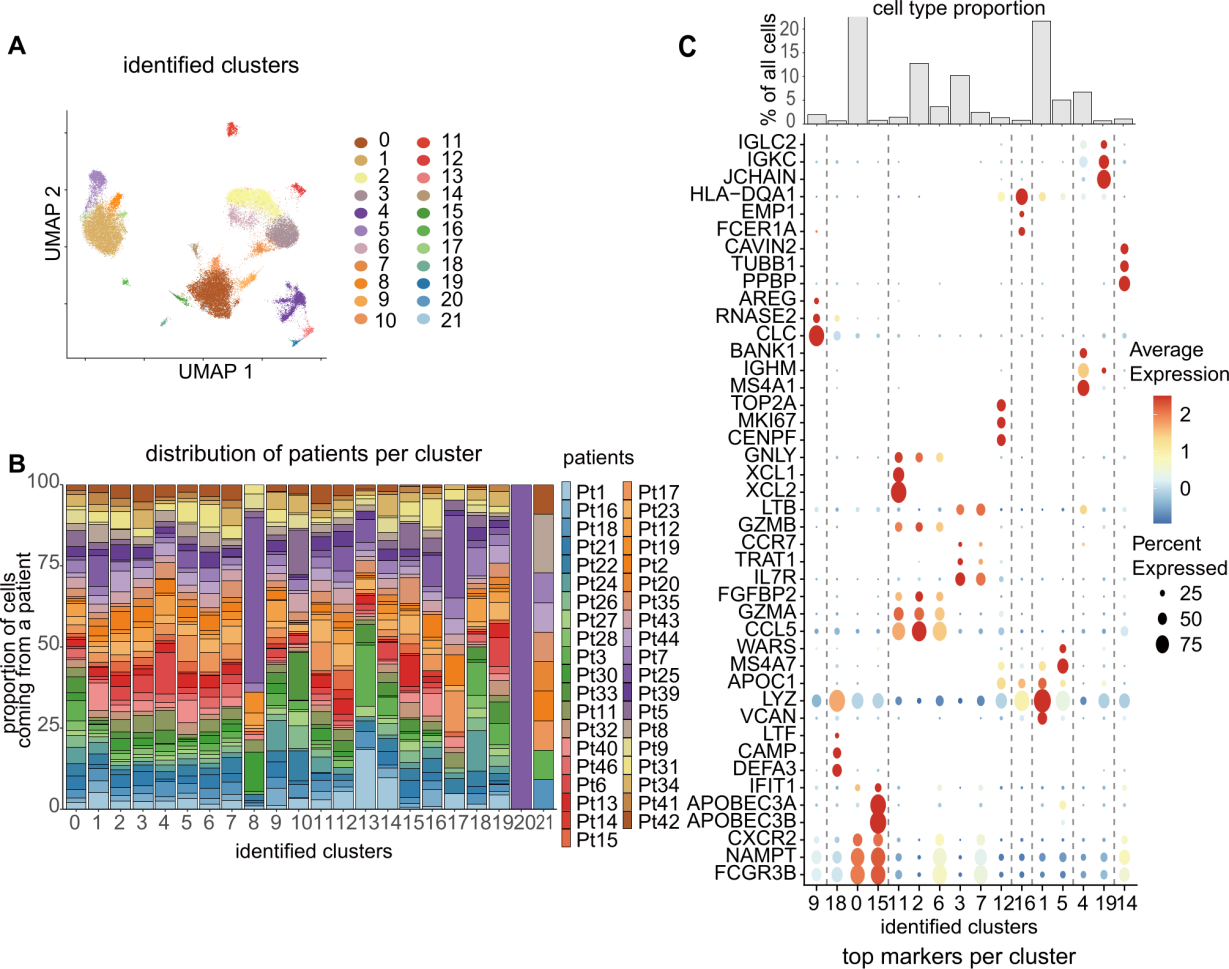
**(A)** UMAP representation of the integrated blood data. The colors and numbers correspond to the identified main clusters. Twenty-two clusters were visualized. **(B)** Distribution of patients per cluster. The potential donor effect was evaluated and clusters that only included certain patients were excluded. **(C)** Dot plots show the top marker genes per cluster and bar charts represent the relative cell proportions in each cluster. Cells abundant in each cluster were identified based on the marker genes.

**Figure 2:**
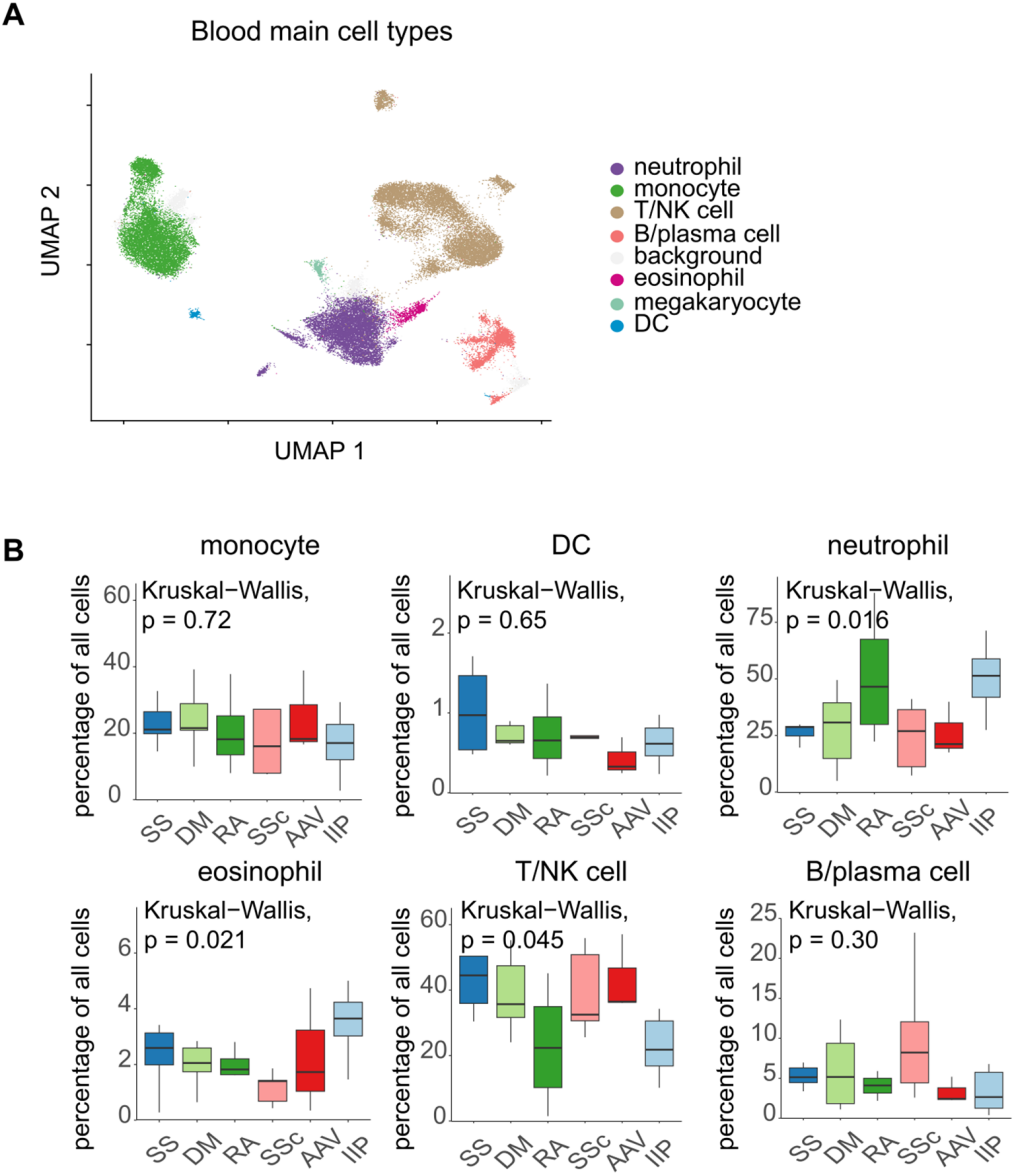
**(A)** Cell type annotation of the integrated blood data according to the step annotation approach. **(B)** Comparison of the proportion of immune cells in the blood among patients with various diseases. The Kruskal–Wallis test followed by the Steel–Dwass test was performed for multi-condition comparison. Statistical significance set at p < 0.05. The percentage of eosinophils was significantly different between systemic sclerosis-associated interstitial lung disease (ILD) and idiopathic interstitial pneumonia-ILD (p = 0.040). The percentage of neutrophils and T/NK cells exhibited no substantial variance in the *post hoc* analysis. SS, Sjögren’s syndrome; DM, dermatomyositis; RA, rheumatoid arthritis; SSc, systemic sclerosis; AAV, ANCA-associated vasculitis; IIP, idiopathic interstitial pneumonia.

Starting with the blood cells, we used a clustering approach and visualized the data in 22 clusters using UMAP (**Figure 1A**). The potential donor effect was assessed and clusters were selected for further analysis, excluding those formed by only a few patients (**Figure 1B**). After characterizing the cells within each cluster using marker genes (**Figure 1C**), we identified the major cell types found in the blood based on previously reported annotation methods (14). After identifying the cell types (**Figure 2A**), we compared the cell populations among patients with different CTD-ILDs (**Figure 2B**). For instance, the percentage of neutrophils tended to increase in patients with RA-associated ILD (RA-ILD), while that of B/plasma cells tended to increase in patients with SSc-associated ILD (SSc-ILD). T/NK cells were further subclassified to discriminate between T cells and NK cells and the differences between diseases were compared (**Supplementary Figure 2**). We also performed multi-color flow cytometry (MCFC) and found similar, although not significant, trends in the percentage of neutrophils in RA-ILD or B cells in SSc-ILD (**Supplementary Figures 1**, **3**).

### Differences in immune cell proportion in the BALF of patients with CTD-ILD

**Figure 3:**
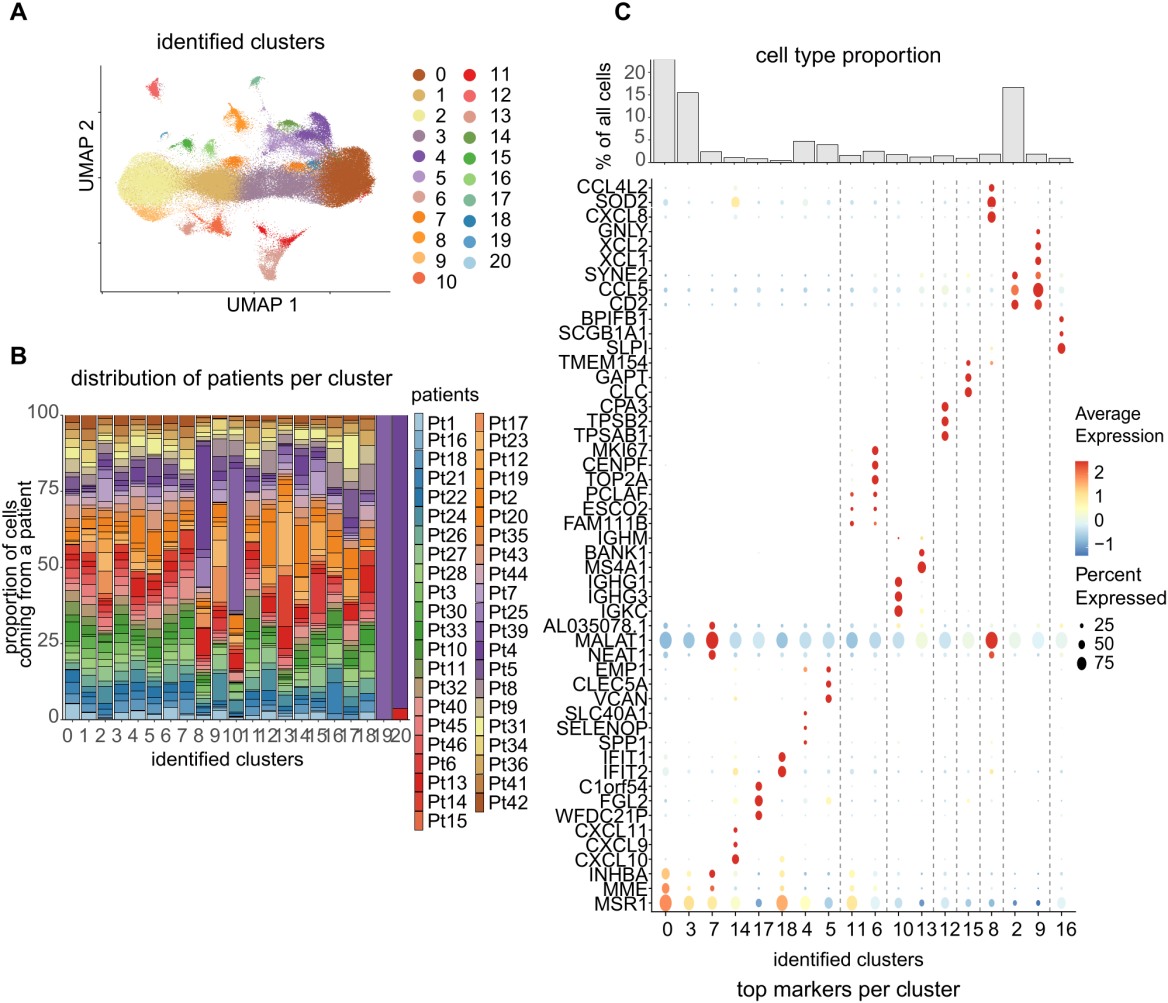
**(A)** UMAP representation of the integrated bronchoalveolar lavage fluid (BALF) data. **(B)** Distribution of patients per cluster. The potential donor effect was evaluated and clusters that only included certain patients were excluded. **(C)** Dot plots show the top marker genes per cluster and bar charts represent the relative cell proportions in each cluster.

**Figure 4:**
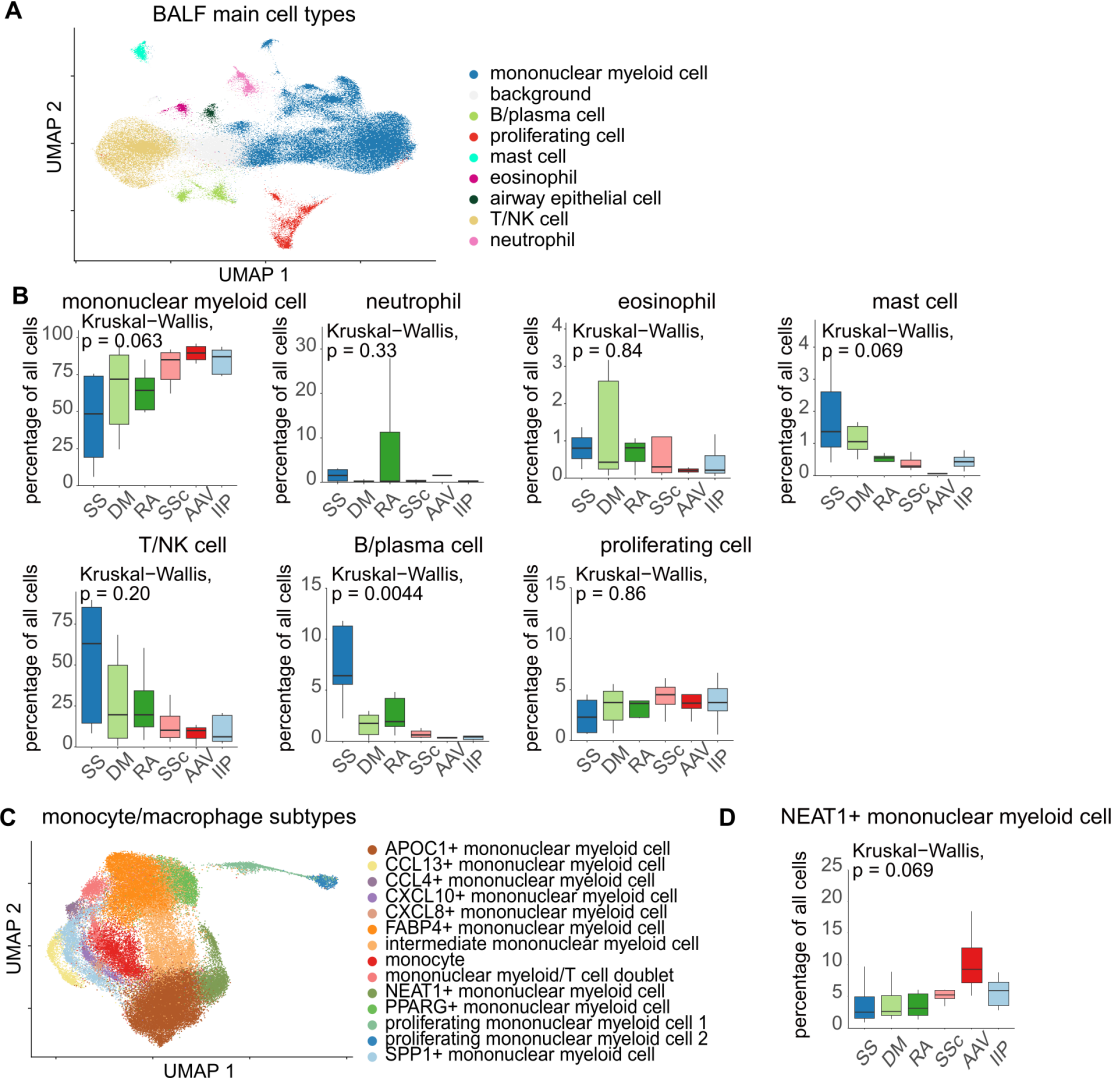
**(A)** Cell type annotation of the integrated BALF data according to the step annotation approach. **(B)** Comparison of the proportion of immune cells in the BALF among patients with various diseases. **(C)** Phenotypic classification of mononucleolar myeloid cells based on the expression of major genes. **(D)** Comparison of the proportion of each subtype of mononuclear myeloid cells. The percentage of B/plasma cells exhibited no substantial variance in the *post hoc* analysis. The Kruskal–Wallis test followed by the Steel–Dwass test was performed for multi-condition comparisons. Statistical significance was set at p < 0.05. SS, Sjögren’s syndrome; DM, dermatomyositis; RA, rheumatoid arthritis; SSc, systemic sclerosis; AAV, ANCA-associated vasculitis; IIP, idiopathic interstitial pneumonia; mononuclear myeloid cell, the fraction including monocytes, alveolar macrophages, and dendritic cells.

A clustering approach was performed for analyzing BALF cells, similar to that done using blood cells. Herein, we visualized the data from 21 clusters using UMAP (**Figure 3A**). The potential donor effect was assessed, and again clusters were chosen by excluding those that solely included a few patients (**Figure 3B**). By identifying the predominant cells in each cluster using marker genes (**Figure 3C**), we ascertained the major cell types present in the BALF based on previously reported annotation methods (14). Mononuclear myeloid cells, including monocytes, alveolar macrophages (AMs), and dendritic cells, were the most abundant immune cells in the BALF, which showed high heterogeneity (**Figure 4A**). We compared the cell populations in the BALF of patients with CTD-ILD (**Figure 4B**). Compared with patients with other CTD-ILDs, the percentage of B/plasma cells remarkably increased in patients with SS-associated ILD (SS-ILD). Furthermore, patients with SS-ILD had more mast cells. The proportion of neutrophils tended to increase in patients with RA-ILD, while that of mononuclear myelocytes tended to increase in patients with AAV-associated ILD (AAV-ILD) or SSc-ILD. We examined the cell proportions using MCFC and found similar trends (**Supplementary Figures 1**, **4**).

We found that mononuclear myeloid cells constitute the majority of immune cells in the BALF, so we further hypothesized that the state of predominant AMs differs depending on the underlying disease (14, 28). Therefore, we subclassified mononuclear myeloid cells based on a previous report (14) (**Figure 4C**, **Supplementary Figures 5A–C, E**), and investigated the differences in the subtypes of mononuclear myeloid cells among CTD-ILDs (**Figure 4D**, **Supplementary Figure 5F**). The proportion of mononuclear myeloid cells expressing nuclear enriched abundant transcript 1 (*NEAT1*), a long noncoding RNA known to promote macrophage inflammasome activation and enhance interleukin 1β (IL-1β) maturation (29), was increased in patients with AAV-ILD. Some clusters of mononuclear myeloid cells also showed signatures characteristic of tissue-resident macrophages based on the previous reports (30, 31) (**Supplementary Figure 5D**). In order to separate T cells from NK cells, T/NK cells were also subclassified (**Supplementary Figure 6**), and several T cell fractions appeared to be increased in SS-ILD.

### Gene ontology enrichment analysis in patients with SS-ILD

There are few reports of ILD in patients with SS, and there is no established treatment for SS-ILD (32). Therefore, we first sought to clarify the pathogenesis of SS-ILD by focusing on the functional changes of immune cells in the BALF and blood. We identified a large number of differentially expressed (DE) genes in patients with SS-ILD using the package IDEAS (33) for each immune cell type compared to other diseases (**Supplementary Tables 5**, **6**), then applied gene ontology (GO) enrichment analysis to estimate their functions and relationships. GO enrichment analysis was performed on monocytes-macrophages (mononuclear myeloid cells), neutrophils, T/NK cells, and B/plasma cells in the BALF and monocytes, neutrophils, T/NK cells, and B/plasma cells in the blood of patients with SS-ILD. Notable significant findings are presented below.

**Figure 5:**
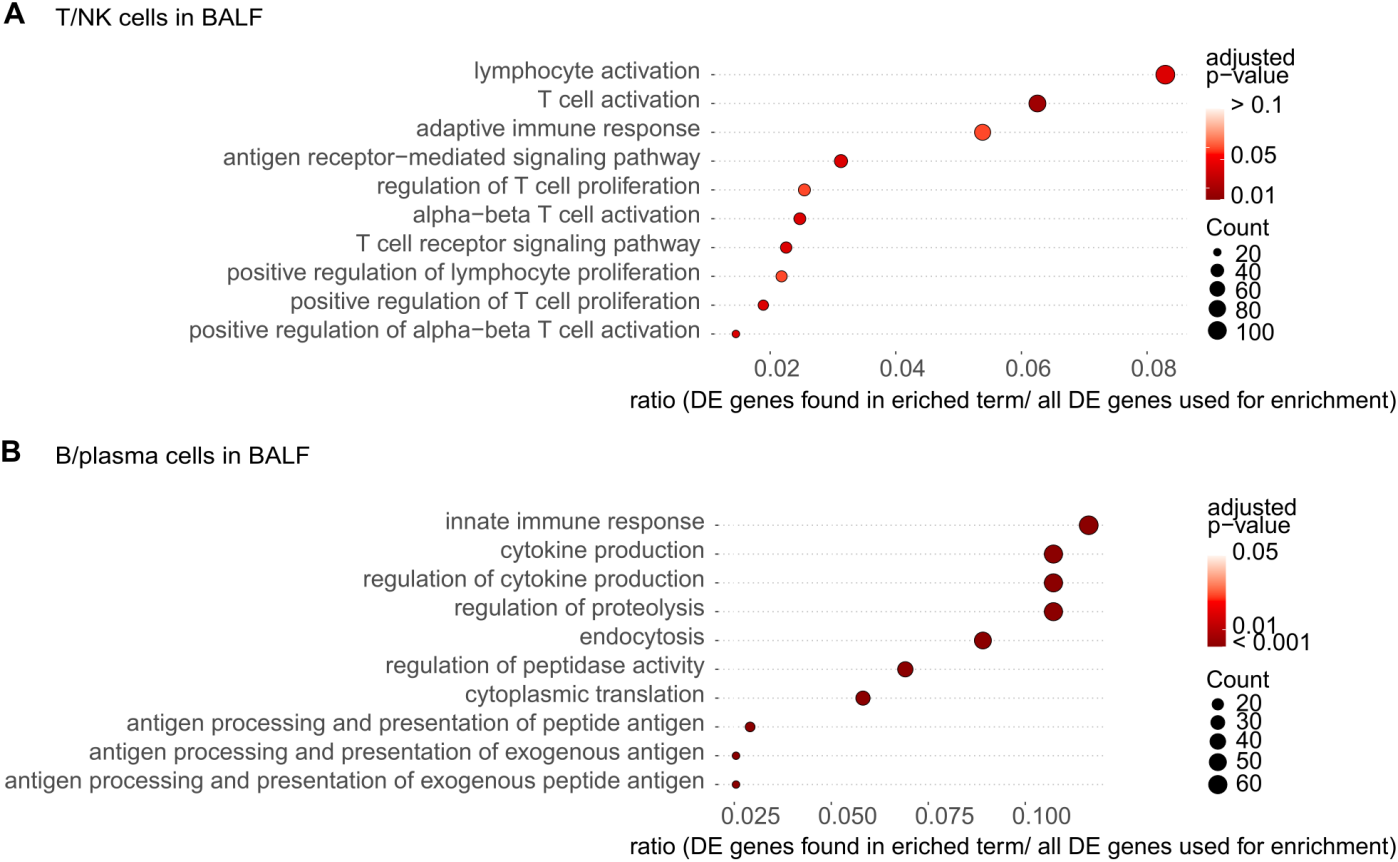
Gene ontology (GO) enrichment analysis of differentially expressed (DE) genes in the bronchoalveolar lavage fluid (BALF) from patients with Sjögren’s syndrome (SS)-associated interstitial lung disease (ILD). The most significantly enriched pathways in each immune cell were visualized using dot plots. **(A)** T/NK cells in the BALF. **(B)** B/plasma cells in the BALF. The p-value cutoff for genes was set at 0.05 for T/NK cells and B/plasma cells in the BALF. Dot plots show the enriched terms. The size of the dot corresponds to the gene count enriched in the pathway, and the color of the dot indicates the pathway enrichment significance.

As mentioned above, the proportion of B/plasma cells in the BALF tended to increase in patients with SS-ILD compared to those with other diseases. In the B/plasma cells from the BALF of patients with SS-ILD, terms associated with innate immune responses and the acquired immune system related to antigen presentation were enriched (**Figure 5B**). The guanylate-binding protein (GBP) family of the interferon (IFN)-inducible GTPases is involved in the innate immune response (**Supplementary Figure 7B**). Among the GBP members, *GBP4* and *GBP5* were identified as DE genes with significant differences (p < 0.01) (**Supplementary Table 5**). In T/NK cells from the BALF of patients with SS-ILD, dipeptidyl peptidase-4 involved in T cell activation was also enriched (**Figure 5A**, **Supplementary Figure 7A**).

**Figure 6:**
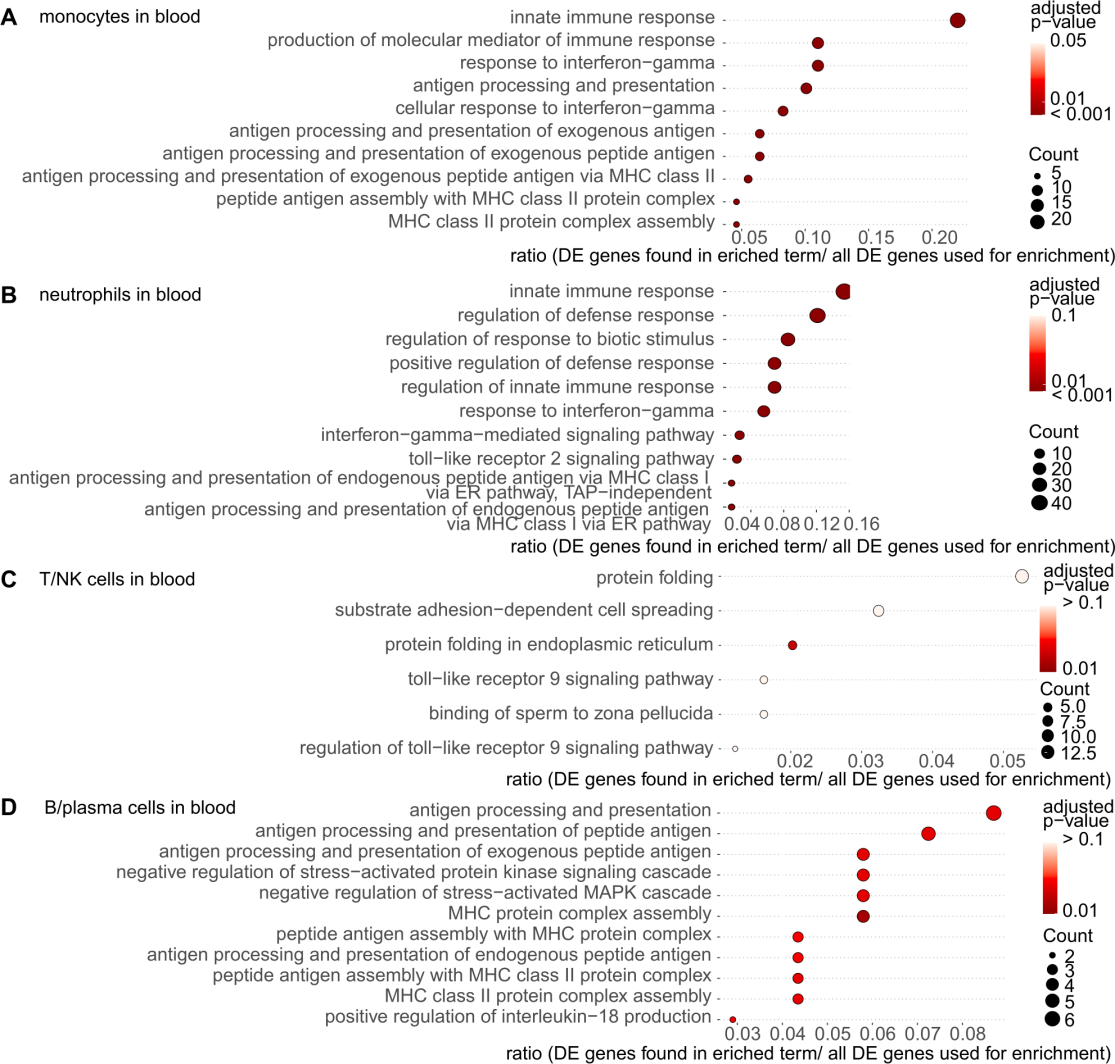
Gene ontology (GO) enrichment analysis of differentially expressed **(DE)** genes in the blood cells from patients with Sjögren’s syndrome (SS)-associated interstitial lung disease (ILD). The most significantly enriched pathways in each immune cell were visualized using dot plots. **(A)** Monocytes in the blood. **(B)** Neutrophils in the blood. **(C)** T/NK cells in the blood. **(D)** B/plasma cells in the blood. The p-value cutoff for genes was set at 0.05 for T/NK cells and B/plasma cells in the blood and 0.01 for monocytes and neutrophils in the blood. Dot plots show the enriched terms. The size of the dot corresponds to the gene count enriched in the pathway, and the color of the dot indicates the pathway enrichment significance.

In blood monocytes and neutrophils, terms associated with the innate immune response, response to IFNγ, and antigen processing and presentation were enriched (**Figures 6A, B**); genes of the GBP family and the JAK-STAT pathway were included (**Supplementary Figures 7C, D**). In B/plasma cells in the blood of patients with SS-ILD, the enriched terms were mainly related to antigen presentation and proteasome 20S subunit beta 8 (*PSMB8*), a gene located in the class II region of the major histocompatibility complex and induced by IFNγ, suggesting the influence of IFNγ on various cells (**Figure 6D**, **Supplementary Figure 7F**). The terms related to protein folding and toll-like receptor 9 signaling pathway were enriched in T/NK cells in the blood (**Figure 6C**, **Supplementary Figure 7E**). These findings suggest that the innate and acquired immune systems play important roles in lung and blood pathogenesis in patients with SS-ILD. Furthermore, the involvement of IFNγ signaling was suggested in a wide range of cells.

### Gene ontology enrichment analysis in patients with DM-associated ILD

Next, we examined the pathogenesis of DM-associated ILD (DM-ILD) because ILD is an important prognostic determinant for patients with DM (2). Using IDEAS (33) for each immune cell type, we identified a large number of DE genes in patients with DM-ILD compared to those with other diseases (**Supplementary Tables 5**, **6**). We then applied GO enrichment analysis to determine their functions and relationships as described above.

**Figure 7:**
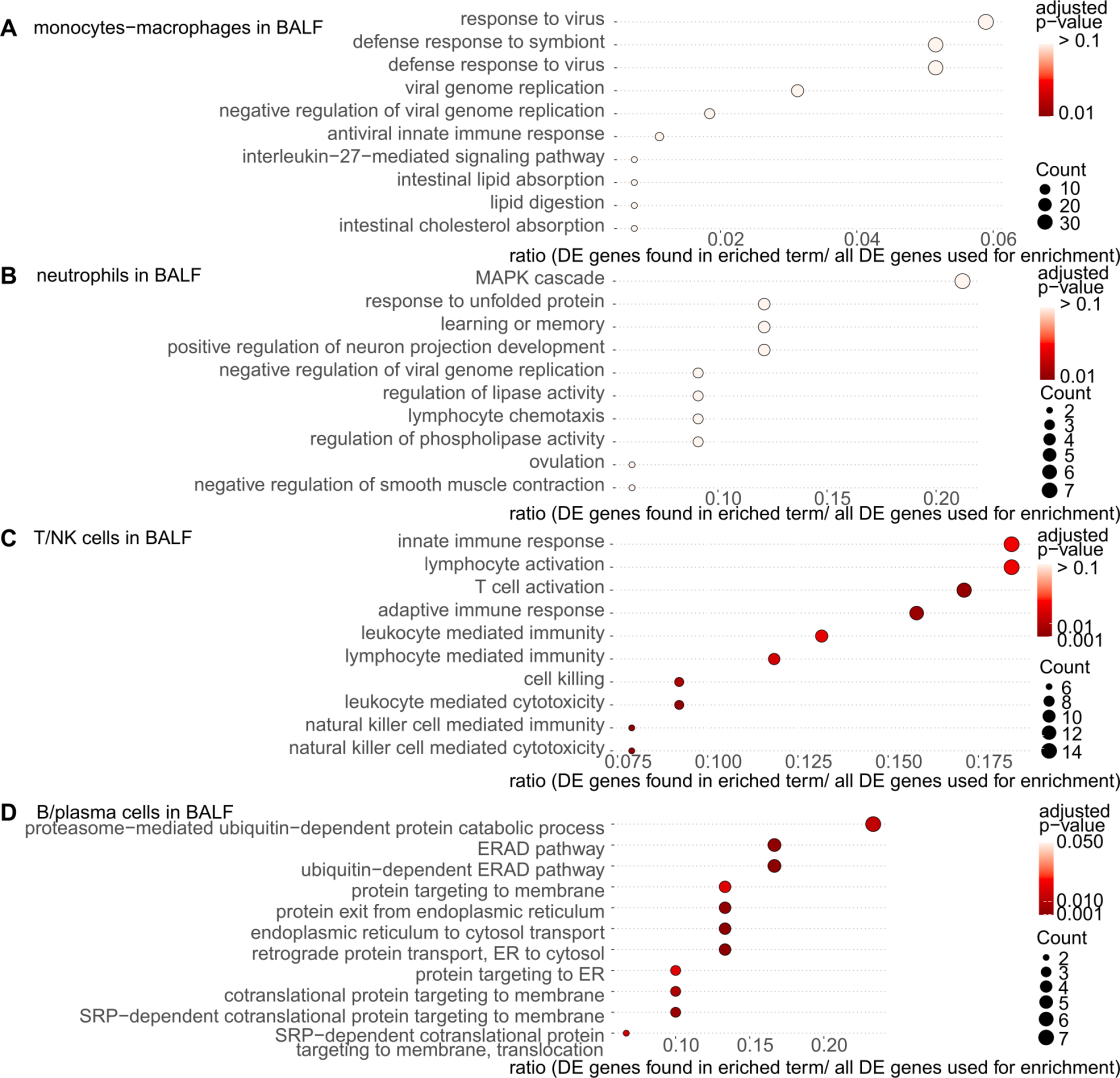
Gene ontology (GO) enrichment analysis of differentially expressed (DE) genes in the bronchoalveolar lavage fluid (BALF) cells from patients with dermatomyositis (DM)-associated interstitial lung disease (ILD). The most significantly enriched pathways in each immune cell were visualized using dot plots. **(A)** Monocyte-macrophages in the BALF. **(B)** Neutrophils in the BALF. **(C)** T/NK cells in the BALF. **(D)** B/plasma cells in the BALF. The p-value cutoff for genes was set at 0.05 for monocyte-macrophages and neutrophils in the BALF and 0.01 for T/NK cells and B/plasma cells in the BALF. ER, endoplasmic-reticulum; ERAD, endoplasmic-reticulum-associated protein degradation.

In monocytes-macrophages in the BALF, terms associated with virus and symbiont were enriched (**Figure 7A**), including IFN-related genes such as IFN-induced protein with tetratricopeptide repeats 1 (*IFIT1*) and C-X-C motif chemokine ligand 10 (*CXCL10*) (**Supplementary Figure 8A**). In neutrophils in the BALF, the term related to the viral genome was also enriched. In addition, the term associated with lymphocyte chemotaxis was enriched, also including CXCL10. (**Figure 7B**, **Supplementary Figure 8B**). In T/NK cells in the BALF, the enriched terms were mainly related to the innate immune response, lymphocyte activation, and T cell activation (**Figure 7C**, **Supplementary Figure 8C**). In B/plasma cells in the BALF, terms related to catabolic process and endoplasmic reticulum were enriched (**Figure 7D**).

**Figure 8:**
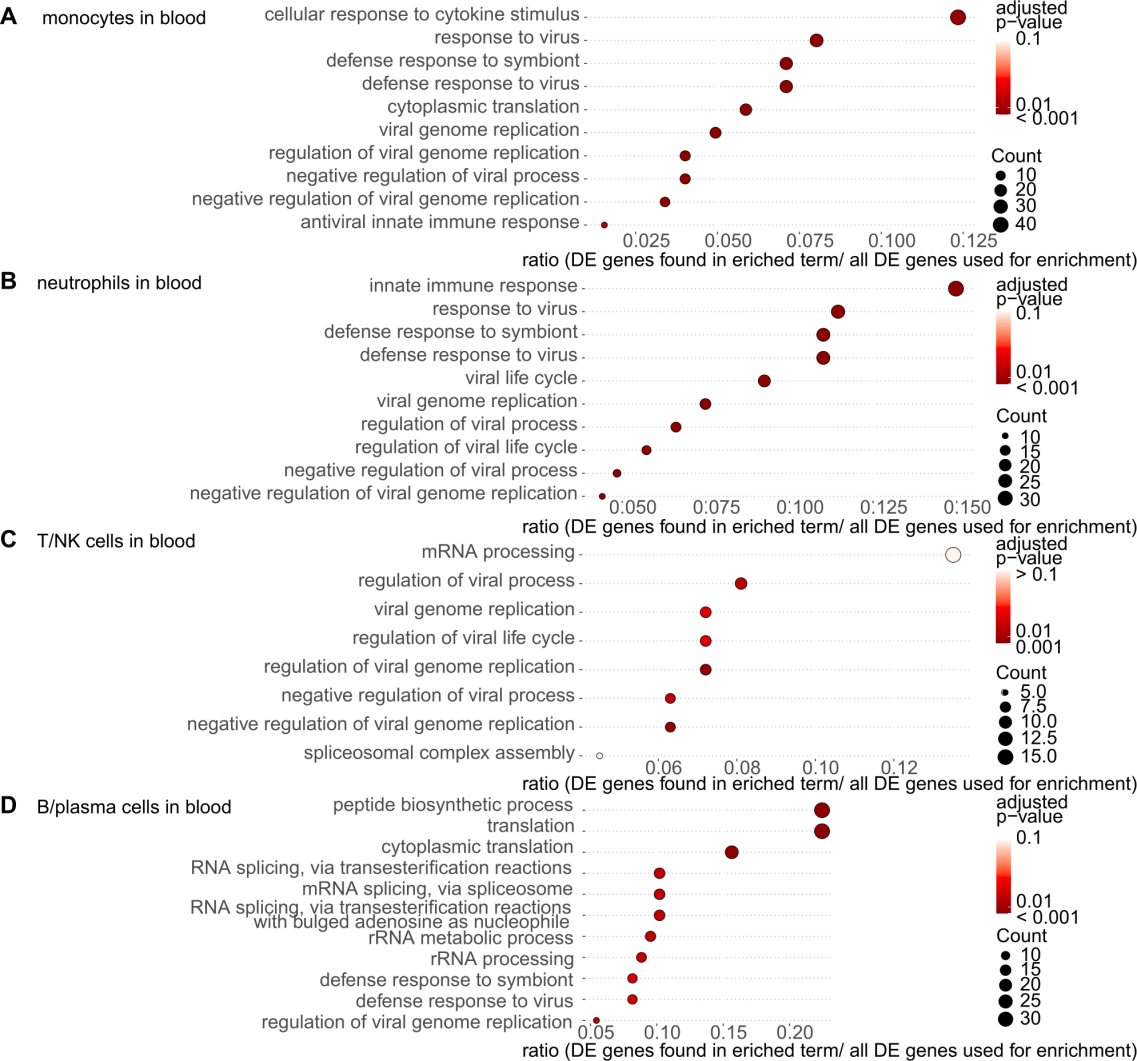
Gene ontology (GO) enrichment analysis of differentially expressed (DE) genes in the blood cells from patients with dermatomyositis (DM)-associated interstitial lung disease (ILD). The most significantly enriched pathways in each immune cell were visualized using dot plots. **(A)** Monocytes in the blood. **(B)** Neutrophils in the blood. **(C)** T/NK cells in the blood. **(D)** B/plasma cells in the blood. The p-value cutoff for genes was set at 0.05 for monocytes, neutrophils, and B/plasma cells in the blood and 0.01 for T/NK cells in the blood.

In monocytes, neutrophils, and B/plasma cells in the blood, terms associated with response to virus and symbiont were enriched and in T/NK cells in the blood, terms related to virus also enriched (**Figures 8A–D**); the expression of the myxovirus resistance 1 (*MX1*) gene, which encodes an IFN-induced protein with antiviral activity, was common in these four types of blood cells (**Supplementary Figures 8E–H**). These findings suggest that IFN- and virus infection-related pathways were upregulated in a wide range of cells in both lung and blood pathogenesis in patients with DM-ILD.

### Gene ontology enrichment analysis in patients with RA-ILD, SSc-ILD, and AAV-ILD

ILD is also a significant complication for patients with RA and is associated with increased mortality (34). We also identified DE genes in patients with RA-ILD using IDEAS and performed GO enrichment analysis. The blood and BALF cell fractions in RA-ILD showed a characteristic increase in the proportion of neutrophils (**Figures 2B**, **4B**). In neutrophils in the BALF, terms related to cell adhesion, intracellular receptor and pattern recognition receptor signaling pathway, and negative regulation of nuclear factor-kappa B transcription factor activity were enhanced (**Supplementary Figure 9B**). In monocytes-macrophages in the BALF, terms associated with inflammation and bacterium were enriched (**Supplementary Figure 9A**). In neutrophils and monocyte-macrophages in the BALF, genes related to tumor necrosis factor and nuclear factor-kappa B were included (**Supplementary Figures 9C, D**). In B/plasma cells in the BALF, terms associated with B cell activation, immunoglobulin mediated immune response, and complement activation were enriched (**Supplementary Figure 9E**).

The progression of ILD is variable in patients with SSc-ILD, so it is important to identify patients with early pulmonary function loss (35). In blood B/plasma cells, terms associated with innate immunity, cytokine production, and translation were enriched (**Supplementary Figure 10A**). Terms associated with cytokine production were also enriched in neutrophils in the BALF (**Supplementary Figures 10C, D**) and the terms related to negative regulation of lymphocyte activation and toll-like receptor signaling pathway were enriched in neutrophils in the blood (**Supplementary Figures 10E, F**). The terms associated with endoplasmic reticulum tubular network and protein folding were enriched in monocytes-macrophages in the BALF (**Supplementary Figures 10G, H**). In contrast, the terms related to mRNA were enriched in monocytes in the blood (**Supplementary Figures 10I, J**).

In patients with AAV-ILD, DESeq2 showed significant DE genes. *RETN*, the gene encoding resistin, was upregulated in monocyte-macrophages and neutrophils in the BALF of patients with AAV-ILD (**Supplementary Table 5**).

### Differences in cytokine/chemokine levels and complement activation

**Figure 9:**
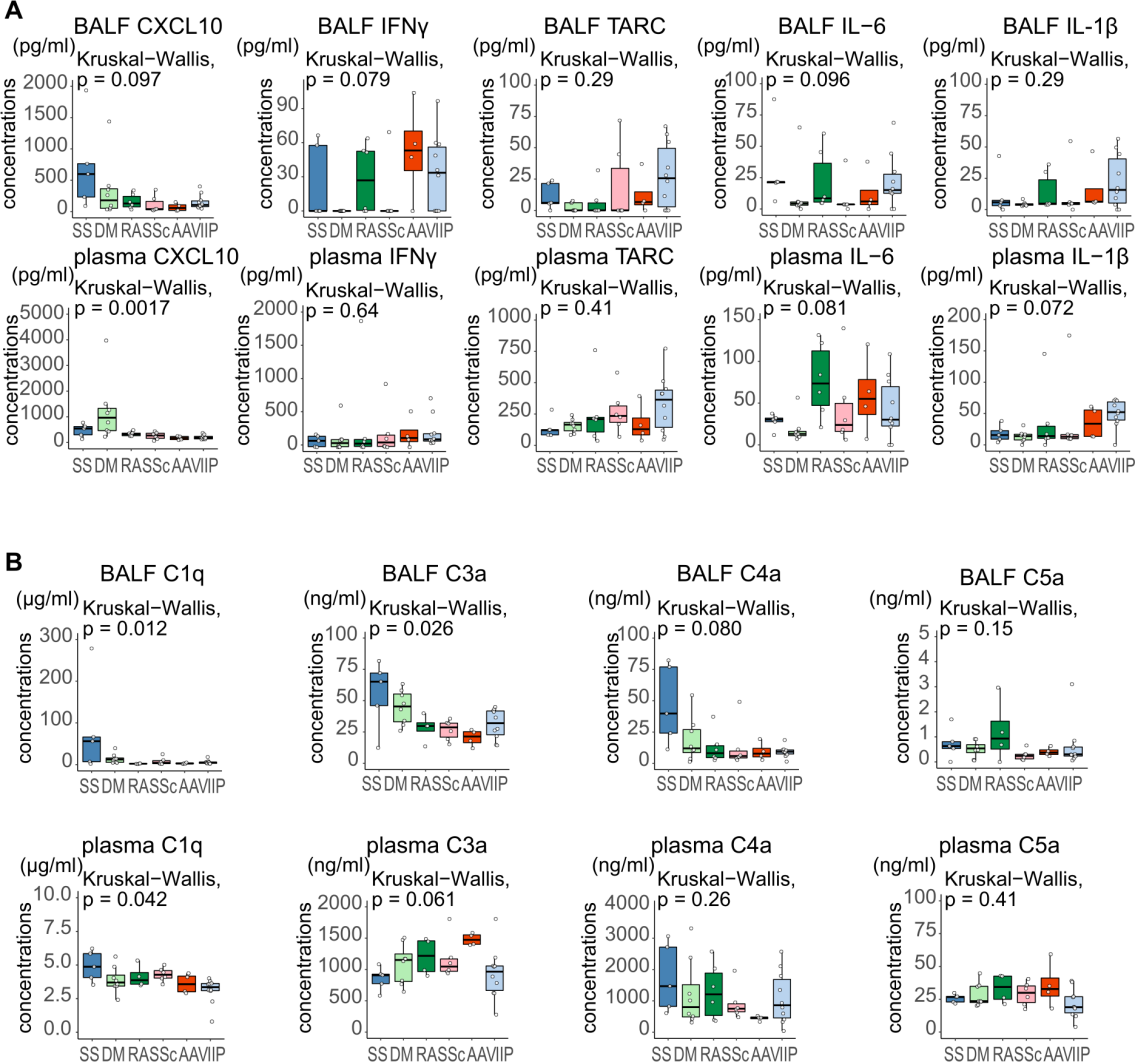
**(A)** Comparison of cytokine and chemokine levels in the bronchoalveolar lavage fluid (BALF) supernatants and plasma among patients with various diseases. **(B)** Comparison of complement levels in the BALF supernatants and plasma among patients with various diseases. CXCL10 levels in the plasma were significantly elevated in patients with dermatomyositis (DM)-associated interstitial lung disease (ILD) compared to patients with idiopathic interstitial pneumonia (IIP) (p = 0.013). Complement levels were significantly different in the plasma and BALF of patients with different diseases. The Kruskal–Wallis test followed by the Steel–Dwass test was performed for multi-condition comparison. Statistical significance was set at p < 0.05. SS, Sjögren’s syndrome; DM, dermatomyositis; RA, rheumatoid arthritis; SSc, systemic sclerosis; AAV, ANCA-associated vasculitis; IIP, idiopathic interstitial pneumonia; CXCL10, C-X-C motif chemokine ligand 10; IFNγ, interferon-gamma; TARC, Thymus and Activation-Regulated Chemokine; IL, interleukin.

Our RNA sequencing data indicate a distinct distribution of cellular fractions within each CTD-ILD. Moreover, it suggests that diverse genetic pathways were enriched, leading to alterations in immune cell functions. We hypothesized that there are differences in cytokine/chemokine profiles that reflect the pathogenesis of CTD-ILDs. We then measured cytokine/chemokine levels in BALF supernatants and plasma using ELISA (**Figure 9A**, **Supplementary Figure 11**). Levels of CXCL10, which are produced in response to IFNγ, in the plasma were significantly elevated in patients with DM-ILD, while its levels in the BALF tended to be elevated in patients with SS-ILD. In addition, interleukin 6 (IL-6) levels in the plasma tended to be elevated in patients with RA-ILD. Moreover, thymus and activation-regulated chemokine (TARC) and interleukin 1 β (IL-1β) levels in the plasma tended to be increased in patients with SSc-ILD and AAV-ILD, respectively. In addition, the exacerbation of pulmonary fibrosis by C1q has been reported before (36), suggesting the importance of C1q in the pathogenesis of CTD-ILD. Therefore, we measured C1q levels in the BALF supernatants and plasma using ELISA. We also measured C3a, C4a, and C5a levels to determine the status of complement pathway activation in CTD-ILDs from complement values, such as C3 and C4, which are routinely measurable in clinical practice. Differences in complement levels are shown in **Figure 9B**. In patients with SS-ILD, C1q, C3a, and C4a levels were elevated in the BALF. Moreover, C5a levels in the BALF of patients with RA-ILD tended to be elevated.

We found that each disease had a distinct profile of cytokines/chemokines and complements. Using our sequencing data, we compared the relative mRNA expression levels of these proteins across different diseases and cell types (**Supplementary Figure 12**). mRNA levels for IL-6 and IL-1β were not elevated in RA-ILD or AAV-ILD, respectively, unlike their protein levels. In SS-ILD, *CXCL-10* was upregulated in blood monocytes, and Interferon Gamma (*IFNG)* was upregulated in T/NK cells and neutrophils in BALF, suggesting a role of IFNγ in the pathogenesis of SS-ILD. In RA-ILD monocytes, increased Complement C1q B Chain and C Chain (*C1QB* and *C1QC*) expression suggested complement activation.

## Discussion

In this study, we analyzed the BALF and blood from patients with newly developed CTD-ILD using single-cell RNA sequencing and investigated the cellular distribution status and gene expression patterns of the immune cells for each CTD-ILD. To the best of our knowledge, this is the first study to comprehensively analyze BALF and blood samples from patients with CTD-ILD at a single-cell level.

First, the proportion of B/plasma cells in the BALF was remarkably increased in patients with SS-ILD. A previous study reported that lymphocyte proliferation in the BALF of patients with SS-ILD suggests interstitial pneumonia activity (37); however, detailed subset reports are scarce. We also found an increased number of mast cells in the BALF of patients with SS-ILD. Mast cells are a source of TGFβ (38), and TGFβ-positive mast cells are involved in lung fibrosis (39). Thus, mast cells may be involved in fibrosis in SS-ILD. Furthermore, neutrophils in the BALF of patients with RA-ILD tended to increase, consistent with a previous report (40). Moreover, higher percentages of mononuclear myeloid cells in the BALF were observed in patients with AAV-ILD and SSc-ILD, and a more detailed analysis revealed differences in the phenotypes of these mononuclear myeloid cells, such as an increase in the percentage of mononuclear myelocytes expressing *NEAT1* in patients with AAV-ILD. Upregulated *NEAT1* expression was involved in the development of fibrosis in various organs, including pulmonary fibrosis (41). Therefore, patients with each CTD-ILD had characteristics of the cellular fraction in the BALF, which are primarily involved in local lung pathology and may be potential therapeutic targets.

Next, we performed GO enrichment analysis of the DE genes in patients with each CTD-ILD compared to those of other diseases. We also examined cytokine, chemokine, and complement levels as possible indicators of each CTD-ILD for clinical applications. Enrichment analysis revealed the importance of innate immunity and acquired immunity in the pathogenesis of SS-ILD, which is consistent with recent reports (42). We also found those pathways were activated not only in blood monocytes and neutrophils but also in the B/plasma cells in the BALF. Moreover, in our study, the IFNγ signaling molecule type 2 IFN was enriched in monocytes and neutrophils in the blood. Although type 1 IFN activation has been reported in patients with SS (43, 44), populations with predominant type 2 IFNs have also been reported (45). Type 2 IFNs have been suggested to play important roles in the pathogenesis of active ILD. We also found a remarkable elevation of C1q levels in the BALF supernatants. C1q is required for Th1-type responses (46) and also contributes to removing dead cells and the polarization of macrophages (47). This may also support the importance of both acquired and innate immunity. In short, pathways related to innate immunity, acquired immunity, and IFN signaling were similarly enriched in the immune cells in both the blood and BALF of patients with SS-ILD. Systemic and local lung immune cell functions are similarly altered in SS-ILD, suggesting the involvement of common pathways in systemic immune abnormalities and pulmonary complications.

In DM-ILD, enrichment analysis unveiled that the pathways involved in virus response operated across various cellular lineages. Analysis of circulating monocytes in patients with anti-MDA5-associated ILD reported that an antiviral inflammatory network might be involved in the cytokine storm (48). We observed both immune cells in the BALF and blood exhibited a network related to viral response, signifying the commonality of this pathway across diverse cell types and its pivotal role in pathogenesis. Additionally, elevated expressions of type 1 IFN-induced genes, such as *MX1* and *IFIT1* were reported in the peripheral blood mononuclear cells of patients with active DM (49). Our investigation has also revealed the elevated expression of IFN-related genes in a wide range of immune cells in the BALF and blood. Furthermore, the activity of DM was correlated with elevated serum CXCL10 levels (50), and we also found that CXCL10 was increased in the plasma of patients with DM-ILD. In conclusion, pathways related to antiviral response and IFN were closely associated with the pathogenesis of DM-ILD, both in local lung pathogenesis and systemic immune abnormality.

In RA-ILD, enrichment analysis showed the upregulation of inflammation-associated genes in the neutrophils and monocyte-macrophages in the BALF. Inflammatory cells such as neutrophils are essentially absent in the alveoli during homeostasis because neutrophil migration is prevented by patrols of AMs, such as by processing bacteria beforehand (51). The infiltration of neutrophils into the BALF of patients with RA-ILD may indicate a breakdown of local lung homeostasis. Additionally, the enrichment analysis of B/plasma cells in the BALF showed an upregulation of the terms related to B cell activation and humoral immunity. In RA-ILD, it has been reported that tertiary lymphoid structures develop at locally affected sites, surrounded by plasma cells that produce high-affinity antibodies and a neutrophilic infiltrate in contact with these plasma cells (52). In conclusion, the alterations observed within the BALF may imply the significance of local pulmonary pathogenesis in the context of RA-ILD.

In SSc-ILD, the enrichment analysis of blood B/plasma cells showed the involvement of terms associated with innate immunity and cytokine production, and B cell depletion therapy, rituximab, that has been reported to prevent the worsening lung fibrosis (53) may have an effect on this aspect. Furthermore, in monocytes-macrophages in BALF, pathways related to the endoplasmic reticulum tubular network were enhanced. The endoplasmic reticulum stress has been reported to be associated with pulmonary fibrosis and to play a role in macrophage polarization to the M2 phenotype, accompanied by the increased production of fibrotic mediators (54). It was also reported that in pulmonary arterial hypertension with limited cutaneous systemic sclerosis, the activation of endoplasmic reticulum stress may contribute to driving inflammation (55). These suggest that endoplasmic reticulum stress may also play some role in SSc-ILD. Moreover, serum TARC levels were reported to be elevated in patients with SSc-ILD (56); this study also indicated a similar tendency.

In AAV-ILD, *RETN*, the gene encoding resistin, was upregulated in the monocyte-macrophages and neutrophils in the BALF. Macrophage-expressed resistin has important roles in inflammation (57) and resistin stimulates neutrophils to pro-inflammatory activation and promotes neutrophil extracellular trap (NET) formation (58). The NET formation is deeply involved in the pathogenesis of AAV (59), suggesting that *RETN* may play an important part in the lung pathogenesis in AAV-ILD. Furthermore, IL-1β in plasma tended to rise in patients with AAV-ILD. IL-1β produced by macrophages prime neutrophils and plays a critical role in the pathogenesis of AAV (59). Elevated blood IL-1β levels may mirror AAV pathogenesis. We also found an increase in alveolar macrophages expressing *NEAT1*, linked to IL-1β, which may imply localized effects of IL-1β in the lungs.

Our study had certain limitations. First, as this was an observational study with a small sample size conducted in a clinical setting, the clinical phenotype, history of smoking, and severity of ILD were not standardized. Further studies with larger populations are required to confirm our generalizations. Second, we only included patients with systemic conditions allowing the collection of BALF, leaving critical cases unexplored. Third, although various changes in gene expression were observed, their interactions and functional changes were not validated; these must be considered in future investigations.

In conclusion, our comprehensive single-cell analysis of the BALF and blood showed characteristic immune cell distributions and functional changes in patients with CTD-ILD. In the immune cells in both the blood and BALF, we found that pathways associated with virus and IFN signaling were enriched in DM-ILD, while those associated with innate immunity, acquired immunity, and IFN signaling were enhanced in SS-ILD. These findings imply an interaction between systemic immune abnormalities and local lung pathogenesis in DM-ILD and SS-ILD. For RA-ILD, the significance of localized pulmonary inflammation was suggested; the lung microenvironment seemed important for RA-ILD pathogenesis. The characteristics of these immune cells may reflect the distinct pathogenesis of each disease. Our findings shed light on understanding the diversity of pathogenesis in CTD-ILDs and would provide new biomarkers useful for these diseases.

## Data Availability

The scRNA-seq data have been deposited in the European Genome-phenome Archive (EGA) database (EGAD00001011334).

## References

[1] Kondoh Y Makino S Ogura T Suda T Tomioka H Amano H. 2020 Guide for the diagnosis and treatment of interstitial lung disease associated with connective tissue disease. Respir Investig. (2021) 59:709–40. doi: 10.1016/j.resinv.2021.04.011 34602377

[2] Jeganathan N Sathananthan M. Connective tissue disease-related interstitial lung disease: prevalence, patterns, predictors, prognosis, and treatment. Lung. (2020) 198:735–59. doi: 10.1007/s00408-020-00383-w 32780179

[3] Wells AU Denton CP. Interstitial lung disease in connective tissue disease–mechanisms and management. Nat Rev Rheumatol. (2014) 10:728–39. doi: 10.1038/nrrheum.2014.149 25266451

[4] Hata K Yanagihara T Matsubara K Kunimura K Suzuki K Tsubouchi K. Mass cytometry identifies characteristic immune cell subsets in bronchoalveolar lavage fluid from interstitial lung diseases. Front Immunol. (2023) 14:1145814. doi: 10.3389/fimmu.2023.1145814 36949950 PMC10027011

[5] Kuwana M Bando M Kawahito Y Sato S Suda T Kondoh Y. Identification and management of connective tissue disease-associated interstitial lung disease: evidence-based Japanese consensus statements. Expert Rev Respir Med. (2023) 17:71–80. doi: 10.1080/17476348.2023.2176303 36786105

[6] Tomassetti S Colby TV Wells AU Poletti V Costabel U Matucci-Cerinic M. Bronchoalveolar lavage and lung biopsy in connective tissue diseases, to do or not to do? Ther Adv Musculoskelet Dis. (2021) 13:1759720X211059605. doi: 10.1177/1759720X211059605 PMC8664307 34900002

[7] Raghu G Remy-Jardin M Richeldi L Thomson CC Inoue Y Johkoh T. Idiopathic pulmonary fibrosis (an update) and progressive pulmonary fibrosis in adults: an official ATS/ERS/JRS/ALAT clinical practice guideline. Am J Respir Crit Care Med. (2022) 205:e18–47. doi: 10.1164/rccm.202202-0399ST PMC9851481 35486072

[8] Mathai SC Danoff SK. Management of interstitial lung disease associated with connective tissue disease. BMJ. (2016) 352:h6819. doi: 10.1136/bmj.h6819 26912511 PMC6887350

[9] Antoniou KM Margaritopoulos G Economidou F Siafakas NM. Pivotal clinical dilemmas in collagen vascular diseases associated with interstitial lung involvement. Eur Respir J. (2009) 33:882–96. doi: 10.1183/09031936.00152607 19336591

[10] Schultze JL Aschenbrenner AC. Systems immunology allows a new view on human dendritic cells. Semin Cell Dev Biol. (2019) 86:15–23. doi: 10.1016/j.semcdb.2018.02.017 29448068

[11] Stephenson E Reynolds G Botting RA Calero-Nieto FJ Morgan MD Tuong ZK. Single-cell multi-omics analysis of the immune response in COVID-19. Nat Med. (2021) 27:904–16. doi: 10.1038/s41591-021-01329-2 PMC8121667 33879890

[12] Valenzi E Bulik M Tabib T Morse C Sembrat J Trejo Bittar H. Single-cell analysis reveals fibroblast heterogeneity and myofibroblasts in systemic sclerosis-associated interstitial lung disease. Ann Rheum Dis. (2019) 78:1379–87. doi: 10.1136/annrheumdis-2018-214865 PMC7255436 31405848

[13] Fujii W Kapellos TS Bassler K Handler K Holsten L Knoll R. Alveolar macrophage transcriptomic profiling in COPD shows major lipid metabolism changes. ERJ Open Res. (2021) 7(3):00915-2020. doi: 10.1183/23120541.00915-2020 PMC8435801 34527724

[14] Bassler K Fujii W Kapellos TS Dudkin E Reusch N Horne A. Alveolar macrophages in early stage COPD show functional deviations with properties of impaired immune activation. Front Immunol. (2022) 13:917232. doi: 10.3389/fimmu.2022.917232 35979364 PMC9377018

[15] Morse C Tabib T Sembrat J Buschur KL Bittar HT Valenzi E. Proliferating SPP1/MERTK-expressing macrophages in idiopathic pulmonary fibrosis. Eur Respir J. (2019) 54(2):1802441. doi: 10.1183/13993003.02441-2018 PMC8025672 31221805

[16] Gao X Jia G Guttman A DePianto DJ Morshead KB Sun KH. Osteopontin links myeloid activation and disease progression in systemic sclerosis. Cell Rep Med. (2020) 1:100140. doi: 10.1016/j.xcrm.2020.100140 33294861 PMC7691442

[17] Travis WD Costabel U Hansell DM King TE Jr. Lynch DA Nicholson AG. An official American Thoracic Society/European Respiratory Society statement: Update of the international multidisciplinary classification of the idiopathic interstitial pneumonias. Am J Respir Crit Care Med. (2013) 188:733–48. doi: 10.1164/rccm.201308-1483ST PMC5803655 24032382

[18] van der Linden MP Knevel R Huizinga TW van der Helm-van Mil AH. Classification of rheumatoid arthritis: comparison of the 1987 American College of Rheumatology criteria and the 2010 American College of Rheumatology/European League Against Rheumatism criteria. Arthritis Rheumatol. (2011) 63:37–42. doi: 10.1002/art.30100 20967854

[19] van den Hoogen F Khanna D Fransen J Johnson SR Baron M Tyndall A. 2013 Classification criteria for systemic sclerosis: an American College of Rheumatology/European League against Rheumatism collaborative initiative. Arthritis Rheum. (2013) 65:2737–47. doi: 10.1002/art.38098 PMC3930146 24122180

[20] Shiboski CH Shiboski SC Seror R Criswell LA Labetoulle M Lietman TM. 2016 American College of Rheumatology/European League Against Rheumatism classification criteria for primary Sjögren’s syndrome: A consensus and data-driven methodology involving three international patient cohorts. Ann Rheum Dis. (2017) 76:9–16. doi: 10.1136/annrheumdis-2016-210571 27789466

[21] Lundberg IE Tjarnlund A Bottai M Werth VP Pilkington C Visser M. 2017 European League Against Rheumatism/American College of Rheumatology classification criteria for adult and juvenile idiopathic inflammatory myopathies and their major subgroups. Ann Rheum Dis. (2017) 76:1955–64. doi: 10.1136/annrheumdis-2017-211468 PMC5736307 29079590

[22] Suppiah R Robson JC Grayson PC Ponte C Craven A Khalid S. 2022 American College of Rheumatology/European Alliance of Associations for Rheumatology classification criteria for microscopic polyangiitis. Ann Rheum Dis. (2022) 81:321–6. doi: 10.1136/annrheumdis-2021-221796 35110332

[23] Robson JC Grayson PC Ponte C Suppiah R Craven A Judge A. 2022 American College of Rheumatology/European Alliance of Associations for Rheumatology classification criteria for granulomatosis with polyangiitis. Ann Rheum Dis. (2022) 81:315–20. doi: 10.1136/annrheumdis-2021-221795 35110333

[24] Grayson PC Ponte C Suppiah R Robson JC Craven A Judge A. 2022 American college of rheumatology/European alliance of associations for rheumatology classification criteria for eosinophilic granulomatosis with polyangiitis. Ann Rheum Dis. (2022) 81:309–14. doi: 10.1136/annrheumdis-2021-221794 35110334

[25] Gierahn TM Wadsworth MH 2nd Hughes TK Bryson BD Butler A Satija R. Seq-Well: portable, low-cost RNA sequencing of single cells at high throughput. Nat Methods. (2017) 14:395–8. doi: 10.1038/nmeth.4179 PMC5376227 28192419

[26] Corleis B Tzouanas CN Wadsworth MH 2nd Cho JL Linder AH Schiff AE. Tobacco smoke exposure recruits inflammatory airspace monocytes that establish permissive lung niches for Mycobacterium tuberculosis. Sci Transl Med. (2023) 15:eadg3451. doi: 10.1126/scitranslmed.adg3451 38055798 PMC12289333

[27] McGinnis CS Murrow LM Gartner ZJ. DoubletFinder: doublet detection in single-cell RNA sequencing data using artificial nearest neighbors. Cell Syst. (2019) 8:329–37 e4. doi: 10.1016/j.cels.2019.03.003 30954475 PMC6853612

[28] Papazoglou A Huang M Bulik M Lafyatis A Tabib T Morse C. Epigenetic regulation of profibrotic macrophages in systemic sclerosis-associated interstitial lung disease. Arthritis Rheumatol. (2022) 74:2003–14. doi: 10.1002/art.42286 PMC9771864 35849803

[29] Zhang P Cao L Zhou R Yang X Wu M. The lncRNA Neat1 promotes activation of inflammasomes in macrophages. Nat Commun. (2019) 10:1495. doi: 10.1038/s41467-019-09482-6 30940803 PMC6445148

[30] Travaglini KJ Nabhan AN Penland L Sinha R Gillich A Sit RV. A molecular cell atlas of the human lung from single-cell RNA sequencing. Nature. (2020) 587:619–25. doi: 10.1038/s41586-020-2922-4 PMC7704697 33208946

[31] Sikkema L Ramirez-Suastegui C Strobl DC Gillett TE Zappia L Madissoon E. An integrated cell atlas of the lung in health and disease. Nat Med. (2023) 29:1563–77. doi: 10.1038/s41591-023-02327-2 PMC10287567 37291214

[32] Luppi F Sebastiani M Silva M Sverzellati N Cavazza A Salvarani C. Interstitial lung disease in Sjögren’s syndrome: a clinical review. Clin Exp Rheumatol. (2020) 38 Suppl 126:291–300. 33095142

[33] Zhang M Liu S Miao Z Han F Gottardo R Sun W. IDEAS: individual level differential expression analysis for single-cell RNA-seq data. Genome Biol. (2022) 23:33. doi: 10.1186/s13059-022-02605-1 35073995 PMC8784862

[34] Koduri G Solomon JJ. Identification, monitoring and management of rheumatoid arthritis-associated interstitial lung disease. Arthritis Rheumatol. (2023) 75(12):2067-77. doi: 10.1002/art.42640 37395725

[35] Distler O Assassi S Cottin V Cutolo M Danoff SK Denton CP. Predictors of progression in systemic sclerosis patients with interstitial lung disease. Eur Respir J. (2020) 55(5):1902026. doi: 10.1183/13993003.02026-2019 PMC7236865 32079645

[36] Ogawa T Shichino S Ueha S Ogawa S Matsushima K. Complement protein C1q activates lung fibroblasts and exacerbates silica-induced pulmonary fibrosis in mice. Biochem Biophys Res Commun. (2022) 603:88–93. doi: 10.1016/j.bbrc.2022.02.090 35278885

[37] Dalavanga YA Voulgari PV Georgiadis AN Leontaridi C Katsenos S Vassiliou M. Lymphocytic alveolitis: A surprising index of poor prognosis in patients with primary Sjogren’s syndrome. Rheumatol Int. (2006) 26:799–804. doi: 10.1007/s00296-005-0092-1 16344933

[38] Hugle T Hogan V White KE van Laar JM. Mast cells are a source of transforming growth factor beta in systemic sclerosis. Arthritis Rheumatol. (2011) 63:795–9. doi: 10.1002/art.30190 21360509

[39] Shimbori C Upagupta C Bellaye PS Ayaub EA Sato S Yanagihara T. Mechanical stress-induced mast cell degranulation activates TGF-beta1 signalling pathway in pulmonary fibrosis. Thorax. (2019) 74:455–65. doi: 10.1136/thoraxjnl-2018-211516 30808717

[40] Garcia JG Parhami N Killam D Garcia PL Keogh BA. Bronchoalveolar lavage fluid evaluation in rheumatoid arthritis. Am Rev Respir Dis. (1986) 133:450–4. doi: 10.1164/arrd.1986.133.3.450 3485395

[41] Jiang X. The mechanisms and therapeutic potential of long noncoding RNA NEAT1 in fibrosis. Clin Exp Med. (2023) 23(7):3339-47. doi: 10.1007/s10238-023-01191-1 37740135

[42] Rizzo C Grasso G Destro Castaniti GM Ciccia F Guggino G. Primary sjogren syndrome: focus on innate immune cells and inflammation. Vaccines (Basel). (2020) 8(2): 272. doi: 10.3390/vaccines8020272 PMC7349953 32503132

[43] Brkic Z Maria NI van Helden-Meeuwsen CG van de Merwe JP van Daele PL Dalm VA. Prevalence of interferon type I signature in CD14 monocytes of patients with Sjogren’s syndrome and association with disease activity and BAFF gene expression. Ann Rheum Dis. (2013) 72:728–35. doi: 10.1136/annrheumdis-2012-201381 PMC3618683 22736090

[44] Peng Y Wu X Zhang S Deng C Zhao L Wang M. The potential roles of type I interferon activated neutrophils and neutrophil extracellular traps (NETs) in the pathogenesis of primary Sjogren’s syndrome. Arthritis Res Ther. (2022) 24:170. doi: 10.1186/s13075-022-02860-4 35854322 PMC9295258

[45] Nezos A Gravani F Tassidou A Kapsogeorgou EK Voulgarelis M Koutsilieris M. Type I and II interferon signatures in Sjogren’s syndrome pathogenesis: Contributions in distinct clinical phenotypes and Sjogren’s related lymphomagenesis. J Autoimmun. (2015) 63:47–58. doi: 10.1016/j.jaut.2015.07.002 26183766 PMC4564326

[46] Baruah P Dumitriu IE Malik TH Cook HT Dyson J Scott D. C1q enhances IFN-gamma production by antigen-specific T cells via the CD40 costimulatory pathway on dendritic cells. Blood. (2009) 113:3485–93. doi: 10.1182/blood-2008-06-164392 19171874

[47] van de Bovenkamp FS Dijkstra DJ van Kooten C Gelderman KA Trouw LA. Circulating C1q levels in health and disease, more than just a biomarker. Mol Immunol. (2021) 140:206–16. doi: 10.1016/j.molimm.2021.10.010 34735869

[48] Gono T Okazaki Y Kuwana M. Antiviral proinflammatory phenotype of monocytes in anti-MDA5 antibody-associated interstitial lung disease. Rheumatol (Oxford). (2022) 61:806–14. doi: 10.1093/rheumatology/keab371 33890985

[49] Walsh RJ Kong SW Yao Y Jallal B Kiener PA Pinkus JL. Type I interferon-inducible gene expression in blood is present and reflects disease activity in dermatomyositis and polymyositis. Arthritis Rheumatol. (2007) 56:3784–92. doi: 10.1002/art.22928 PMC2443782 17968926

[50] Wienke J Bellutti Enders F Lim J Mertens JS van den Hoogen LL Wijngaarde CA. Galectin-9 and CXCL10 as biomarkers for disease activity in juvenile dermatomyositis: A longitudinal cohort study and multicohort validation. Arthritis Rheumatol. (2019) 71:1377–90. doi: 10.1002/art.40881 PMC6973145 30861625

[51] Neupane AS Willson M Chojnacki AK Vargas ESCF Morehouse C Carestia A. Patrolling alveolar macrophages conceal bacteria from the immune system to maintain homeostasis. Cell. (2020) 183:110–25 e11. doi: 10.1016/j.cell.2020.08.020 32888431

[52] Akiyama M Kaneko Y. Pathogenesis, clinical features, and treatment strategy for rheumatoid arthritis-associated interstitial lung disease. Autoimmun Rev. (2022) 21:103056. doi: 10.1016/j.autrev.2022.103056 35121155

[53] Jordan S Distler JH Maurer B Huscher D van Laar JM Allanore Y. Effects and safety of rituximab in systemic sclerosis: an analysis from the European Scleroderma Trial and Research (EUSTAR) group. Ann Rheum Dis. (2015) 74:1188–94. doi: 10.1136/annrheumdis-2013-204522 24442885

[54] Kropski JA Blackwell TS. Endoplasmic reticulum stress in the pathogenesis of fibrotic disease. J Clin Invest. (2018) 128:64–73. doi: 10.1172/JCI93560 29293089 PMC5749533

[55] Lenna S Farina AG Martyanov V Christmann RB Wood TA Farber HW. Increased expression of endoplasmic reticulum stress and unfolded protein response genes in peripheral blood mononuclear cells from patients with limited cutaneous systemic sclerosis and pulmonary arterial hypertension. Arthritis Rheumatol. (2013) 65:1357–66. doi: 10.1002/art.37891 PMC3636187 23400395

[56] Kuzumi A Yoshizaki A Ebata S Fukasawa T Yoshizaki-Ogawa A Asano Y. Serum TARC levels in patients with systemic sclerosis: clinical association with interstitial lung disease. J Clin Med. (2021) 10(4):660. doi: 10.3390/jcm10040660 PMC7915627 33572144

[57] Jamaluddin MS Weakley SM Yao Q Chen C. Resistin: functional roles and therapeutic considerations for cardiovascular disease. Br J Pharmacol. (2012) 165:622–32. doi: 10.1111/j.1476-5381.2011.01369.x PMC3315035 21545576

[58] Jiang S Park DW Tadie JM Gregoire M Deshane J Pittet JF. Human resistin promotes neutrophil proinflammatory activation and neutrophil extracellular trap formation and increases severity of acute lung injury. J Immunol. (2014) 192:4795–803. doi: 10.4049/jimmunol.1302764 PMC4018664 24719460

[59] Nakazawa D Masuda S Tomaru U Ishizu A. Pathogenesis and therapeutic interventions for ANCA-associated vasculitis. Nat Rev Rheumatol. (2019) 15:91–101. doi: 10.1038/s41584-018-0145-y 30542206

[60] Hirano A Fujii W Sakashita A Baßler K Kadoya M Omoto A. POS0151 Single-cell RNA sequencing of bronchoalveolar lavage fluid and blood reveals disease-specific characteristics of immune cells in connective tissue disease-associated interstitial lung disease patients. Ann Rheumatic Diseases. (2023) 82:296–7. doi: 10.1136/annrheumdis-2023-eular.2875

