# Supplementary material for: Immunological characteristics of bronchoalveolar lavage fluid and blood across connective tissue disease-associated interstitial lung diseases
Source: Front Immunol. 2024 Oct 25;15:1408880. doi: 10.3389/fimmu.2024.1408880 (PMC11543407; doi:10.3389/fimmu.2024.1408880)
Supplement: Supplementary file 1 [file DataSheet1.pdf]

Supplementary materials

Supplementary Figure 1

A

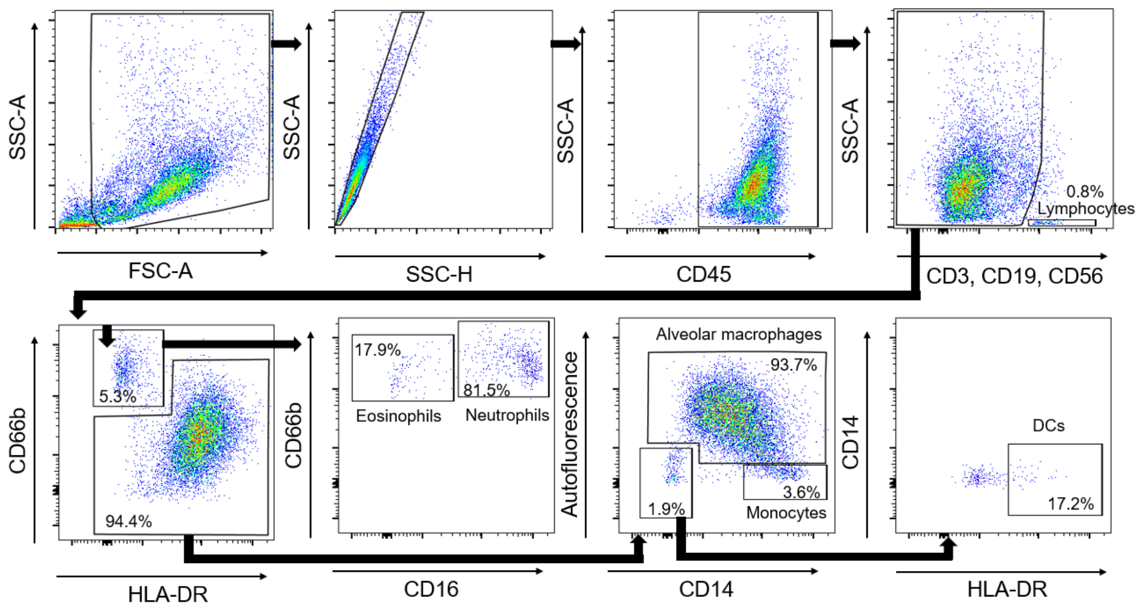

B

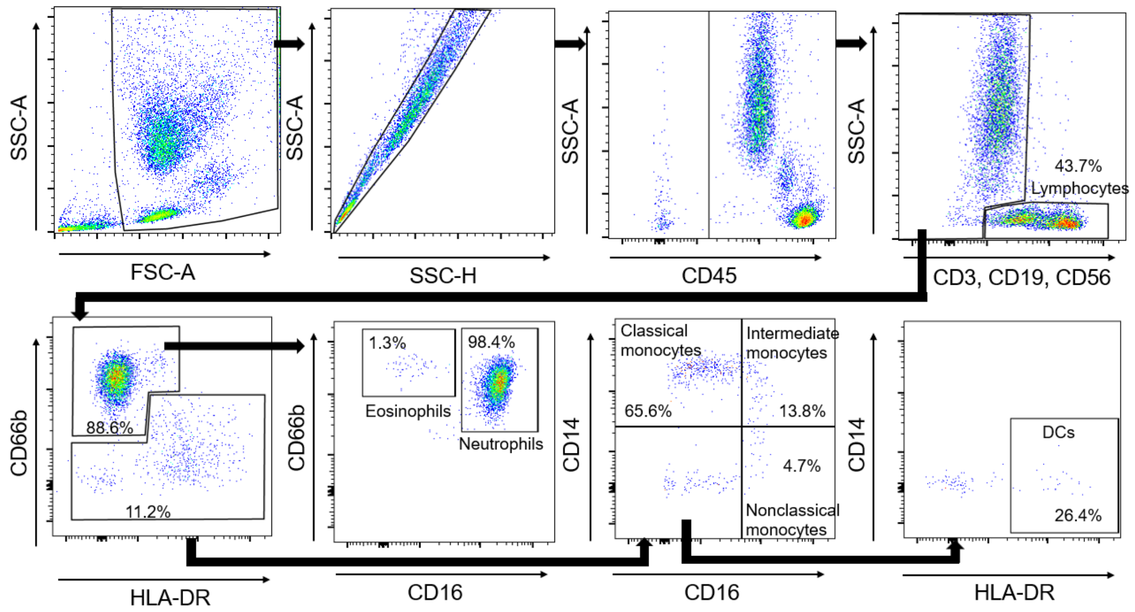

C

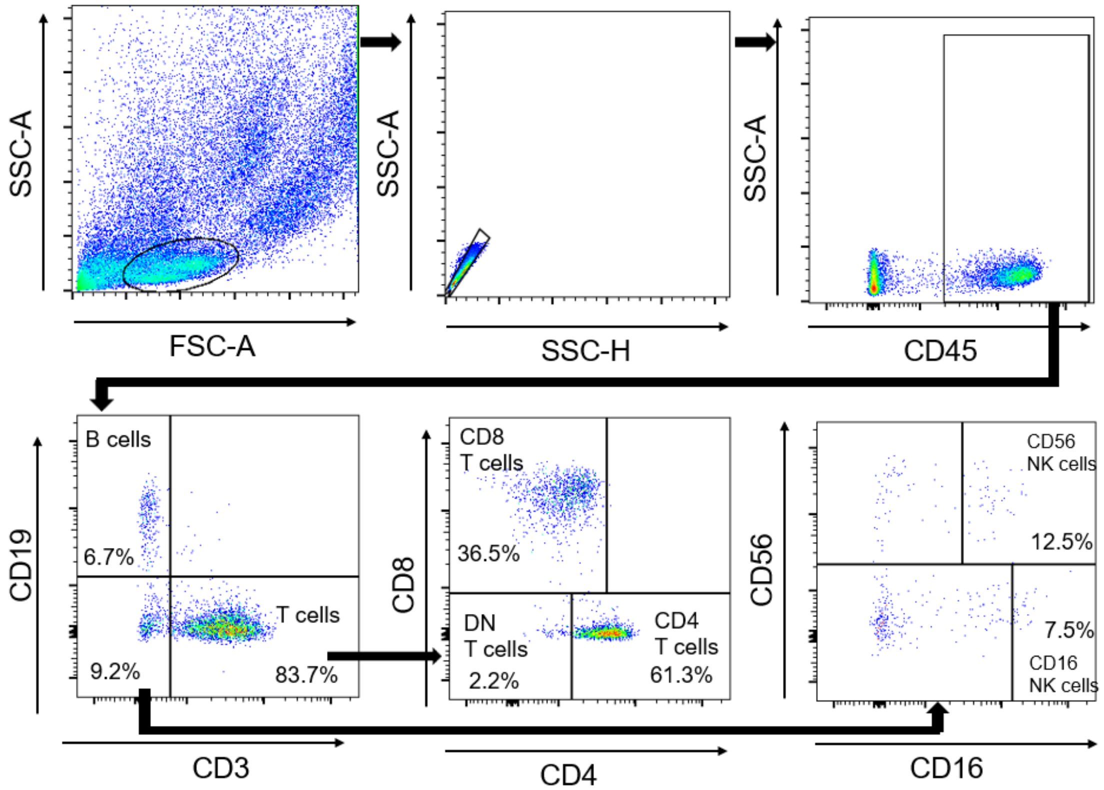
SSC-A
FSC-A
SSC-A
SSC-H
SSC-A
CD45
CD19
B cells
6.7%
T cells
9.2%
83.7%
CD3
CD8
CD8
T cells
36.5%
DN
T cells
2.2%
CD4
T cells
61.3%
CD4
CD56
CD56
NK cells
12.5%
7.5%
CD16
NK cells
CD16

D

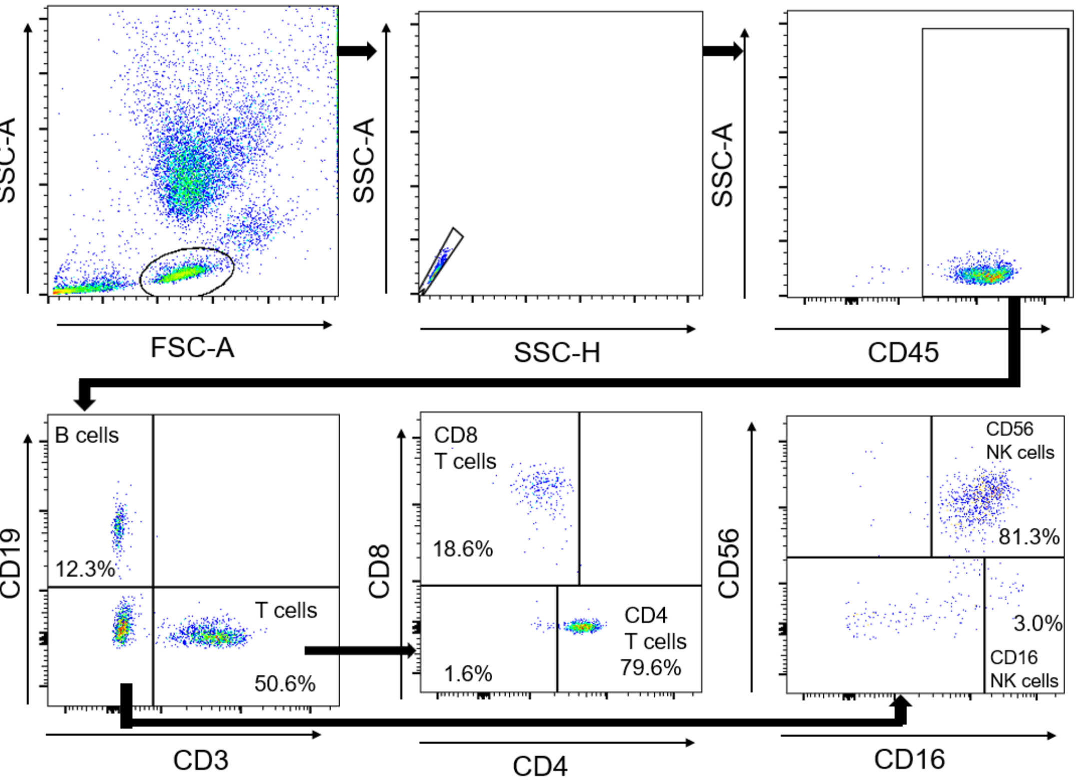
SSC-A
FSC-A
SSC-A
SSC-H
SSC-A
CD45
CD19
B cells
12.3%
T cells
50.6%
CD3
CD8
CD8
T cells
18.6%
1.6%
CD4
T cells
79.6%
CD4
CD56
CD56
NK cells
81.3%
3.0%
CD16
NK cells
CD16

Gating Strategy. (A) For bronchoalveolar lavage fluid (BALF) myeloid cells. (B) For blood myeloid cells. (C) For BALF lymphocytes. (D) For blood lymphocytes. Abbreviations: SSC-A, side scatter area; FSC-A, forward scatter area; SSC-H, side scatter height; DN, double negative.

Supplementary Figure 2

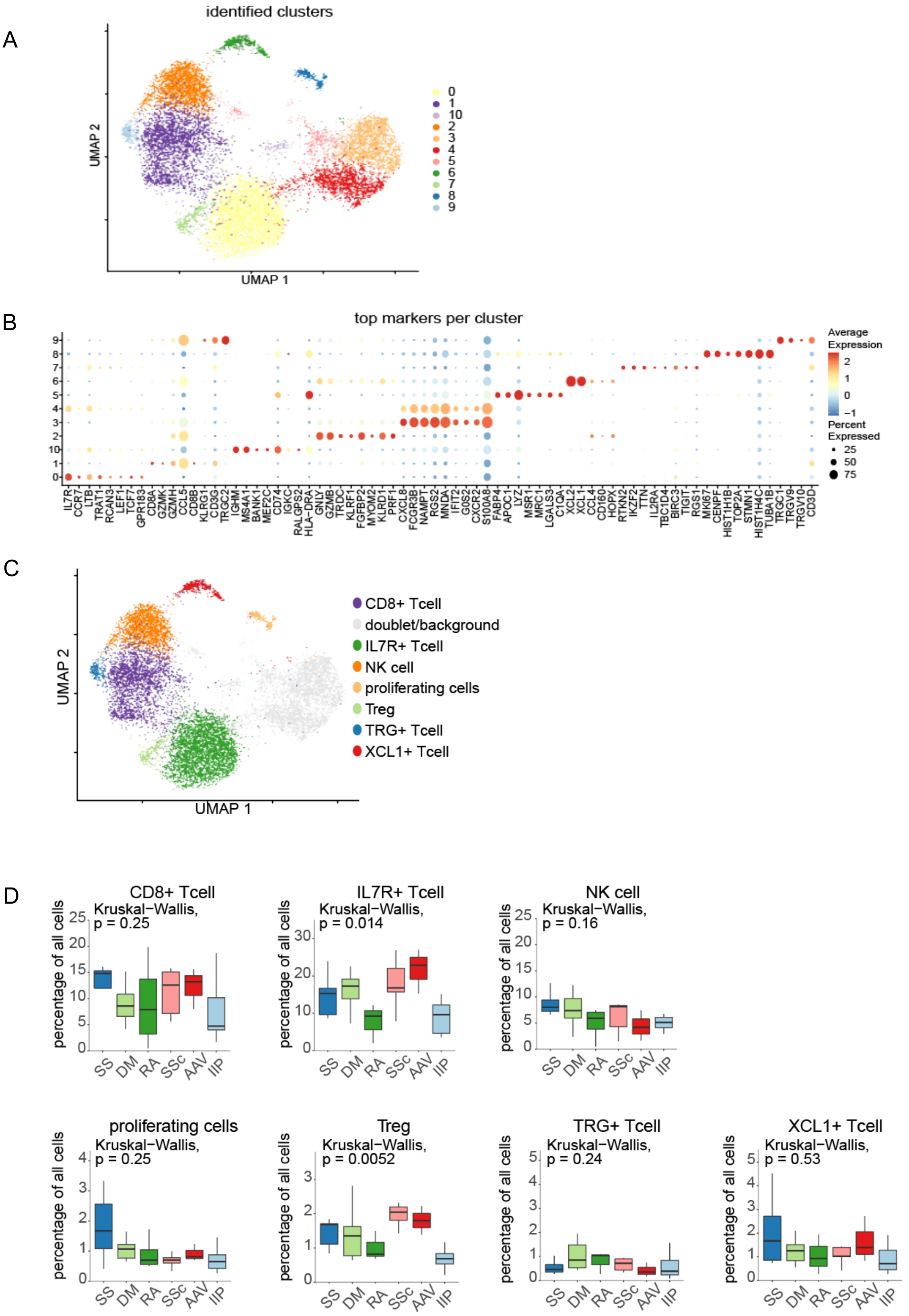

T/NK cell subtypes in the blood. (A) UMAP representation of integrated blood data obtained from only the fraction of T/NK cells in the blood. The colors and numbers correspond to the identified main clusters. (B) Dot plots show the top marker genes per cluster. (C) Cell type annotation of the integrated blood data according to the step annotation approach. (D) Comparison of the proportion of each subtype of T/NK cells in the blood among patients with various diseases. The Kruskal–Wallis test followed by the Steel–Dwass test was performed for multi-condition comparisons. Statistical significance was set at $p < 0.05$. Several T-cell fractions that appeared to differ among diseases using the Kruskal–Wallis test showed no obvious significant differences in the Steel–Dwass test.

Abbreviations: SS, Sjögren’s syndrome; DM, dermatomyositis; RA, rheumatoid arthritis; SSc, systemic sclerosis; AAV, ANCA-associated vasculitis; IIP, idiopathic interstitial pneumonia; IL7R, Interleukin 7 Receptor; NK, natural killer; TRG, T Cell Receptor Gamma Locus; XCL1, X-C Motif Chemokine Ligand 1.

Supplementary Figure 3

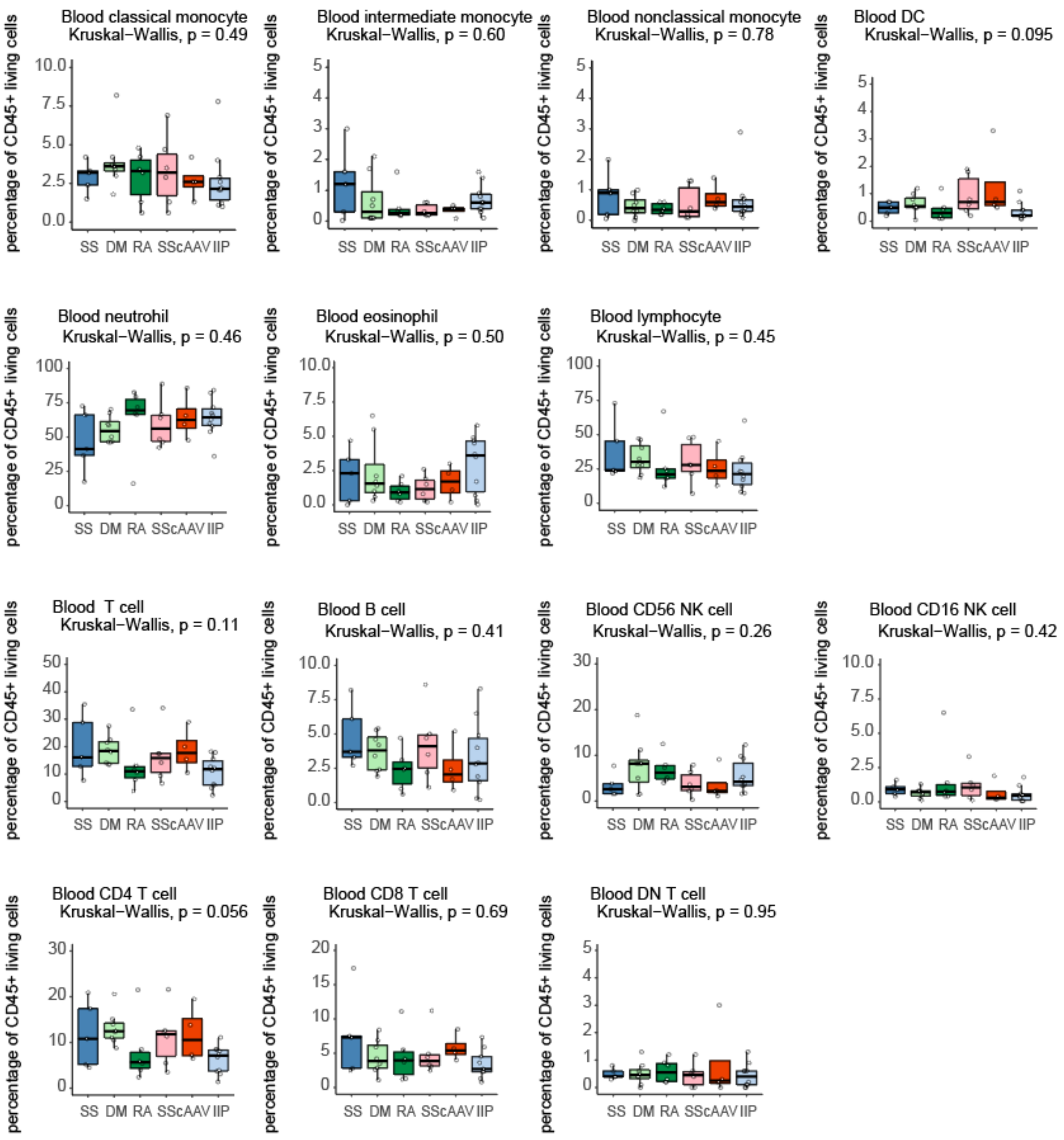

Major cell type cell proportion in the blood analyzed by multi-color flow cytometry (MCFC). We performed MCFC, and differences in cell proportion in the blood among patients with various diseases were not significant by the Kruskal–Wallis test. Statistical significance was set at $p < 0.05$. We gated $CD45^+$ living single cells and analyzed myeloid cells and lymphoid cells separately. For myeloid cells in the blood, we defined $CD3^-CD19^-CD56^-CD66b^+HLA\text{-}DR^-CD16^+$ cells as neutrophils, $CD3^-CD19^-CD56^-CD66b^+HLA\text{-}DR^-CD16^-$ as eosinophils, $CD3^-CD19^-CD56^-CD66b^-CD14^+CD16^-$ as

classical monocytes, $CD3^-CD19^-CD56^-CD66b^-CD14^+CD16^+$ as intermediate monocytes, $CD3^-CD19^-CD56^-CD66b^-CD14^-CD16^+$ as nonclassical monocytes, $CD3^-CD19^-CD56^-CD66b^-HLA\text{-}DR^+CD14^-CD16^-$ as dendritic cells, and $CD3^+CD19^+CD56^+$ as lymphocytes. For lymphoid cells in the blood, we defined $CD3^+CD19^-$ cells as T cells, $CD3^-CD19^+$ as B cells, $CD3^-CD19^-CD56^+$ as CD56 NK cells, $CD3^-CD19^-CD56^-CD16^+$ as CD16 NK cells, $CD3^+CD4^+CD8^-CD19^-$ cells as CD4 T cells, $CD3^+CD4^-CD8^+CD19^-$ as CD8 T cells, and $CD3^+CD4^-CD8^-CD19^-$ as double negative T cells. Abbreviations: SS, Sjögren's syndrome; DM, dermatomyositis; RA, rheumatoid arthritis; SSc, systemic sclerosis; AAV, ANCA-associated vasculitis; IIP, idiopathic interstitial pneumonia; DC, dendritic cell; NK, natural killer; DN, double-negative.

Supplementary Figure 4

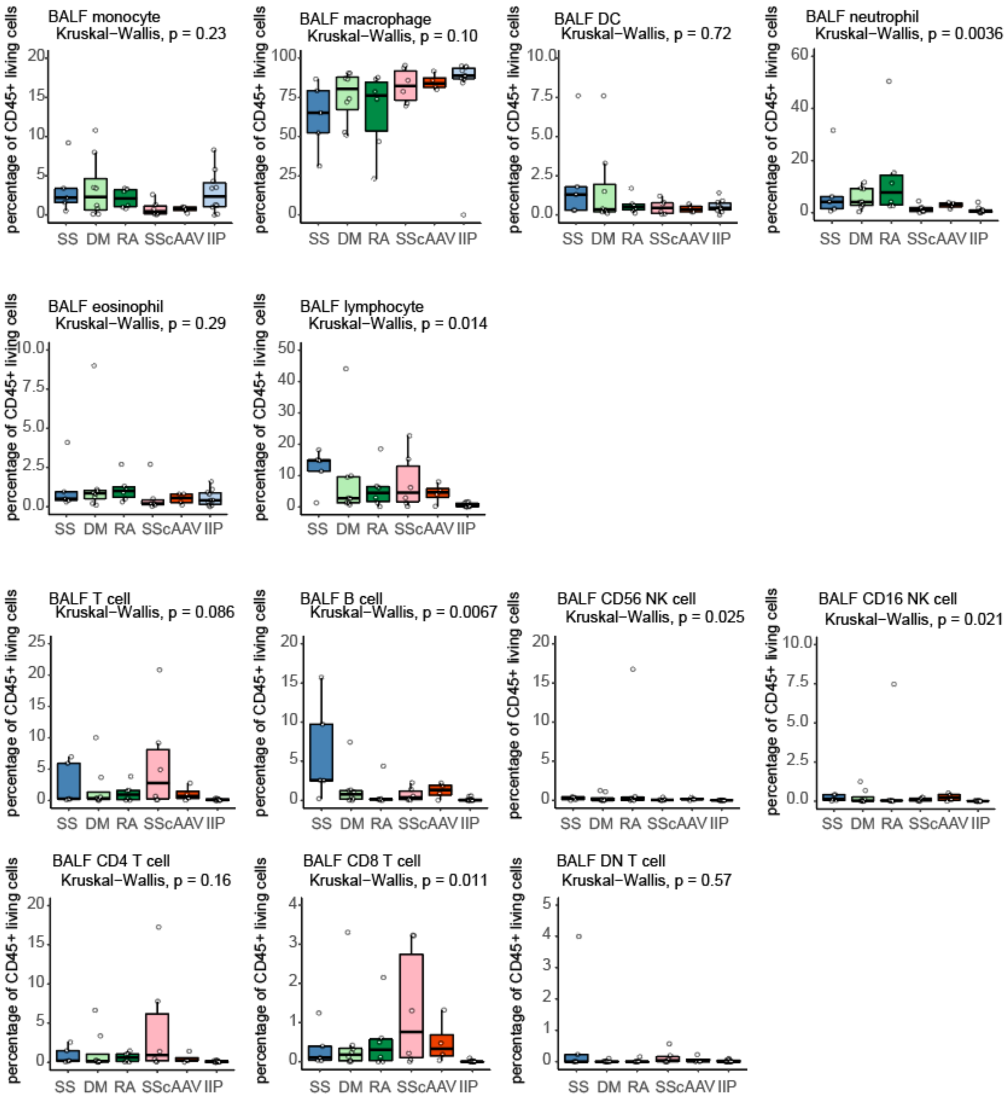

Major cell type cell proportion in the bronchoalveolar lavage fluid (BALF) analyzed by multi-color flow cytometry (MCFC). We performed MCFC and found that the differences in cell proportion in the BALF among patients with various diseases were similar to the results of single-cell RNA sequencing analysis. The Kruskal–Wallis test followed by the Steel–Dwass test was performed for multi-condition comparisons. Statistical significance was set at $p < 0.05$. The percentage of neutrophils in the BALF of patients with rheumatoid arthritis (RA)-associated interstitial lung disease (ILD) was

significantly increased compared to patients with idiopathic interstitial pneumonia (IIP) ($p = 0.028$). The percentage of other cell types exhibited no substantial variance among the diseases in the post hoc analysis. We gated CD45^{+} living single cells and analyzed myeloid cells and lymphoid cells separately. For myeloid cells in the BALF, we defined CD3^{-}CD19^{-}CD56^{-}CD66b^{+}HLA-DR^{-}CD16^{+} cells as neutrophils, CD3^{-}CD19^{-}CD56^{-}CD66b^{+}HLA-DR^{-}CD16^{-} as eosinophils, CD3^{-}CD19^{-}CD56^{-}CD66b^{-}HLA-DR^{+}autofluorescence^{+} as alveolar macrophages, CD3^{-}CD19^{-}CD56^{-}CD66b^{-}HLA-DR^{+}autofluorescence^{-}CD14^{+} as monocytes, CD3^{-}CD19^{-}CD56^{-}CD66b^{-}HLA-DR^{+}autofluorescence-CD14^{-} as dendritic cells, and CD3^{+}CD19^{+}CD56^{+} as lymphocytes. For lymphoid cells in the BALF, we defined CD3^{+}CD19^{-} cells as T cells, CD3^{-}CD19^{+} as B cells, CD3^{-}CD19^{-}CD56^{+} as CD56 NK cells, CD3^{-}CD19^{-}CD56^{-}CD16^{+} as CD16 NK cells, CD3^{+}CD4^{+}CD8^{-}CD19^{-} cells as CD4 T cells, CD3^{+}CD4^{-}CD8^{+}CD19^{-} as CD8 T cells, and CD3^{+}CD4^{-}CD8^{-}CD19^{-} as double negative T cells.

Abbreviations: SS, Sjögren's syndrome; DM, dermatomyositis; RA, rheumatoid arthritis; SSc, systemic sclerosis; AAV, ANCA-associated vasculitis; IIP, idiopathic interstitial pneumonia; DC, dendritic cell; NK, natural killer; DN, double-negative.

.

Supplementary Figure 5

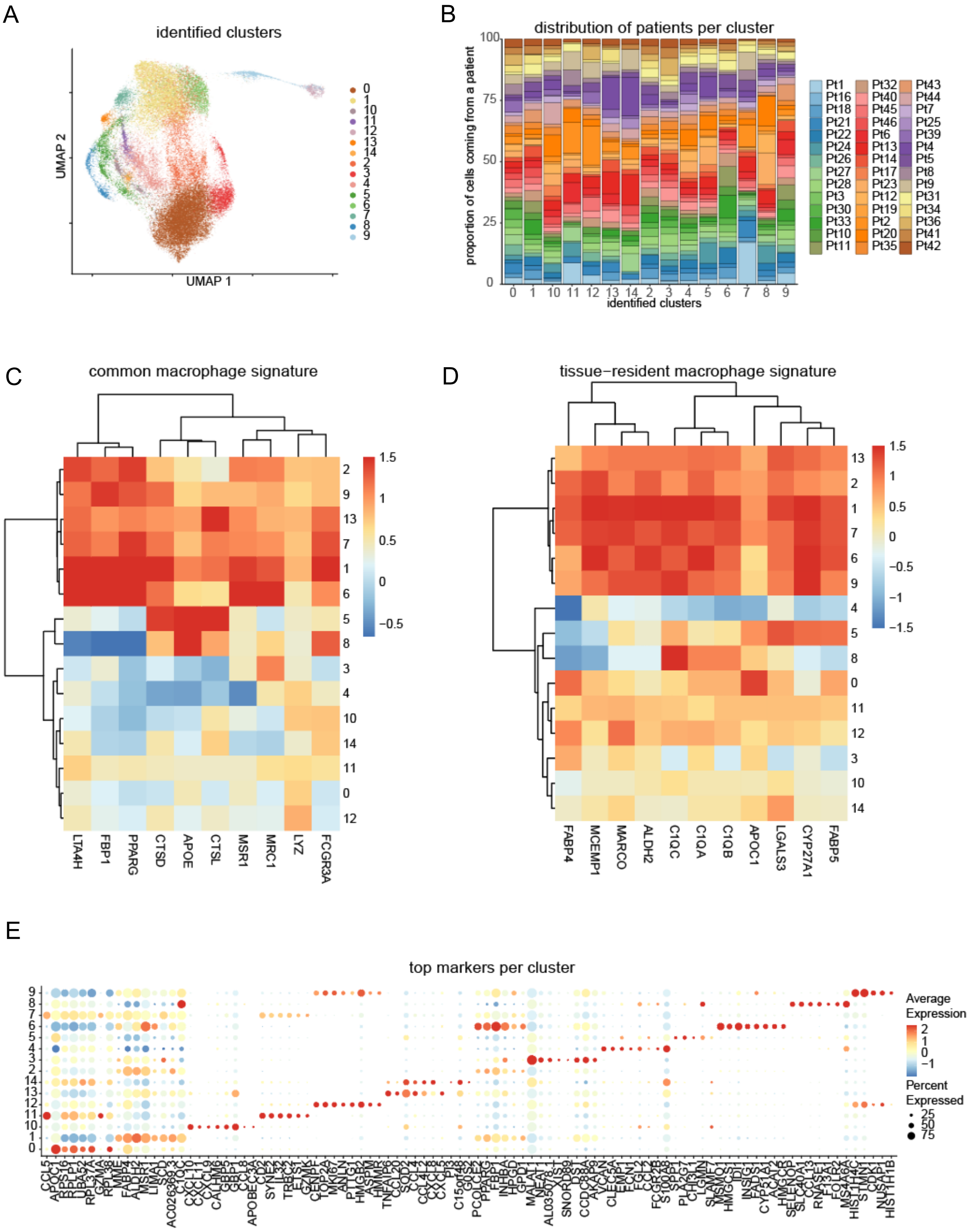

F

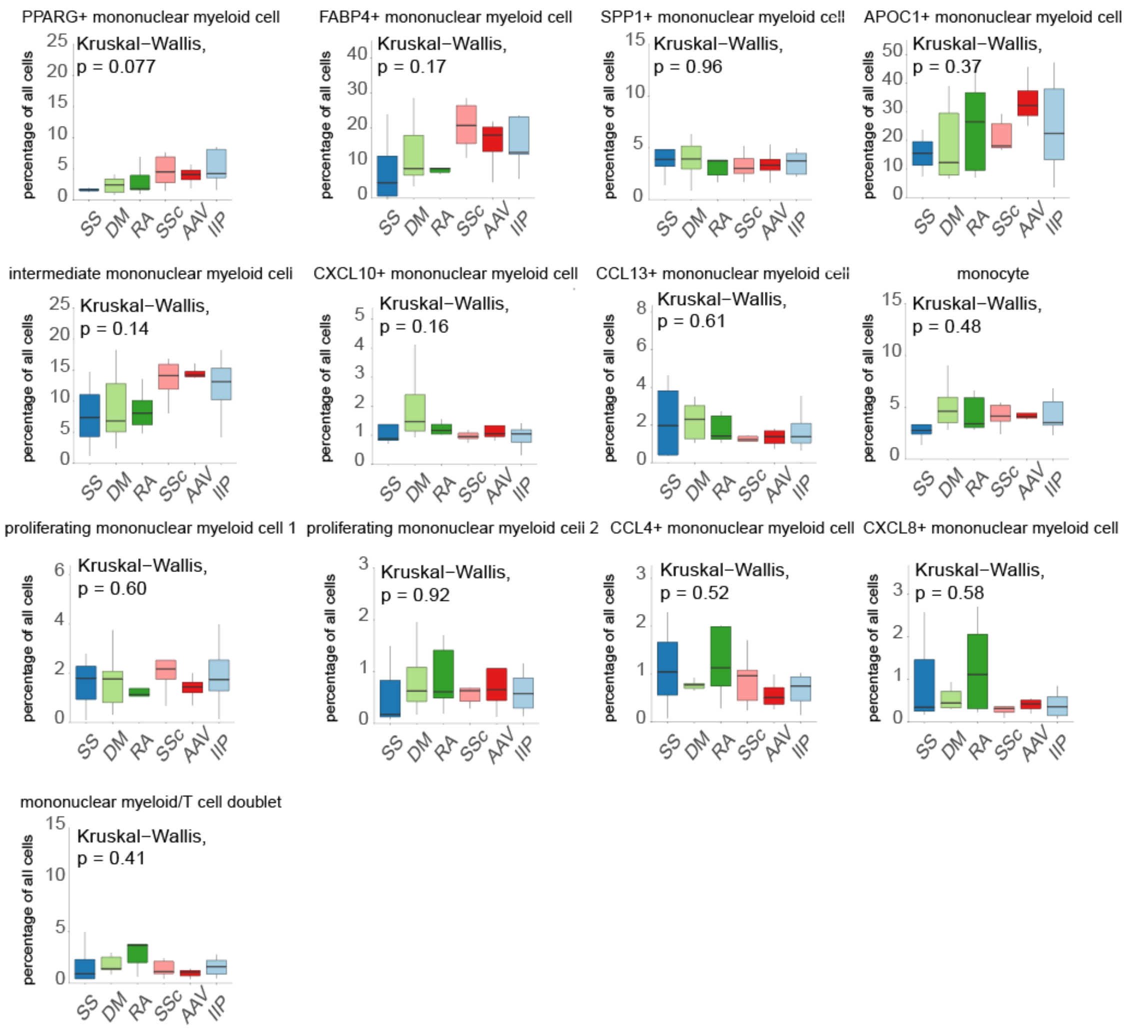

Subtypes of mononuclear myeloid cells in the bronchoalveolar lavage fluid (BALF). (A) UMAP representation of integrated BALF data obtained from only the fraction of mononuclear myeloid cells in the BALF. The colors and numbers correspond to the identified main clusters. 15 clusters were visualized. (B) Distribution of patients per cluster. The potential donor effect was evaluated and all 15 were significant clusters. (C) Heatmap of the common macrophage signature per cluster. (D) Heatmap of the tissue-resident macrophage signature per cluster. (E) Dot plots show the top marker genes per cluster. (F) Comparison of the proportion of each subtype of mononuclear myeloid cells in the BALF among patients with various diseases. The Kruskal–Wallis test was performed for multi-condition comparison. Statistical significance was set at $p < 0.05$.

Abbreviations: SS, Sjögren's syndrome; DM, dermatomyositis; RA, rheumatoid arthritis; SSc, systemic sclerosis; AAV, ANCA-associated vasculitis; IIP, idiopathic interstitial pneumonia; mononuclear myeloid cell, the fraction including monocytes, alveolar macrophages, and DCs; PPARG, Peroxisome Proliferator Activated Receptor Gamma; FABP4, Fatty Acid Binding Protein 4; SPP1, Secreted Phosphoprotein 1; APOC1, apolipoprotein C1; CXCL10, C-X-C Motif Chemokine Ligand 10; CCL13, C-C Motif Chemokine Ligand 13; CCL4, C-C Motif Chemokine Ligand 4; CXCL8, C-X-C Motif Chemokine Ligand 8.

Supplementary Figure 6

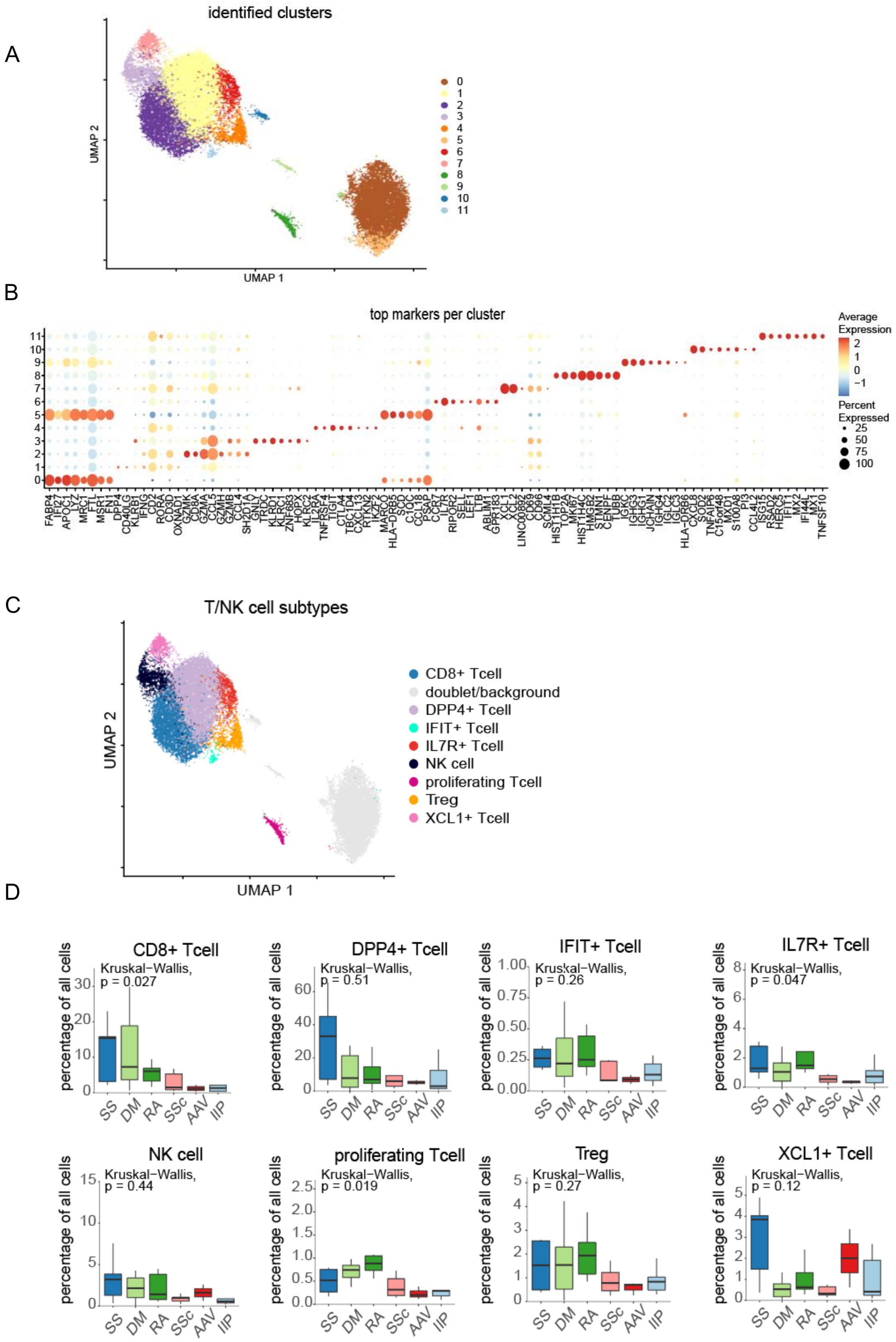

T/NK cell subtypes in the bronchoalveolar lavage fluid (BALF). (A) UMAP representation of integrated BALF data obtained from only the fraction of T/NK cells in the BALF. The colors and numbers correspond to the identified main clusters. (B) Dot plots show the top marker genes per cluster. (C) Cell type annotation of the integrated BALF data according to the step annotation approach. (D) Comparison of the proportion of each subtype of T/NK cells in the BALF among patients with various diseases. The Kruskal–Wallis test followed by the Steel–Dwass test was performed for multi-condition comparisons. Statistical significance was set at $p < 0.05$. Several T-cell fractions that appeared to differ among diseases using the Kruskal–Wallis test showed no obvious significant differences in the Steel–Dwass test.
Abbreviations: SS, Sjögren's syndrome; DM, dermatomyositis; RA, rheumatoid arthritis; SSc, systemic sclerosis; AAV, ANCA-associated vasculitis; IIP, idiopathic interstitial pneumonia; DPP4, Dipeptidyl Peptidase 4; IFIT, Interferon Induced proteins with Tetratricopeptide repeats; IL7R, Interleukin 7 Receptor; NK, natural killer; XCL1, X-C Motif Chemokine Ligand 1.

Supplementary Figure 7

A T/NK cells in BALF in patients with SS

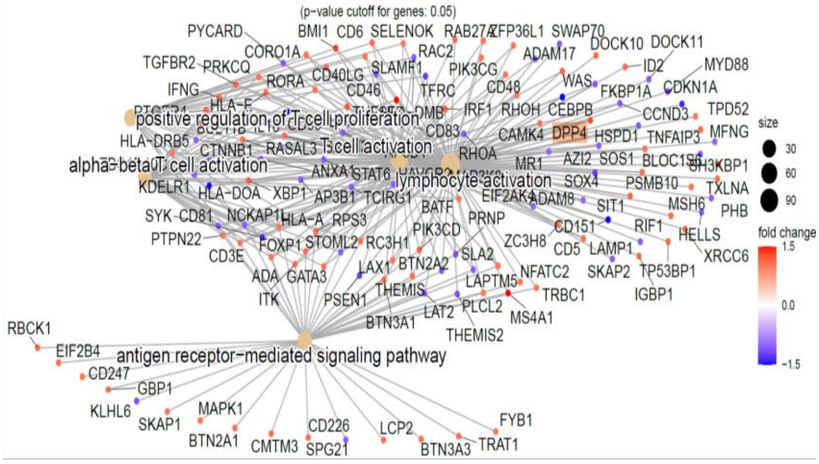

B B/plasma cells in BALF in patients with SS

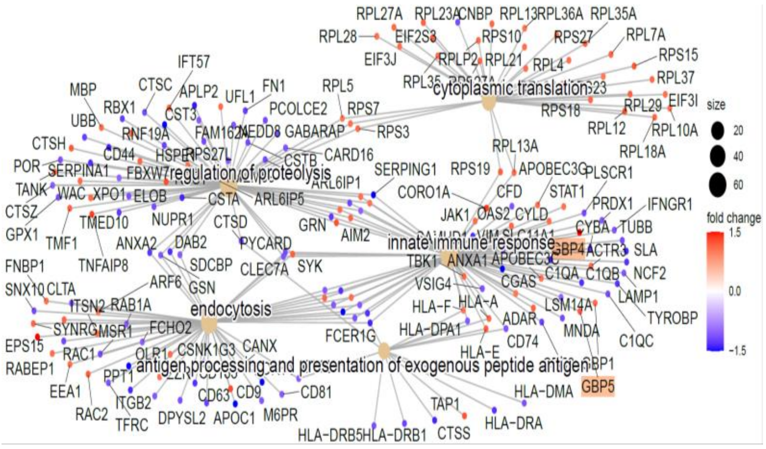

C Monocytes in blood in patients with SS

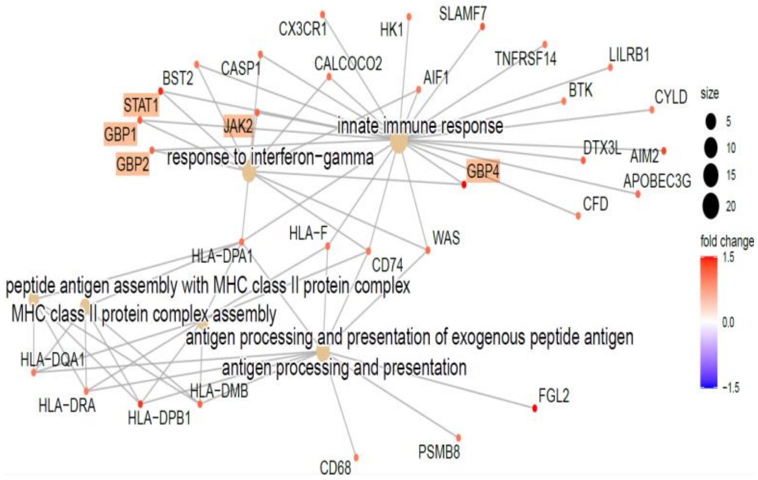

D Neutrophils in blood in patients with SS

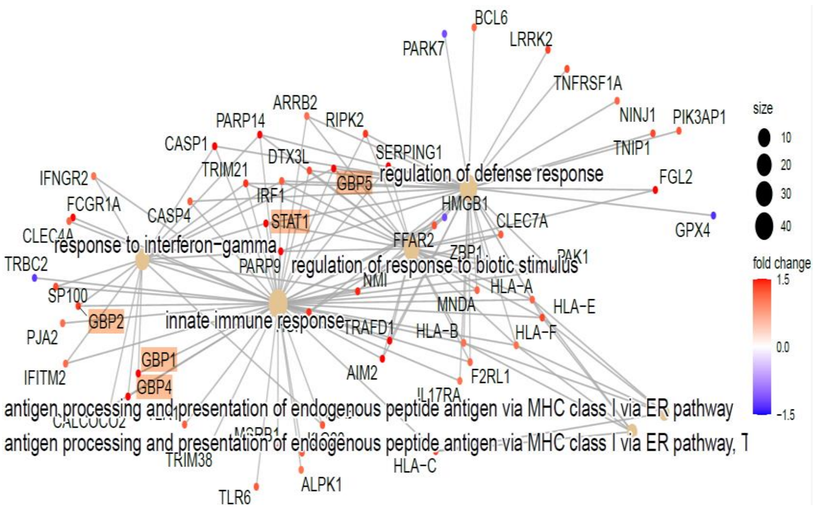

E T/NK cells in blood in patients with SS

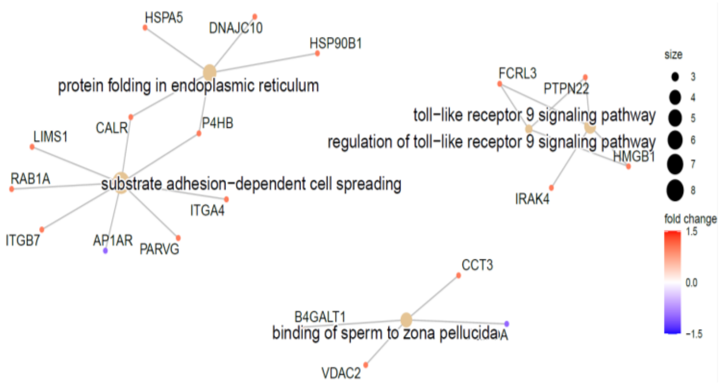

F B/plasma cells in blood in patients with SS

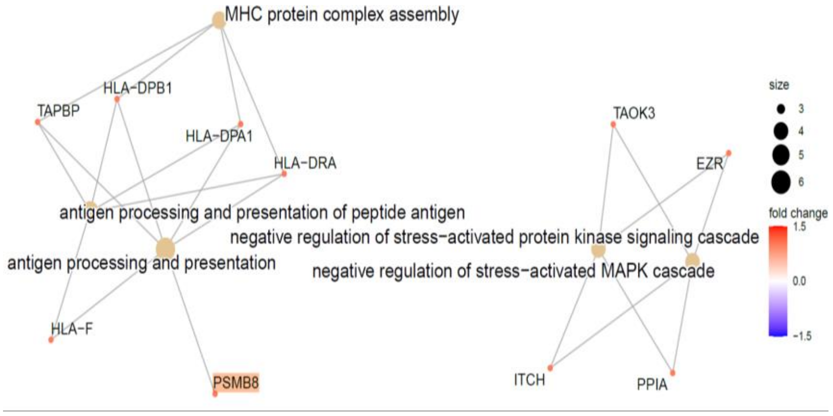

Gene ontology (GO) enrichment analysis of differentially expressed (DE) genes in the bronchoalveolar lavage fluid (BALF) and blood cells from patients with Sjögren's syndrome (SS)-associated interstitial lung disease (ILD). The most significantly enriched pathways in each immune cell were visualized by c-net. (A) T/NK cells in the BALF by c-net. (B) B/plasma cells in the BALF by c-net. (C) Monocytes in the blood by c-net. (D) Neutrophils in the blood by c-net. (E) T/NK cells in the blood by c-net. (F) B/plasma cells in the blood by c-net. The p-value cutoff for genes was set at 0.05 for T/NK cells and B/plasma cells in the BALF and blood and 0.01 for monocytes and neutrophils in the blood. C-net displays the linkage between genes and biological concepts as a network. The size of the dot corresponds to the number of DE genes belonging to the enriched GO pathway and the fold change shows the difference between SS-ILD relative to other diseases. Genes mentioned in the text are highlighted in parentheses.

Supplementary Figure 8

A Monocytes-macrophages in BALF in patients with DM

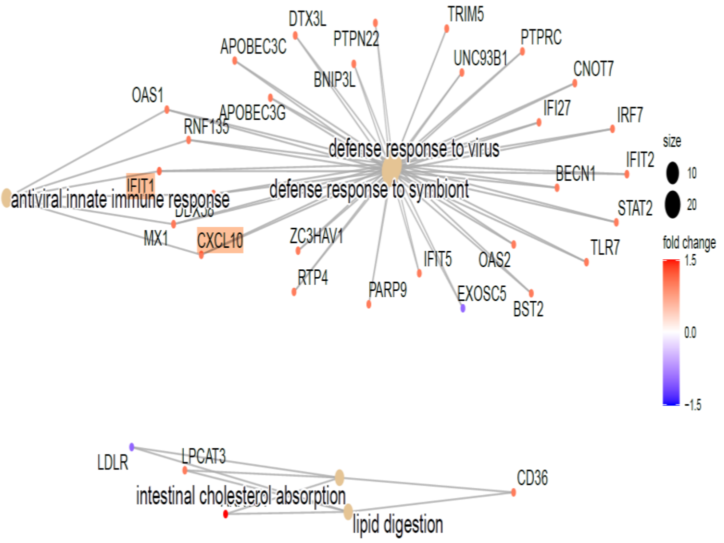

B Neutrophils in BALF in patients with DM

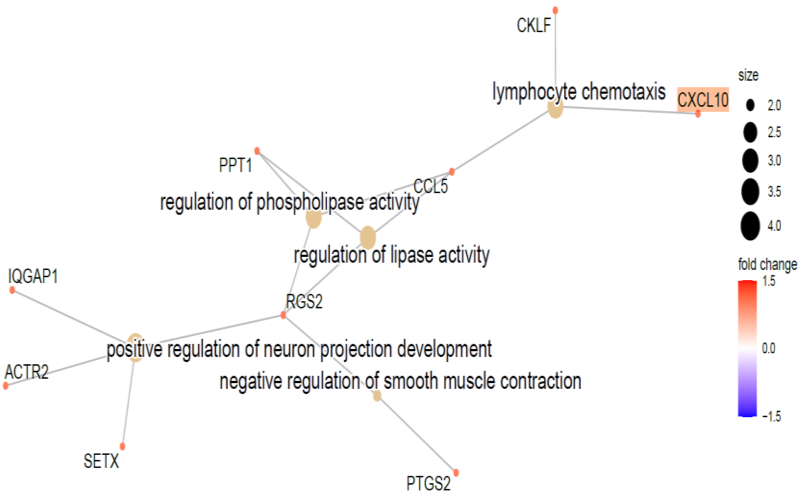

C T/NK cells in BALF in patients with DM

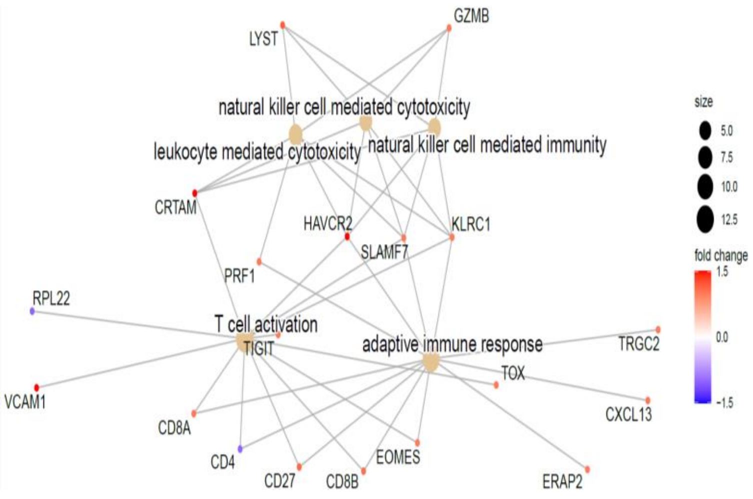

D B/plasma cells in BALF in patients with DM

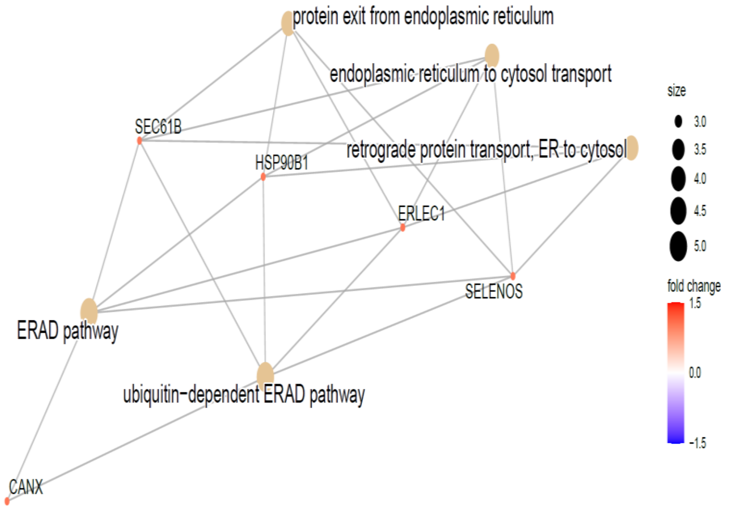

E Monocytes in blood in patients with DM

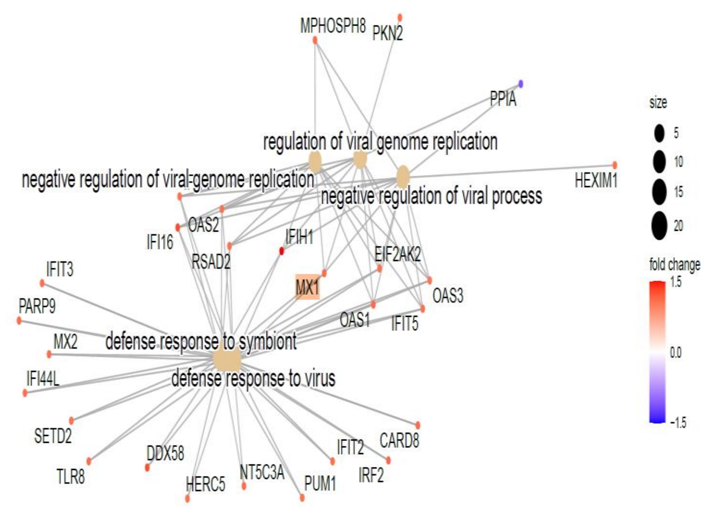

F Neutrophils in blood in patients with DM

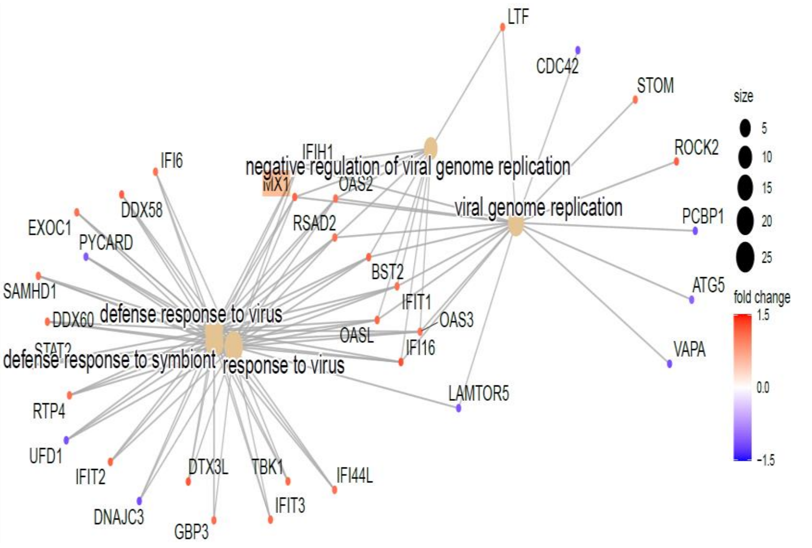

G T/NK cells in blood in patients with DM

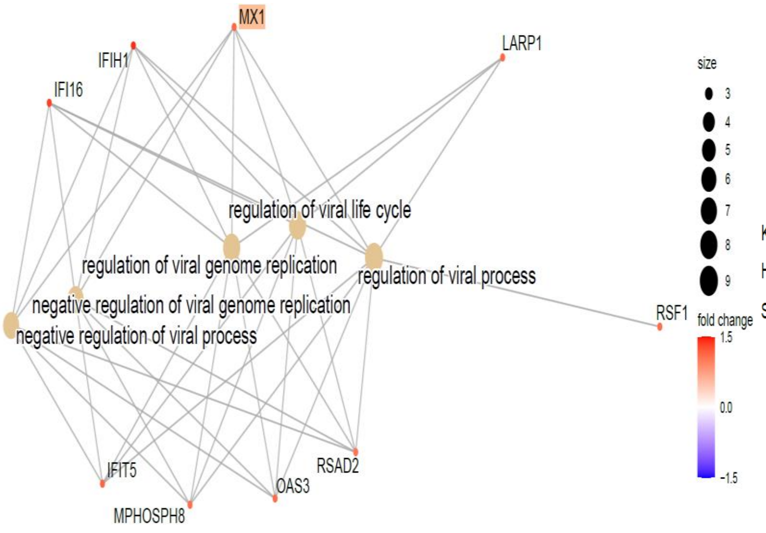

H B/plasma cells in blood in patients with DM

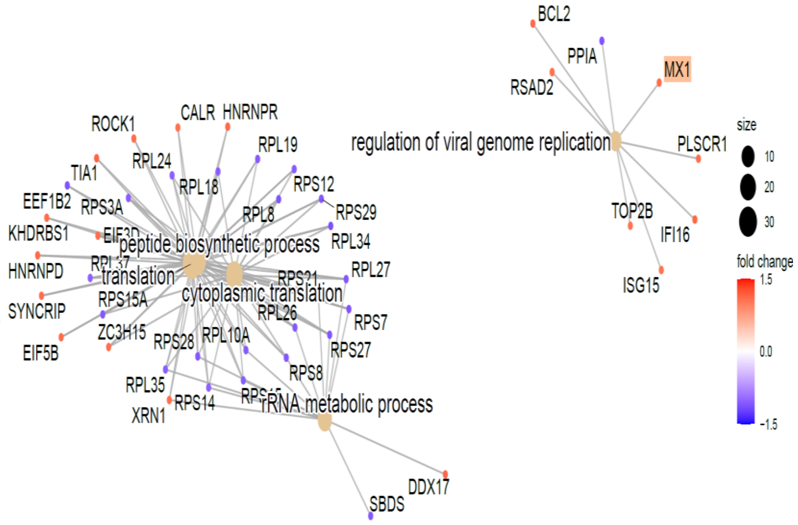

Gene ontology (GO) enrichment analysis of differentially expressed (DE) genes in the bronchoalveolar lavage fluid (BALF) and blood cells from patients with dermatomyositis (DM) -associated interstitial lung disease (ILD). The most significantly enriched pathways in each immune cell were visualized by c-net. (A) Monocytes-macrophages in the BALF by c-net. (B) Neutrophils in the BALF by c-net. (C) T/NK cells in the BALF by c-net. (D) B/plasma cells in the BALF by c-net. (E) Monocytes in the blood by c-net. (F) Neutrophils in the blood by c-net. (G) T/NK cells in the blood by c-net. (H) B/plasma cells in the blood by c-net. The p-value cutoff for genes was set at 0.05 for monocyte-macrophages and neutrophils in the BALF and monocytes, neutrophils, and B/plasma cells in the blood and 0.01 for T/NK cells and B/plasma cells in the BALF and T/NK cells in the blood. C-net displays the linkage between genes and biological concepts as a network. The size of the dot corresponds to the number of DE genes belonging to the enriched GO pathway and the fold change shows the difference between DM-ILD relative to other diseases. Genes mentioned in the text are highlighted in parentheses.

Supplementary Figure 9

A Monocytes-macrophages in BALF in patients with RA, dot plot

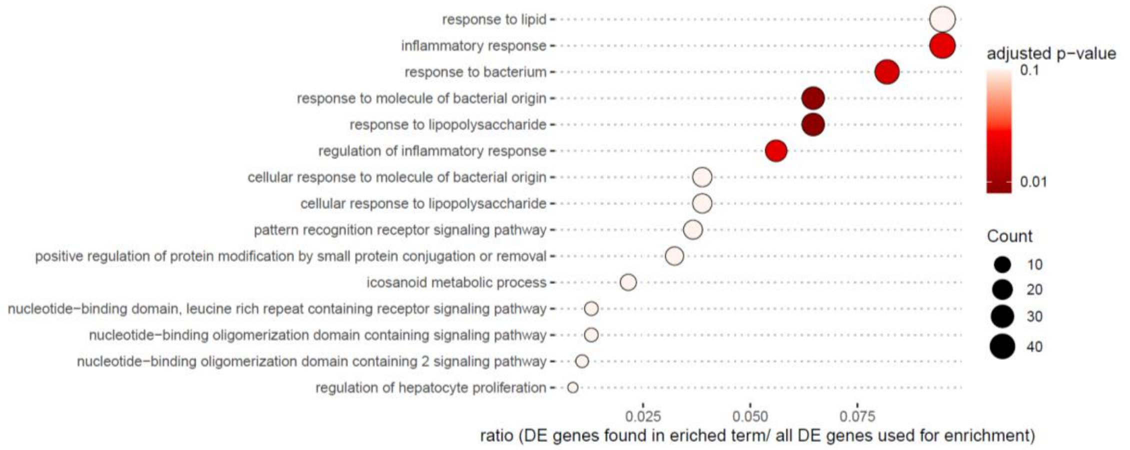

B Neutrophils in BALF in patients with RA, dot plot

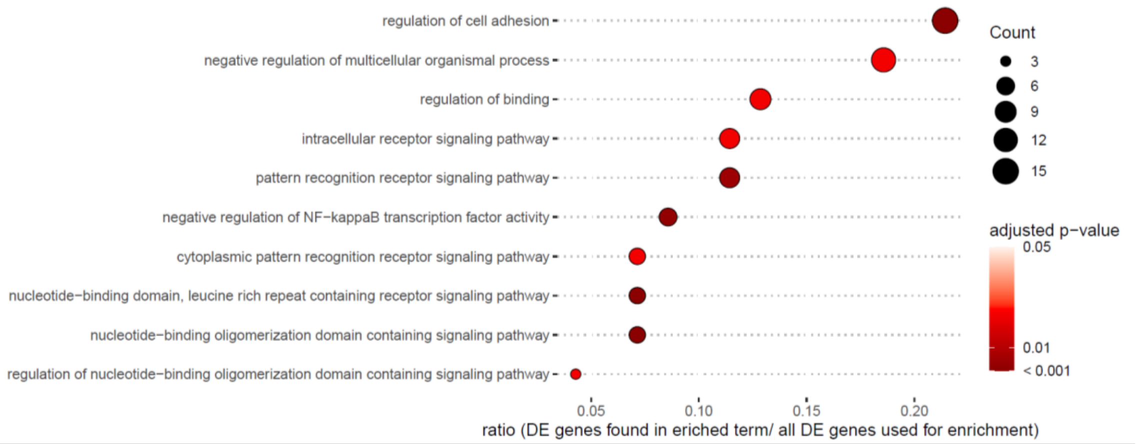

C Monocytes-macrophages in BALF in patients with RA, c-net

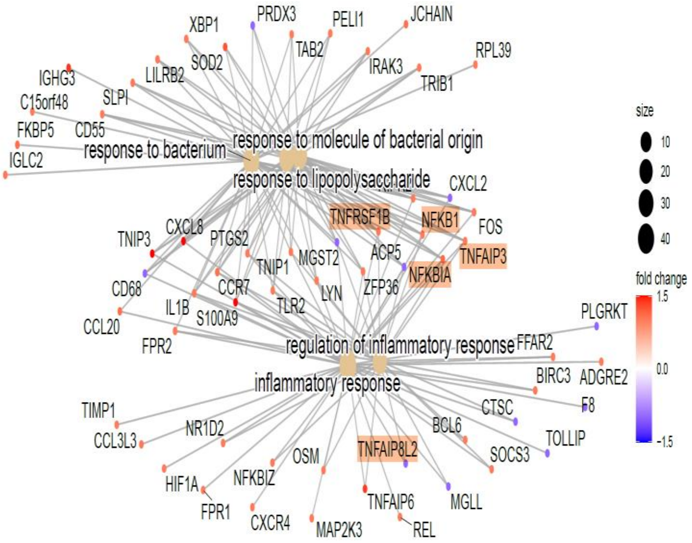

D Neutrophils in BALF in patients with RA, c-net

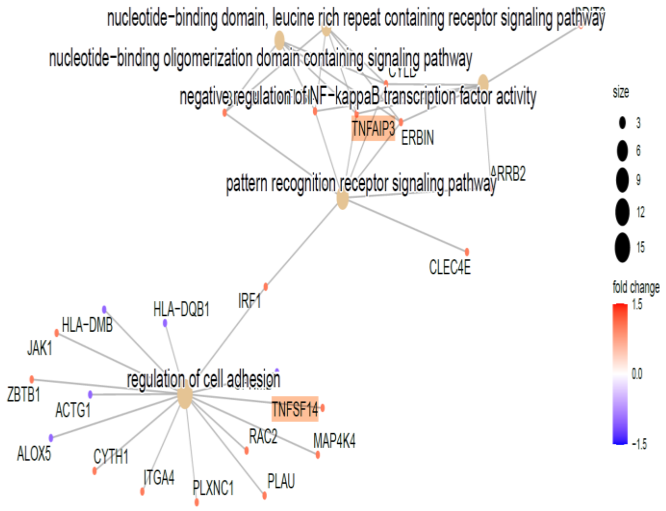

E B/plasma cells in BALF in patients with RA, dot plot

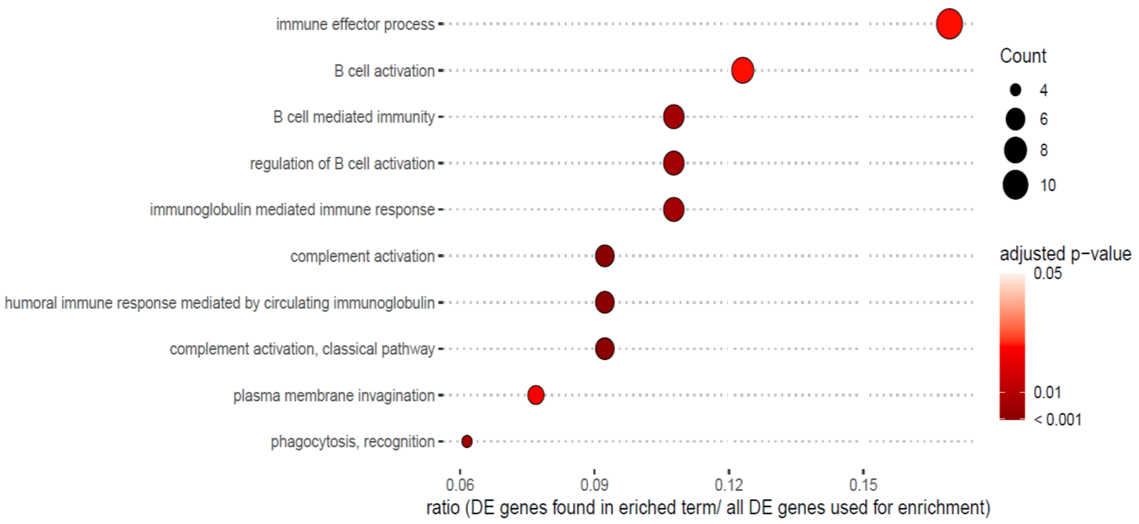

F B/plasma cells in BALF in patients with RA, c-net

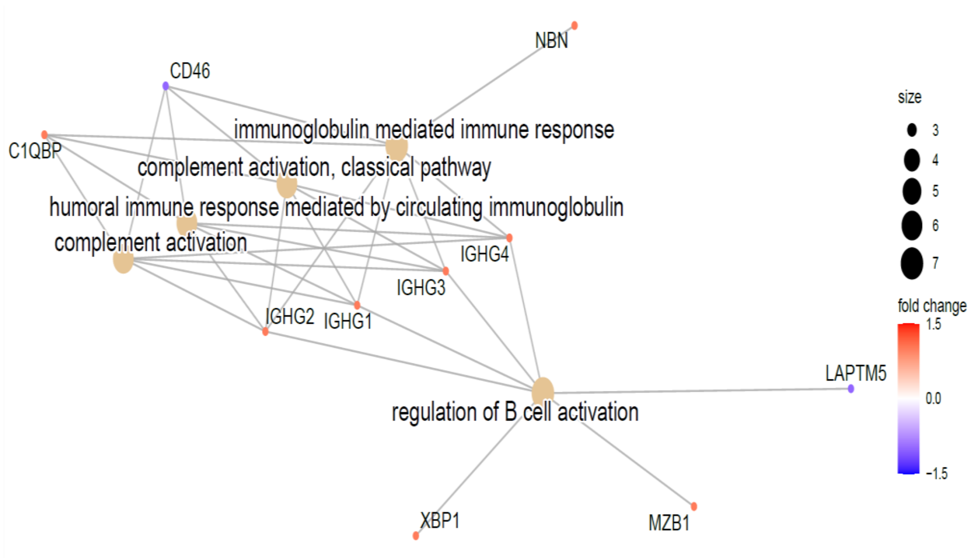

Gene ontology (GO) enrichment analysis of differentially expressed (DE) genes in the bronchoalveolar lavage fluid (BALF) cells from patients with rheumatoid arthritis (RA)-associated interstitial lung disease (ILD). The most significantly enriched pathways in each immune cell were visualized by dot plot or c-net. (A) Monocytes-macrophages in the BALF by dot plot. (B) Neutrophils in the BALF by dot plot. (C) Monocytes-macrophages in the BALF by c-net. (D) Neutrophils in the BALF by c-net. (E) B/plasma cells in the BALF by dot plot. (F) B/plasma cells in the BALF by c-net. The p-value cutoff for genes was set at 0.05. Dot plot shows enriched terms. The size of the dot corresponds to the gene count enriched in the pathway and the color of the dot indicates the pathway enrichment significance. C-net displays the linkage between genes and biological concepts as a network. The size of the dot corresponds to the number of DE genes belonging to the enriched GO pathway and the fold change shows the difference between RA-ILD relative to other diseases. Genes mentioned in the text are highlighted in parentheses.

Supplementary Figure 10

A B/plasma cells in blood in patients with SSc, dot plot

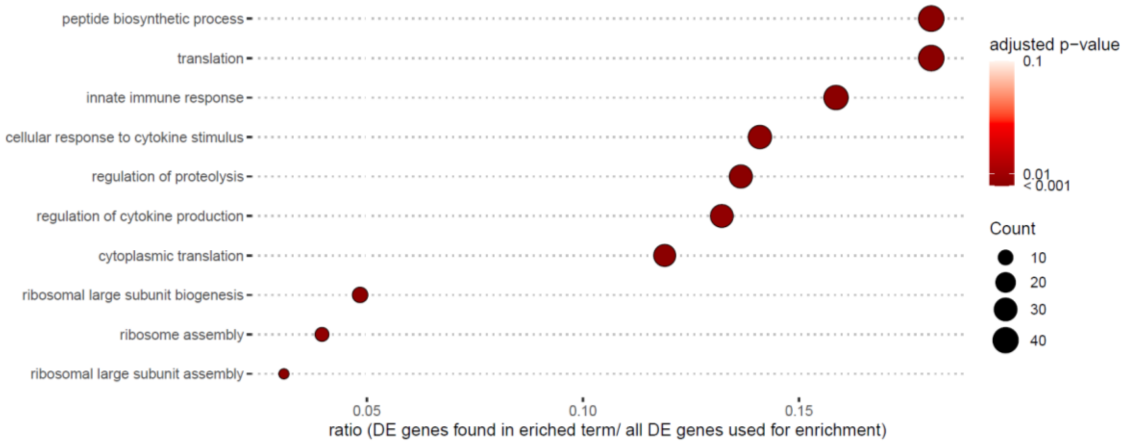

B B/plasma cells in blood in patients with SSc, c-net

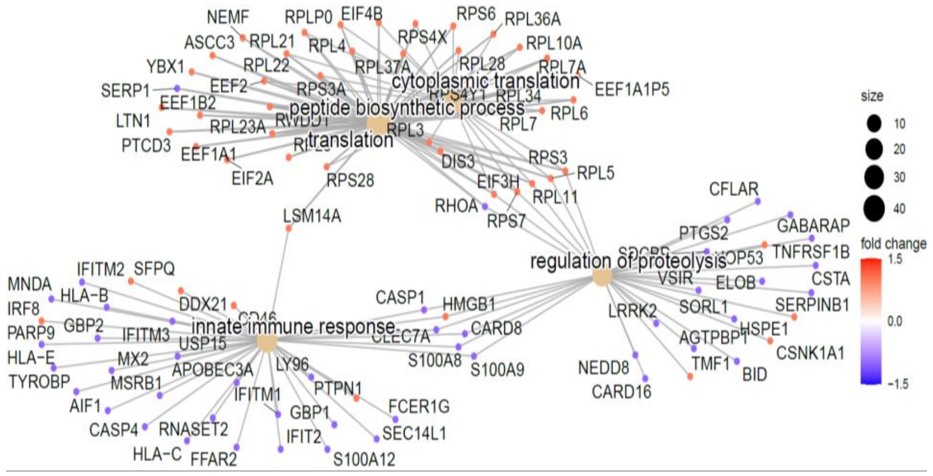

C Neutrophils in BALF in patients with SSc, dot plot

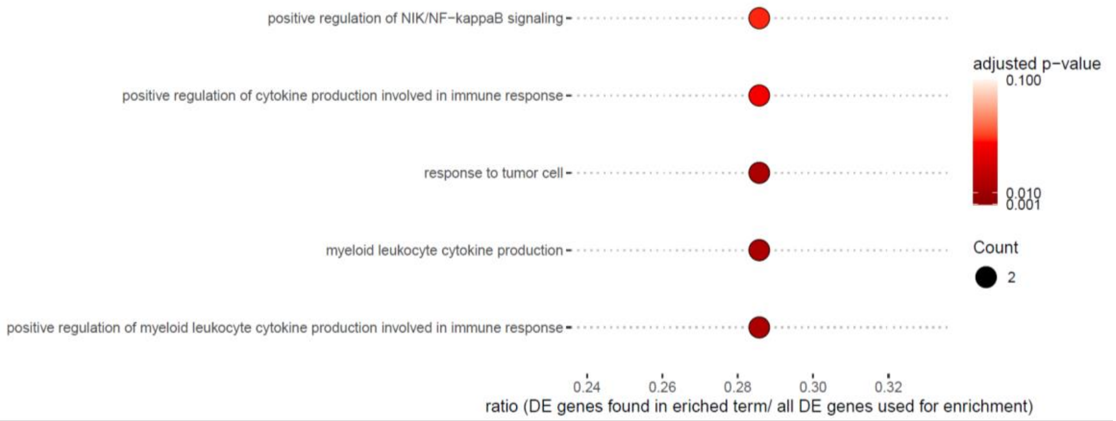

D Neutrophils in BALF in patients with SSc, c-net

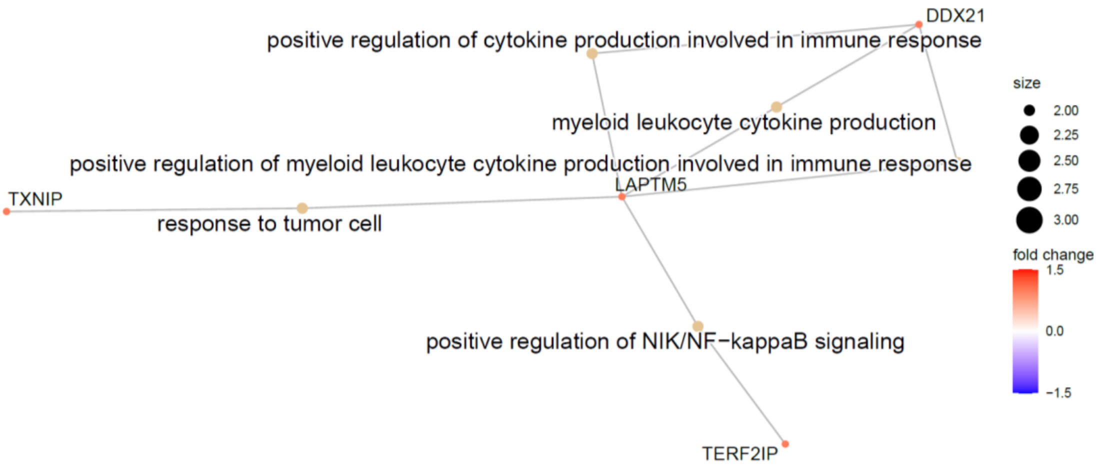

E Neutrophils in blood in patients with SSc, dot plot

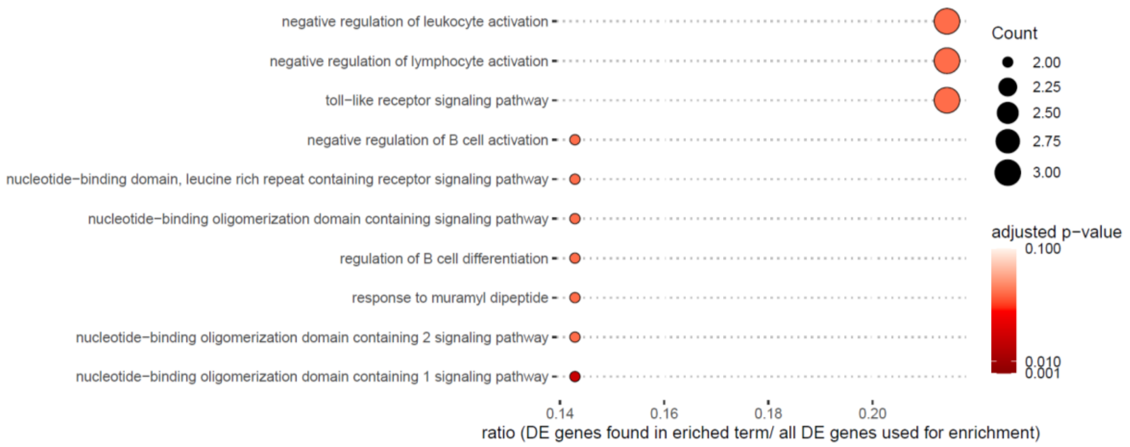

F Neutrophils in blood in patients with SSc, c-net

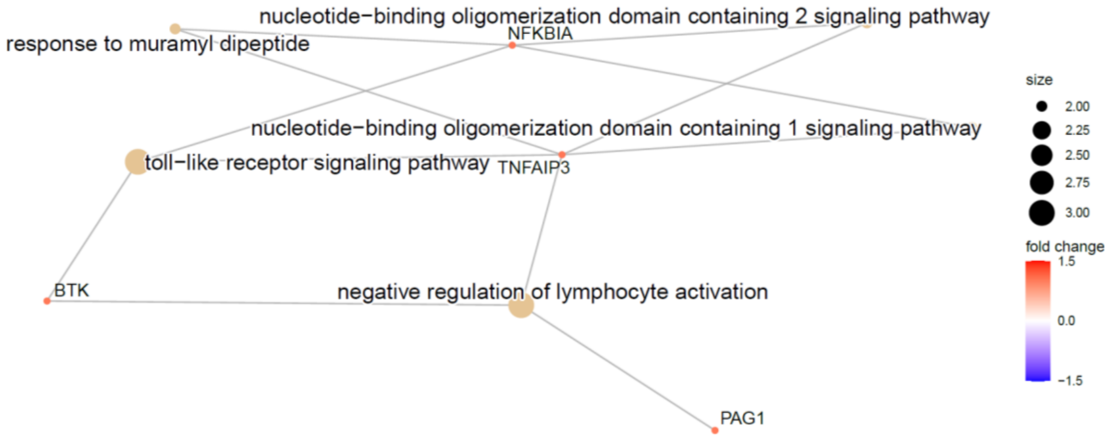

G Monocytes-macrophages in BALF in patients with SSc, dot plot

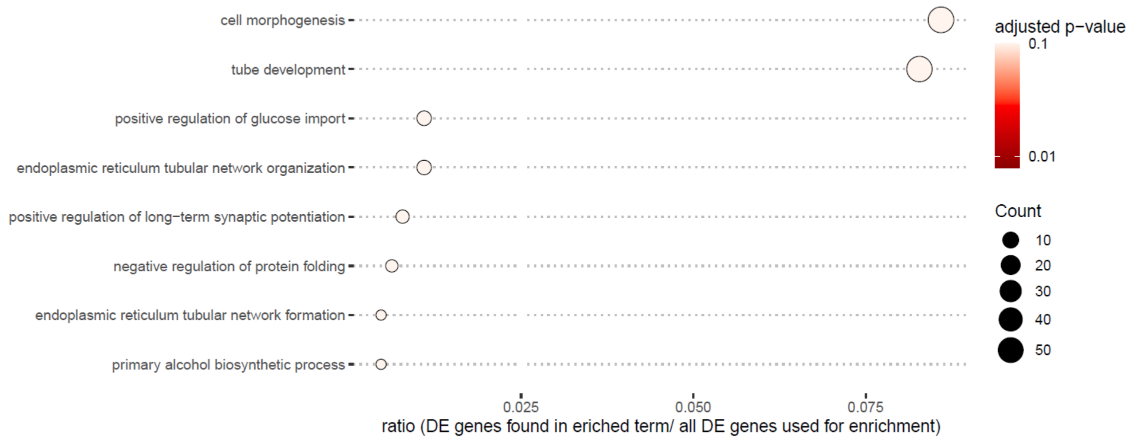

H Monocytes-macrophages in BALF in patients with SSc, c-net

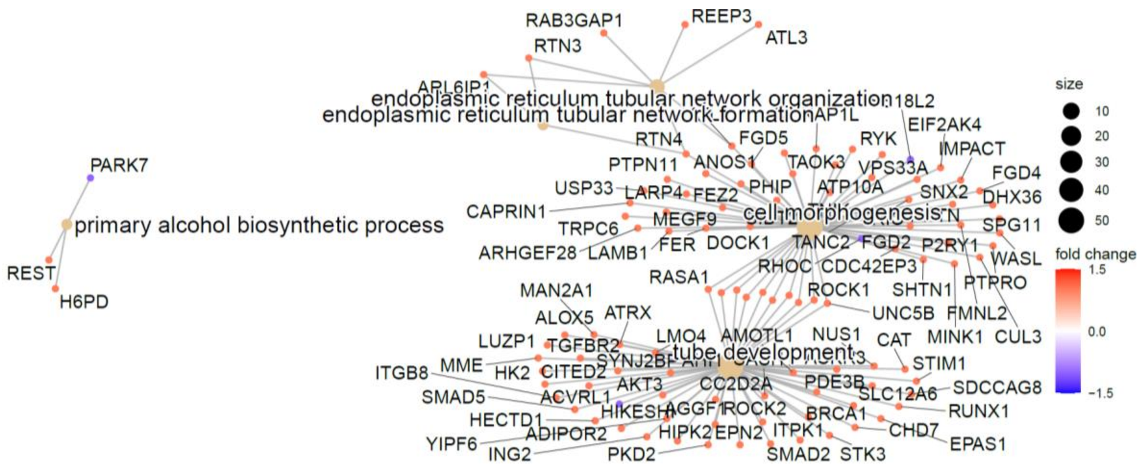

I Monocytes in blood in patients with SSc, dot plot

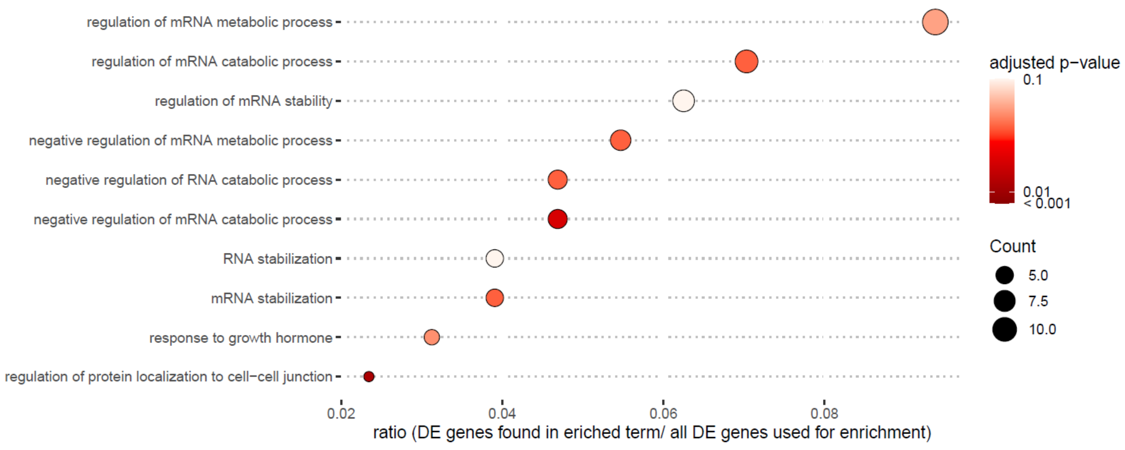

J Monocytes in blood in patients with SSc, c-net

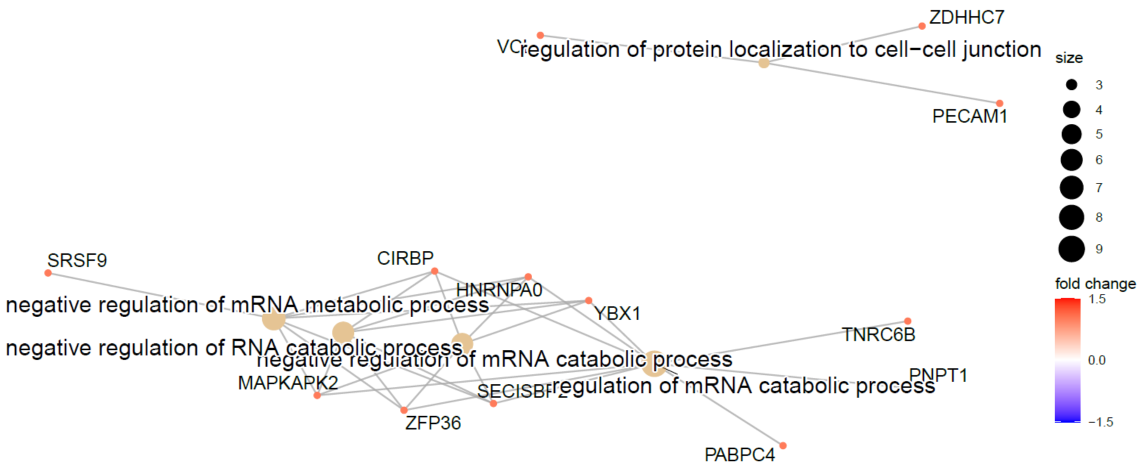

Gene ontology (GO) enrichment analysis of differentially expressed (DE) genes in the bronchoalveolar lavage fluid (BALF) cells and blood from patients with systemic sclerosis (SSc)-associated ILD. The most significantly enriched pathways in each immune cell were visualized by dot plot or c-net. (A) B/plasma cells in the blood by dot plot. (B) B/plasma cells in the blood by c-net. (C) Neutrophils in the BALF by dot plot. (D) Neutrophils in the BALF by c-net. (E) Neutrophils in the blood by dot plot. (F) Neutrophils in the blood by c-net. (G) Monocytes-macrophages in the BALF by dot plot. (H) Monocytes-macrophages in the BALF by c-net. (I) Monocytes in the blood by dot plot. (J) Monocytes in the blood by c-net. The p-value cutoff for genes was set at 0.01 for neutrophils in the BALF and blood and at 0.05 for B/plasma cells and monocytes in the blood and monocyte-macrophages in the BALF. Dot plot shows enriched terms. The size of the dot corresponds to the gene count enriched in the pathway and the color of the dot indicates the pathway enrichment significance. C-net displays the linkage between genes and biological concepts as a network. The size of the dot corresponds to the number of DE genes belonging to the enriched GO pathway and the fold change shows the difference between SSc-ILD relative to other diseases. Genes mentioned in the text are highlighted in parentheses.

Supplementary Figure 11

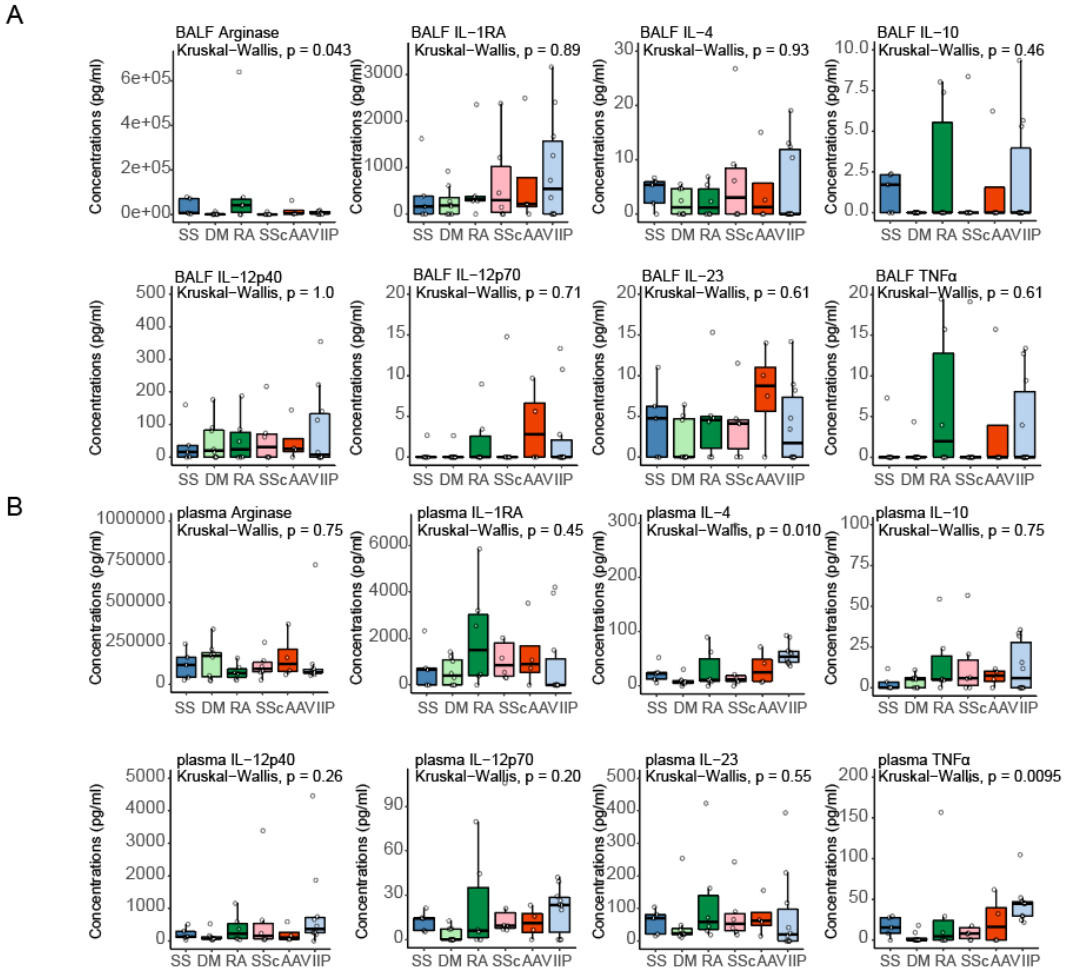

Comparison of cytokine and chemokine levels in the bronchoalveolar lavage fluid (BALF) supernatants and plasma among patients with various diseases. (A) Comparison in the BALF supernatants. (B) Comparison in the plasma. The Kruskal–Wallis test followed by the Steel–Dwass test was performed for multi-condition comparison. Statistical significance was inferred when $p < 0.05$.

The concentrations of IL-4 in the plasma of patients with dermatomyositis (DM)-associated interstitial lung disease (ILD) was significantly increased compared to patients with idiopathic interstitial pneumonia (IIP) ($p = 0.0051$). The concentrations of TNFα in the plasma of patients with DM-ILD was significantly increased compared to patients with IIP ($p = 0.0040$).

Abbreviations: SS, Sjögren’s syndrome; DM, dermatomyositis; RA, rheumatoid arthritis; SSc, systemic sclerosis; AAV, ANCA-associated vasculitis; IIP, idiopathic interstitial pneumonia; IL-1RA, Interleukin 1 receptor antagonist; IL, interleukin; TNFα, tumor necrosis factor-alpha.

Supplementary Figure 12

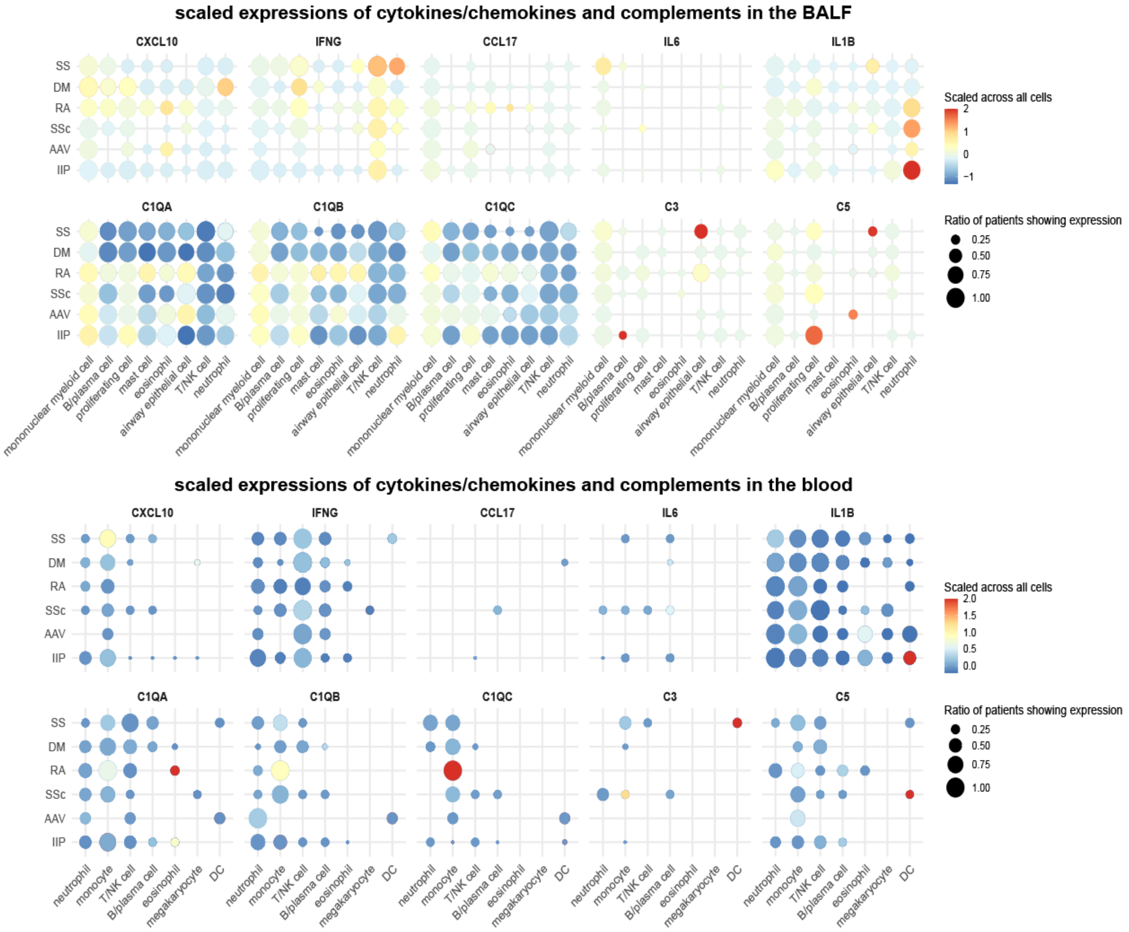

Comparison of differential expression of mRNAs related to several cytokines/chemokines and complements. Relative gene expression differences for each disease and each cell type are shown.

Abbreviations: SS, Sjögren’s syndrome; DM, dermatomyositis; RA, rheumatoid arthritis; SSc, systemic sclerosis; AAV, ANCA-associated vasculitis; IIP, idiopathic interstitial pneumonia; CXCL10, C-X-C Motif Chemokine Ligand 10; IFNG, Interferon Gamma; CCL17, C-C Motif Chemokine Ligand 17; TARC, Thymus and Activation-Regulated Chemokine; IL6, Interleukin 6; IL1B, Interleukin 1 Beta; C1QA, Complement C1q A Chain; C1QB, Complement C1q B Chain; C1QC, Complement C1q C Chain.

Supplementary Table 1

Patient number	Diagnosis	Radiological pattern	sex	age	BMI	current smoking (Y=yes, No=no)	EX-smoking (Y=yes, No=no)	Pack-years (number)	Medication before sample collections	Immunosuppressant before sample collections	Autoantibodies	ELISA analysis (Y=yes, No=no)	Sequence analysis of the blood cells (Y=yes, No=no)	Sequence analysis of the BALF cells (Y=yes, No=no)	Flow cytometry analysis of the blood cells (Y=yes, No=no)	Flow cytometry analysis of the BALF cells (Y=yes, No=no)
1	IIP	OP	male	64	22	N	N	0	None	None	anti-cardiolipin antibody, anti-β2-glycoprotein I antibody	N	N	N	N	N
2	DM	OP	male	56	27	N	Y	4	Atorvastatin Calcium Hydrate, Lisinopril Hydrate	None	anti-ARS antibody, anti-Jo1 antibody	Y	Y	Y	Y	Y
3	IIP	UIP	male	75	24	N	Y	90	Aspirin, Ethyl Icosapentate, Metformin Hydrochloride, Olmesartan Medoxomil, Pitavastatin Calcium Hydrate	None	anti-CCP antibody, anti-SSA antibody, rheumatoid factor	Y	Y	Y	Y	Y
4	RA	NSIP	female	87	27	N	N	0	Amlodipine Besilate, Magnesium Oxide	None	anti-CCP antibody, rheumatoid factor	Y	N	Y	Y	Y
5	RA	NSIP	male	66	30	N	Y	34	Azilsartan/ Amlodipine Besilate, Celecoxib, Entecavir Hydrate, Febuxostat, Glimepiride, Lansoprazole, Salazosulfapyridine, Sitagliptin Phosphate Hydrate/ Ipragliflozin L-Proline	None	Not detected	Y	Y	Y	Y	Y
6	SSc	NSIP	female	68	21	N	N	0	Fexofenadine Hydrochloride, L-Carbocisteine, Rebamipide	None	anti-Scl-70 antibody, anti-SSA antibody, rheumatoid factor	Y	Y	Y	Y	Y
7	DM	NSIP	male	64	22	N	N	0	None	None	anti-Jo1 antibody	Y	Y	Y	Y	Y
8	RA	OP	male	61	24	N	Y	25	Celecoxib, Rebamipide, Rosuvastatin Calcium	None	rheumatoid factor	Y	Y	Y	Y	Y
9	RA	OP	male	69	20	N	Y	11	Alendronate Sodium Hydrate, Alfacalcidol, Esomeprazole Magnesium Hydrate, Salazosulfapyridine, Sulfamethoxazole Trimethoprim	Prednisolone (5 mg/day)	anti-CCP antibody	Y	Y	Y	Y	Y
10	SSc	NSIP	male	68	22	Y	Y	43	Beraprost Sodium, Diltiazem Hydrochloride, Magnesium Oxide, Mosapride Citrate Hydrate, Pitavastatin Calcium Hydrate, Vonoprazan Fumarate	Prednisolone (7.5 mg/day), Mycophenolate Mofetil (2 g/day)	anti-Scl-70 antibody	N	N	N	N	N
11	SSc	UIP	female	74	22	N	Y	22	Acetaminophen, Azilsartan, Benidipine Hydrochloride, Fenofibrate, Metformin Hydrochloride, Vildagliptin, Zopiclone	None	anti-centromere antibody, rheumatoid factor	Y	Y	Y	Y	Y
12	DM	NSIP	male	45	17	N	N	0	None	None	anti-ARS antibody, anti-Jo1 antibody	Y	Y	Y	Y	Y
13	SS	UIP	female	68	24	N	N	0	Ambroxol Hydrochloride, Ethyl Loflazepate, Etizolam, Erythromycin Stearate, Mecobalamin, Sulpiride	None	anti-CCP antibody, anti-double stranded DNA antibody, anti-SSA antibody, anti-SSB antibody, anti-smith antibody, proteinase-3 ANCA, rheumatoid factor,	Y	Y	Y	Y	Y
14	SS	NSIP	female	43	16	N	N	0	None	None	anti-SSA antibody	Y	Y	Y	Y	Y
15	SS	NSIP	female	85	20	N	N	0	Amlodipine Besilate, Azosemide, Candesartan Cilexetil, Edoxaban Tosilate Hydrate, Metildigoxin	None	anti-centromere antibody, anti-SSA antibody	Y	Y	Y	Y	Y
16	IIP	OP	male	70	24	Y	Y	40	Atenolol, Benzbromarone, Brotizolam, Celecoxib, Etodolac, Irbesartan/ Amlodipine Besilate, Nitrazepam, Rabeprazole Sodium, Rebamipide	None	Not detected	N	N	N	N	N
17	SS	HP	female	55	15	N	N	0	Atorvastatin Calcium Hydrate, Mesalazine	None	anti-SSB antibody	Y	Y	Y	Y	Y
18	IIP	NSIP	female	63	26	Y	Y	70	None	None	rheumatoid factor	Y	Y	Y	Y	Y
19	DM	NSIP	female	44	25	N	N	0	None	None	Not detected	Y	Y	Y	Y	Y
20	DM	NSIP	male	79	25	N	Y	30	Amlodipine Besilate, Famotidine, Rebamipide, Vilanterol Trifenatate/ Fluticasone Furoate	None	anti-ARS antibody, rheumatoid factor	Y	Y	Y	Y	Y
21	IIP	UIP	male	71	25	N	Y	26	Alfacalcidol, Allopurinol	None	Not detected	Y	Y	Y	Y	Y
22	IIP	NSIP	male	78	28	N	Y	72	Benidipine Hydrochloride, Candesartan Cilexetil, Carvedilol, Clopidogrel Sulfate, Febuxostat, Fursultiamine Hydrochloride, Lansoprazole, Magnesium Oxide, Pitavastatin Calcium Hydrate	None	Not detected	Y	Y	Y	Y	Y
23	SS	NSIP	female	64	24	N	N	0	None	None	anti-CCP antibody, anti-SSA antibody, anti-SSB antibody, rheumatoid factor	Y	Y	Y	Y	Y
24	IIP	NSIP	female	72	23	N	Y	16	None	None	anti-ARS antibody, anti-Scl-70 antibody, rheumatoid factor	Y	Y	Y	N	Y
25	RA	NSIP	female	73	28	N	N	0	Azilsartan, Imidapril Hydrochloride	None	anti-CCP antibody, anti-SSA antibody, rheumatoid factor	Y	N	Y	Y	Y
26	IIP	NSIP	male	61	24	N	Y	38	Teneligliptin Hydrobromide Hydrate/ Canagliflozin Hydrate, Pravastatin Sodium	None	anti-ARS antibody	Y	Y	Y	Y	Y
27	IIP	NSIP	male	64	30	N	Y	49	Famotidine	None	Not detected	Y	Y	Y	Y	Y
28	IIP	PPFE	male	73	18	N	Y	40	None	None	Not detected	Y	Y	Y	Y	Y
30	IIP	NSIP	male	70	23	N	N	0	Acetaminophen, Amlodipine Besilate, Atorvastatin Calcium Hydrate, Magnesium Oxide	None	Not detected	Y	Y	Y	Y	Y
31	HP	UIP	female	84	27	N	Y	3	Fexofenadine Hydrochloride, L-Carbocisteine, Rebamipide	None	Not detected	N	N	N	N	N
32	SSc	NSIP	female	77	21	N	N	0	Atorvastatin Calcium Hydrate, Beraprost, Bisoprolol Fumarate, Bosentan Hydrate, Edoxaban Tosilate Hydrate, Famotidine, Hydrochlorothiazide, Losartan Potassium, Nifedipine, Sodium Risedronate Hydrate	None	Not detected	Y	Y	Y	Y	Y
33	IIP	UIP	male	75	30	Y	Y	50	Ambroxol Hydrochloride, Amlodipine Besilate, Aspirin, Epalrestat, Lansoprazole, Magnesium Oxide, Metformin Hydrochloride, Pitavastatin Calcium Hydrate, Telmisartan/ Amlodipine Besilate, Tiotropium Bromide Hydrate/ Tiotropium Bromide, Voglibose	None	Not detected	Y	Y	Y	Y	Y
34	AAV	UIP	male	73	19	Y	Y	48	None	None	myeloperoxidase ANCA, rheumatoid factor	Y	Y	Y	Y	Y
35	DM	NSIP	female	59	21	N	N	0	Betahistine Mesilate, Ethyl Loflazepate, Difenidol Hydrochloride, Loxoprofen Sodium Hydrate, Trichlormethiazide, Vonoprazan Fumarate	None	anti-ARS antibody, rheumatoid factor	Y	Y	Y	Y	Y
36	AAV	Unclassifiable	male	59	25	N	Y	40	Abiraterone Acetate, Degarelix Acetate, Denosumab	None	myeloperoxidase ANCA	Y	N	Y	Y	Y
39	RA	NSIP	male	78	19	N	Y	10	Loxoprofen Sodium Hydrate, Metformin Hydrochloride, Lansoprazole, Vildagliptin/ Metformin Hydrochloride	None	anti-CCP antibody, rheumatoid factor	Y	Y	Y	Y	Y
40	SSc	NSIP	female	68	25	N	N	0	Cloperastine Hydrochloride, L-Carbocisteine, Beraprost Sodium, Sarpogrelate Hydrochloride, Tocopherol Acetate, Vonoprazan Fumarate Mosapride Citrate Hydrate, Olopatadine Hydrochloride	None	anti-Scl-70 antibody, myeloperoxidase ANCA, rheumatoid factor	Y	Y	Y	Y	Y
41	AAV	Unclassifiable	femele	73	31	N	N	0	Irbesartan/ Amlodipine Besilate, Loxoprofen Sodium Hydrate, Olopatadine Hydrochloride, Omeprazole, Pranlukast Hydrate, Topiroxostat, Chlorphenesin Carbamate, Montelukast Sodium, Imidafenacin	None	myeloperoxidase ANCA	Y	Y	Y	Y	Y
42	AAV	Unclassifiable	female	76	24	N	N	0	Budesonide/ Formoterol Fumarate Hydrate	None	myeloperoxidase ANCA, rheumatoid factor	Y	Y	Y	Y	Y
43	DM	NSIP	female	59	19	N	N	0	Brotizolam	None	anti-ARS antibody, anti-SSA antibody	Y	Y	Y	Y	Y
44	DM	NSIP	male	37	26	N	N	0	None	None	anti-melanoma differentiation-associated 5 protein antibody	Y	Y	Y	Y	Y
45	SSc	NSIP	female	67	22	N	N	0	Epinastine Hydrochloride, Mecobalamin, Mexiletine Hydrochloride, Midodrine hydrochloride, Sennoside A ・B calcium, Sodium Ferrous Citrate, Precipitated Calcium Carbonate, Warfarin Potassium	None	anti-centromere antibody	Y	N	Y	Y	Y
46	SSc	NSIP	female	49	22	N	N	0	Beraprost, Mosapride Citrate Hydrate	None	anti-Scl-70 antibody	Y	Y	Y	Y	Y

Abbreviations: AAV, ANCA-associated vasculitis; DM, dermatomyositis; HP, hypersensitivity pneumonitis; IIP, idiopathic interstitial pneumonia; RA, rheumatoid arthritis; SS, Sjögren's syndrome; SSc, systemic sclerosis
NSIP, Non-specific interstitial pneumonia; OP, Organizing pneumonia; PPFE, Pleuroparenchymal fibroelastosis; UIP, Usual interstitial pneumonia; ELISA, enzyme-linked immunosorbent assay

Supplementary Table 2

antibodies

Panel	Antibody	Fluorochrome	Clone	Vendor	Identifier	amount for single use (µl/100 µl)
Myeloid cell panel	CD45	Amcyan	HI30	Biolegend	304032	3
Myeloid cell panel	CD3	PerCP/Cy5.5	UCHT1	BD Biosciences	557706	5
Myeloid cell panel	CD19	PerCP/Cy5.5	HIB19	Biolegend	302212	5
Myeloid cell panel	CD56	PerCP/Cy5.5	B159	BD Biosciences	557711	5
Myeloid cell panel	CD14	APC	HCD14	Biolegend	325620	3
Myeloid cell panel	CD16	PE/Cy7	3G8	Biolegend	302039	2
Myeloid cell panel	HLA-DR	Pacific-blue	L243	Biolegend	307630	3
Myeloid cell panel	CD66b	PE	G10F5	Thermo Fisher Scientific	12-0666-41	2
Myeloid cell panel	FcR Blocking Reagent			Miltenyi Biotec	130-059-901	5
Myeloid cell panel	Dead/Live	APC/Cy7		Thermo Fisher Scientific	L34967	0.1
Lymphoid cell panel	CD45	Amcyan	HI30	Biolegend	304050	3
Lymphoid cell panel	CD3	PerCP/Cy5.5	UCHT1	Biolegend	300420	3
Lymphoid cell panel	CD4	Pacific-blue	RPA-T4	Biolegend	300530	3
Lymphoid cell panel	CD8	APC	SK1	Biolegend	344730	3
Lymphoid cell panel	CD16	PE/Cy7	3G8	Biolegend	302039	2
Lymphoid cell panel	CD19	FITC	HIB19	Biolegend	302218	5
Lymphoid cell panel	CD56	PE	NCAM16.2	BD Biosciences	563041	5
Lymphoid cell panel	FcR Blocking Reagent			Miltenyi Biotec	130-059-901	5
Lymphoid cell panel	Dead/Live	APC/Cy7		Thermo Fisher Scientific	L34967	0.1

commercial assays

Commercial Assay	Vendor	Identifier
TapeStation HS D5000 Reagents (Sample Buffer & Ladder)	Agilent	Cat#5067-5593
High Sensitivity D5000 ScreenTape	Agilent	Cat#5067-5592
NEBNext High-Fidelity 2x PCR Master Mix	NEB	Cat#M0541L
Nextera XT DNA Library Preparation Kit (96 samples)	Illumina	Cat#FC-131-1096
2x Kapa Hifi HotStart Readymix	Kapa Biosystems	Cat#KK-2602
Exonuclease I	NEB	Cat#M0293S
Maxima H Minus Reverse Transcriptase	Thermo Fisher	Cat#EPO0753
dNTP	NEB	Cat#N04465
Human C3a ELISA Kit	BD Biosciences	Cat#550499
Human C4a ELISA Kit	BD Biosciences	Cat#550947
Human C5a ELISA Kit	BD Biosciences	Cat#557965
LEGENDplex Human Macrophage/Microglia Panel (13-plex)	Biolegend	Cat#740502

oligonucleotides

Oligonucleotide	Vendor	Identifier
TSO primer	IDT	custom
P5-SMART-PCR primer	IDT	custom
barcoded primer	IDT	custom
N70X Oligo	Illumina	custom
SMART PCR primer	Eurofins Scientific	Cat#74998995

further resources

Resource	Vendor	Identifier
AMPure XP beads	Beckman Coulter	Cat#A63881
LifterSlip™	Electron Microscopy Sc	Cat#72186-60
Polycarbonate (PCTE) membrane filters, 0.01 MICRON, 62MM	Sterlitech	Cat#PCT00162X22100
mRNA Capture beads	Chemgenes	Cat#MACOSKO-2011-10
Dow SYLGARD™ 184 Silicone Encapsulant Clear 0.5kg kit	Dow	184 SIL ELAST KIT 0.5KG
Transwell polycarbonate membrane cell culture inserts	Corning	Cat#3422

Supplementary Table 3

Supplemental information on preparation of Seq-Well arrays, Seq-Well libraries, and sequencing

APTES	0.05% APTES in 95% EtOH
PDITC buffers	0.2% PDITC, 10% pyridine, and 90% DMF
PGA buffer	20 µg/mL polyglutamic acid, 2 M NaCl, and 100 mM sodium carbonate (pH=10)
lysis buffer	5M guanidine thiocyanate, 1mM EDTA, 0.5% Sarkosyl, and 1% β-mercaptoethanol in H2O
hybridization buffer	2M NaCl, 3mM MgCl2, and 0.5% Tween-20 in PBS
washing buffer	2M NaCl, 3mM MgCl2, and 20mM Tris-HCl pH 8.0, in H2O
Maxima Reverse Transcriptase reaction	Maxima RT buffer, 4% Ficoll PM-400, 1mM dNTPs, 1U/µL RNase inhibitor, 2.5 µM template switch oligonucleotide (TSO) primer, and 10U/µL Maxima Reverse Transcriptase in H2O
TE buffer	10mM Tris-HCl pH 8.0 and 1mM EDTA in H2O
exonuclease reaction	ExoI buffer and 1U/µL ExoI in H2O
bead counting solution	10% PEG and 2.5 M NaCl
PCR reactions	2X KAPA HiFi Hotstart Readymix and 25 µM SMART PCR primer in H2O
amplification of reverse transcribed cDNA libraries	initial denaturation at 95°C for 3 min, followed by 4 cycles of denaturation at 98°C for 20 s, annealing at 65°C for 45 s, and extension at 72°C for 3 min,<br>followed by 12 cycles of denaturation at 98°C for 20 s, annealing at °C for 20 s, and extension at 72°C for 3 min, and final extension at 72°C for 5 min
cleaned with AMPure XP beads	5 min incubation with beads, followed by 3 min on the magnet, two washes with 80% EtOH, 5 min dry-out, elution with 13 µL H2O for 3 min, followed by 2 min on the magnet for collection of the eluent

Supplementary Table 4-1

An overview of the used packages and package versions

	package	version
1	enrichplot	1.14.2
2	miQC	1.2.0
3	SAVER	1.1.3
4	doParallel	1.0.17
5	iterators	1.0.14
6	doRNG	1.8.6
7	rngtools	1.5.2
8	ideas	1.0.0
9	apeglm	1.16.0
10	TopKLists	1.0.7
11	glmGamPoi	1.6.0
12	scater	1.22.0
13	scuttle	1.4.0
14	SingleCellExperiment	1.16.0
15	knitr	1.37
16	readxl	1.4.0
17	SoupX	1.5.2
18	ggnewscale	0.4.7
19	clusterProfiler	4.2.2
20	org.Hs.eg.db	3.14.0
21	AnnotationDbi	1.56.2
22	foreach	1.5.2
23	DESeq2	1.34.0
24	SummarizedExperiment	1.24.0
25	Biobase	2.54.0
26	MatrixGenerics	1.6.0
27	matrixStats	0.62.0
28	GenomicRanges	1.46.1
29	GenomeInfoDb	1.30.1
30	Iranges	2.28.0
31	Matrix.utils	0.9.8
32	S4Vectors	0.32.4
33	BiocGenerics	0.40.0
34	magrittr	2.0.2
35	cowplot	1.1.1
36	Matrix	1.5-3
37	patchwork	1.1.1
38	ggrepel	0.9.1
39	ggpubr	0.4.0
40	RColorBrewer	1.1-3
41	pheatmap	1.0.12
42	harmony	0.1.0
43	Rcpp	1.0.8.3
44	janitor	2.1.0
45	forcats	0.5.1
46	dplyr	1.0.8
47	purrr	0.3.4
48	readr	2.1.2
49	tidyr	1.2.0
50	tibble	3.1.6
51	tidyverse	1.3.1
52	scales	1.2.0
53	pals	1.7
54	useful	1.2.6
55	ggplot2	3.4.0
56	stringr	1.4.0
57	ggrastr	1.0.1
58	data.table	1.14.2
59	SeuratObject	4.0.4
60	Seurat	4.1.0
61	DropletUtils	1.14.2

Supplementary Table 4-2

loaded via a namespace (and not attached):

	package	version
1	mixtools	2.0.0
2	graphlayouts	0.8.4
3	pbapply	1.5-0
4	lattice	0.20-45
5	haven	2.5.0
6	vctrs	0.5.1
7	usethis	2.1.5
8	mgcv	1.8-39
9	rmutil	1.1.10
10	blob	1.2.3
11	survival	3.3-1
12	spatstat.data	2.2-0
13	later	1.3.0
14	nloptr	2.0.3
15	DBI	1.1.2
16	uwot	0.1.11
17	jpeg	0.1-10
18	zlibbioc	1.40.0
19	MatrixModels	0.5-1
20	fBasics	4021.93
21	timeSeries	4021.105
22	htmlwidgets	1.5.4
23	mvtnorm	1.1-3
24	future	1.25.0
25	leiden	0.3.9
26	irlba	2.3.5
27	tidygraph	1.2.2
28	KernSmooth	2.23-20
29	promises	1.2.0.1
30	DelayedArray	0.20.0
31	vegan	2.6-4
32	pkgload	1.2.4
33	Hmisc	4.7-2
34	fs	1.5.2
35	MiRKAT	1.2.2
36	brio	1.1.3
37	fastmatch	1.1-3
38	digest	0.6.29
39	png	0.1-7
40	sctransform	0.3.3
41	scatterpie	0.1.8
42	DOSE	3.20.1
43	ggraph	2.1.0
44	pkgconfig	2.0.3
45	GO.db	3.14.0
46	spatstat.random	2.2-0
47	DelayedMatrixStats	1.16.0
48	ggbeeswarm	0.6.0
49	minqa	1.2.5
50	emdbook	1.3.12
51	reticulate	1.24
52	beeswarm	0.4.0
53	modeltools	0.2-23
54	xfun	0.30
55	zoo	1.8-10
56	tidyselect	1.1.2
57	reshape2	1.4.4
58	kernlab	0.9-30
59	ica	1.0-2
60	viridisLite	0.4.0

	package	version
61	pkgbuild	1.3.1
62	rlang	1.0.6
63	glue	1.6.2
64	modelr	0.1.8
65	ggsignif	0.6.4
66	SparseM	1.81
67	httpuv	1.6.5
68	BiocNeighbors	1.12.0
69	grr	0.9.5
70	DO.db	2.9
71	annotate	1.72.0
72	jsonlite	1.8.0
73	Xvector	0.34.0
74	bit	4.0.4
75	mime	0.12
76	gridExtra	2.3
77	gplots	3.1.3
78	stringi	1.7.6
79	processx	3.5.2
80	spatstat.sparse	2.1-1
81	scattermore	0.8
82	yulab.utils	0.0.6
83	bitops	1.0-7
84	cli	3.6.0
85	maps	3.4.0
86	RSQLite	2.2.20
87	spatial	7.3-15
88	rstudioapi	0.13
89	nlme	3.1-155
90	qvalue	2.26.0
91	locfit	1.5-9.7
92	listenv	0.8.0
93	miniUI	0.1.1.1
94	gridGraphics	0.5-1
95	stable	1.1.6
96	dbplyr	2.1.1
97	segmented	1.6-2
98	sessioninfo	1.2.2
99	lifecycle	1.0.3
100	timeDate	3043.102
101	transport	0.12-2
102	munsell	0.5.0
103	cellranger	1.1.0
104	mapproj	1.2.8
105	statip	0.2.3
106	caTools	1.18.2
107	codetools	0.2-18
108	coda	0.19-4
109	vipor	0.4.5
110	lmtest	0.9-40
111	htmlTable	2.4.1
112	xtable	1.8-4
113	ROCR	1.0-11
114	BiocManager	1.30.16
115	abind	1.4-5
116	farver	2.1.0
117	parallelly	1.31.1
118	RANN	2.6.1
119	aplot	0.1.9
120	CompQuadForm	1.4.3

	package	version
121	ggtree	3.2.1
122	GUniFrac	1.7
123	RcppAnnoy	0.0.19
124	goftest	1.2-3
125	dichromat	2.0-0
126	cluster	2.1.2
127	future.apply	1.9.0
128	tidytree	0.4.2
129	ellipsis	0.3.2
130	prettyunits	1.1.1
131	lubridate	1.8.0
132	ggridges	0.5.3
133	reprex	2.0.1
134	igraph	1.3.1
135	fgsea	1.20.0
136	remotes	2.4.2
137	testthat	3.1.2
138	spatstat.utils	2.3-0
139	htmltools	0.5.2
140	yaml	2.3.5
141	utf8	1.2.2
142	plotly	4.10.0
143	XML	3.99-0.9
144	foreign	0.8-82
145	withr	2.5.0
146	fitdistrplus	1.1-8
147	BiocParallel	1.28.3
148	bit64	4.0.5
149	Biostrings	2.62.0
150	spatstat.core	2.4-2
151	GOSemSim	2.20.0
152	rsvd	1.0.5
153	ScaledMatrix	1.2.0
154	devtools	2.4.3
155	memoise	2.0.1
156	evaluate	0.15
157	geneplotter	1.72.0
158	tzdb	0.3.0
159	permute	0.9-7
160	callr	3.7.0
161	ps	1.6.0
162	fansi	1.0.2
163	tensor	1.5
164	checkmate	2.1.0
165	cachem	1.0.6
166	desc	1.4.1
167	interp	1.1-3
168	deldir	1.0-6
169	rstatix	0.7.1
170	clue	0.3-64
171	rprojroot	2.0.2
172	tools	4.1.3
173	stabledist	0.7-1
174	Rcurl	1.98-1.6
175	car	3.1-1
176	ape	5.6-2
177	ggplotify	0.1.0
178	xml2	1.3.3
179	httr	1.4.2
180	assertthat	0.2.1

	package	version
181	rmarkdown	2.14
182	boot	1.3-28
183	globals	0.14.0
184	R6	2.5.1
185	nnet	7.3-17
186	genefilter	1.76.0
187	KEGGREST	1.34.0
188	treeio	1.18.1
189	gtools	3.9.2
190	statmod	1.5.0
191	beachmat	2.10.0
192	BiocSingular	1.10.0
193	splines	4.1.3
194	snakecase	0.11.0
195	carData	3.0-5
196	ggfun	0.0.9
197	colorspace	2.0-3
198	generics	0.1.2
199	base64enc	0.1-3
200	pillar	1.7.0
201	tweenr	2.0.2
202	GenomeInfoDbData	1.2.7
203	plyr	1.8.7
204	gtable	0.3.0
205	bdsmatrix	1.3-6
206	rvest	1.0.2
207	latticeExtra	0.6-30
208	shadowtext	0.1.2
209	fastmap	1.1.0
210	modeest	2.4.0
211	pscl	1.5.5
212	flexmix	2.3-18
213	quantreg	5.94
214	broom	0.8.0
215	backports	1.4.1
216	PearsonDS	1.2.3
217	lme4	1.1-31
218	hms	1.1.1
219	ggforce	0.4.1
220	Rtsne	0.16
221	shiny	1.7.1
222	polyclip	1.10-0
223	grid	4.1.3
224	numDeriv	2016.8-1.1
225	bbmle	1.0.25
226	lazyeval	0.2.2
227	Formula	1.2-4
228	crayon	1.5.0
229	MASS	7.3-55
230	downloader	0.4
231	sparseMatrixStats	1.6.0
232	viridis	0.6.2
233	rpart	4.1.16
234	compiler	4.1.3
235	spatstat.geom	2.4-0
